# Cyanine-based near infra-red organic photoredox catalysis†

**DOI:** 10.1039/d1sc00998b

**Published:** 2021-04-13

**Authors:** Anne Roly Obah Kosso, Nicolas Sellet, Alexandre Baralle, Morgan Cormier, Jean-Philippe Goddard

**Affiliations:** Laboratoire d’Innovation Moléculaire et Applications (LIMA), UMR 7042, Université de Haute-Alsace (UHA), Université de Strasbourg, CNRS 68100 Mulhouse France morgan.cormier@uha.fr jean-philippe.goddard@uha.fr

## Abstract

Direct metal-free near infra-red photoredox catalysis is applied to organic oxidation, photosensitization and reduction, involving cyanines as photocatalysts. This photocatalyst is competitive with conventional reactions catalyzed under visible light. Kinetic and quenching experiments are also reported. Interestingly, these systems are compatible with water media, opening perspective for various applications.

## Abstract 2

Direct metal-free near infra-red photoredox catalysis is applied to oxidation, reduction and photosensitization, involving cyanines as photocatalysts. Mechanistic insights through kinetic and quenching experiments are also reported.

## Graphical abstract

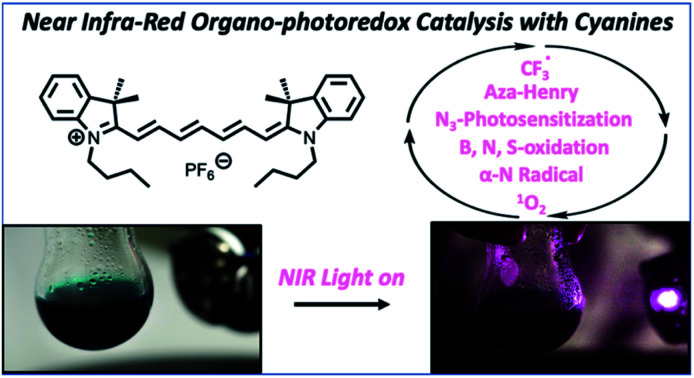

## Introduction

**Scheme 1:**
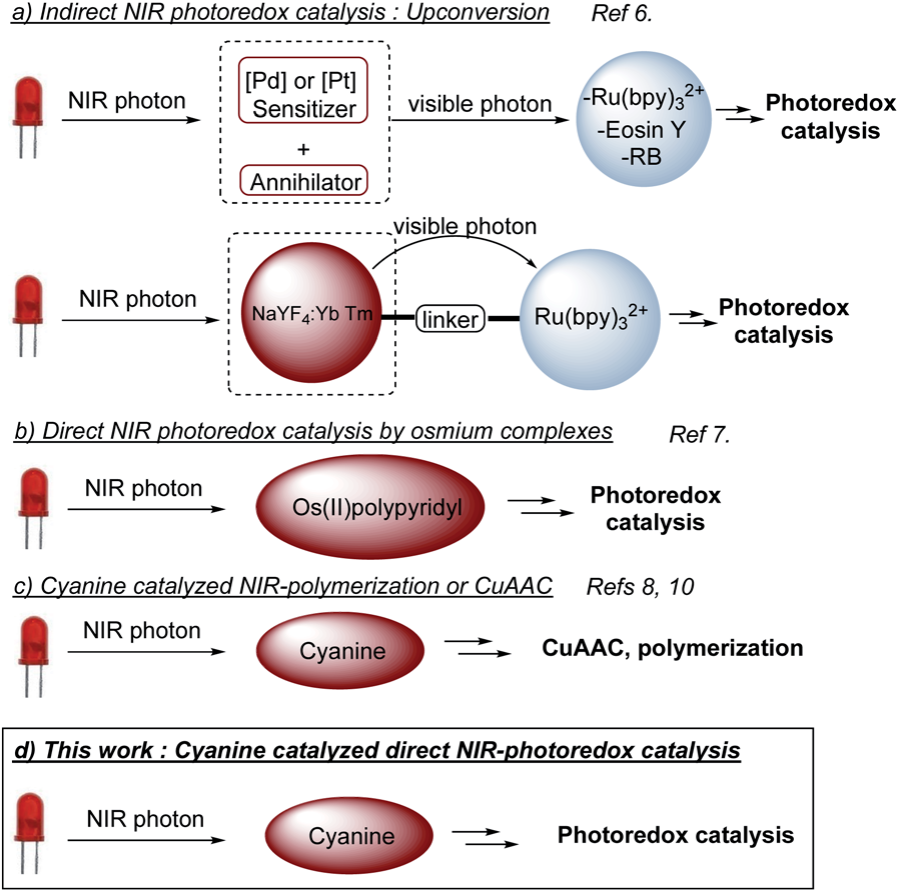
NIR-Photoredox catalysis.

Light as a source of energy for organic transformations has always been an interesting alternative to thermally driven processes. Classically, UV-irradiation is used to activate organic reaction, since most of molecules absorb in this spectral window.^1^ However, such high energetic light is not innocent and limit the reaction applicability in terms of scope and safety issues. Moving to less energetic wavelengths (*i.e.* visible light), photoredox catalysis appeared to be a remarkable solution.^2^ The excitation of a photoredox catalyst (PC to PC*) could give birth to either an oxidant or a reductant, able to promote SET organic transformations. Although extensively studied, the use of visible light has still some limitations like the low penetration of such wavelengths through the solution,^3^ which limits the set-up to small scales or flow-systems.^4^ Additionally, the biological window for light is between 650 nm and 950 nm,^5^ which reduces the impact of visible photoredox catalysis for biological applications. To circumvent these problems, longer wavelengths should be involved, shifting from visible light to near infra-red (NIR) irradiation. Very recently, NIR-photoredox catalysis emerged as a valuable solution resulting in more efficient photochemical processes, due to deeper penetration in various reaction media and the improvement of the irradiation surface. An appropriate photocatalyst able to absorb NIR-light with suitable excited state redox properties is needed. Indirect NIR-photoredox catalysis transformations have been developed based on upconversion phenomena. Two photocatalytic systems are needed, one to convert a NIR-photon into a visible photon and a second, based on conventional photocatalysts, absorbing visible photon to initiate a photoredox transformation (Scheme 1a).^6^ Recently, direct NIR-photoredox catalysis has been accomplished using Os(ii) polypyridyl complexes to promote oxidation/reduction reactions (Scheme 1b).^7^ Although having remarkable properties, preparation and handling need a glovebox. Moreover, osmium could be an issue in term of toxicity. In parallel, publications have been reported on NIR-polymerization using cyanines as a photoiniator (Scheme 1c).^3,8^ These NIR-dyes are also well-known as fluorescence probes^9^ and redox partners for CuAAC, also in a context of polymer science.^8,10^ Thus, we anticipated that such cyanines could catalyze organic photoredox transformations under NIR light and open a promising field of investigation to push forward the limitation of conventional visible light photoredox catalysis.

## Results and discussion

**Fig. 1:**
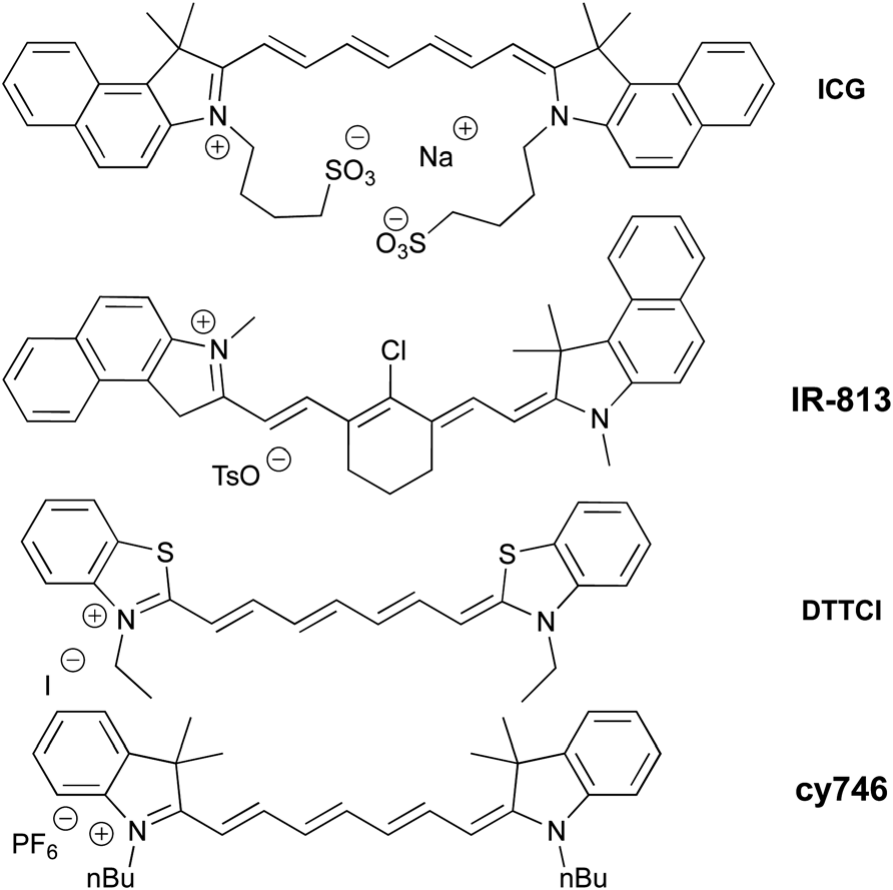
Selected cyanine photocatalysts.

Among a large variety of cyanines, we selected stable commercial Indocyanine Green (ICG), IR-813, DTTCI, cy746 (Fig. 1), with a structural diversity impacting their photophysical properties (see spectra in ESI §2†). However, this family of dyes owns similar excited state lifetime (∼1 ns)^11^ and comparable redox properties.^12^

Then, we began their evaluation with the photoredox oxidation of amine through the aza-Henry reaction (Table 1).^13^ The optimization has been done with **1a** and nitromethane as nucleophile to generate **2a** under NIR-light. Gratifyingly, the irradiation (810 nm) of **1a** (*E*_ox_ = 0.62 V *vs.* SCE)^14^ with photocatalysts in nitromethane (Table 1, entry 1–4) promoted the formation of **2a** in modest to good conversions. Among the tested cyanine photocatalysts, cy746 revealed to be superior (Table 1, entry 4) and we monitored the impact of the irradiation wavelength. As *λ*_max_ (cy746 in DMSO) = 760 nm, we expected to improve the reaction efficiency with an irradiation at 780 nm but only 14% conversion after 24 h was observed (Table 1, entry 5). No benefit was gained with lower energetic light at 940 nm (Table 1, entry 6).

**Table 1:** Aza-Henry reaction optimization

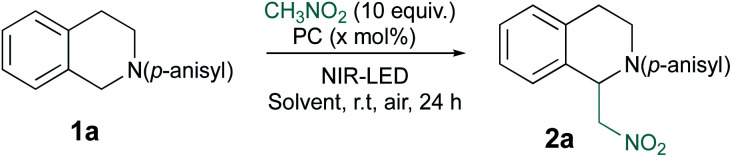					
Entry^a^	PC	*x* (mol%)	Solvent	*λ* (nm)	Conv^b^. (%)
1	ICG	10	CH_3_NO_2_^c^	810	16
2	IR-813	10	CH_3_NO_2_^c^	810	28
3	DTCCI	10	CH_3_NO_2_^c^	810	14
4	cy746	10	CH_3_NO_2_^c^	810	57
5	cy746	10	CH_3_NO_2_^c^	780	14
6	cy746	10	CH_3_NO_2_^c^	940	<5
7	cy746	10	DCM	810	48
8	cy746	10	DMF	810	n.r
9	cy746	10	MeOH	810	28
10	cy746	10	DMSO	810	91
11	cy746	5	DMSO	810	85
12	cy746	1	DMSO	810	67
13	cy746	—	DMSO	810	16
14^d^	cy746	5	DMSO	810	n.r
15^e^	cy746	5	DMSO	810	5

a Run on 0.1 mmol scale.

b Conversions are determined on the crude by ^1^H-NMR.

c CH_3_NO_2_ is used as solvent.

d Run in the dark.

e Run under nitrogen atmosphere in the absence of air.

Finally, variation of solvents and catalyst loadings (Table 1, entry 7–12) revealed that reaction conditions involving 10 equivalents of nitromethane in DMSO under irradiation (810 nm) allowed a very high 91% conversion after 24 h (Table 1, entry 10), which can be maintained at 85% with 5 mol% of cy746 (Table 1, entry 11). When the catalyst was omitted, only 16% conversion was measured (Table 1, entry 13) while no reaction occurred in the dark (Table 1, entry 14). In the absence of oxygen, 5% conversion was observed, indicating that photocatalyst could not be recovered without oxygen (Table 1, entry 15). This clearly indicates the synergistic effect of light, oxygen and photocatalyst to reach high conversion.

**Scheme 2:**
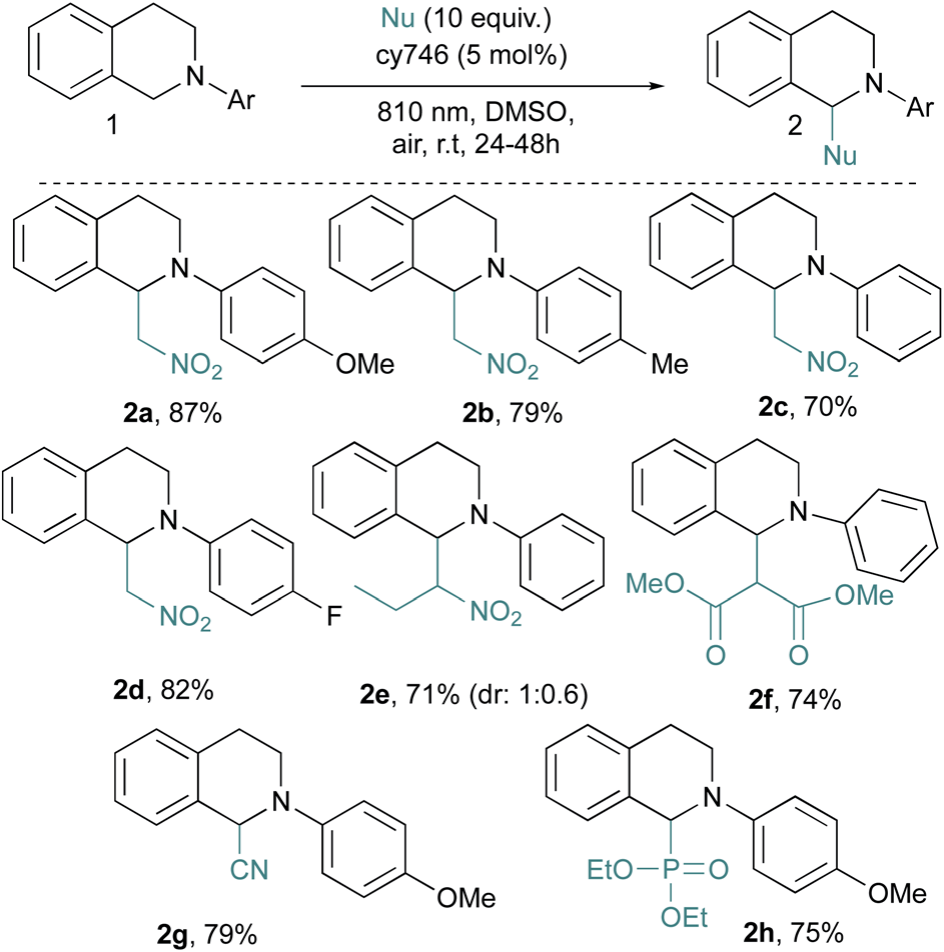
Scope of Aza-Henry type reactions.

**Scheme 3:**
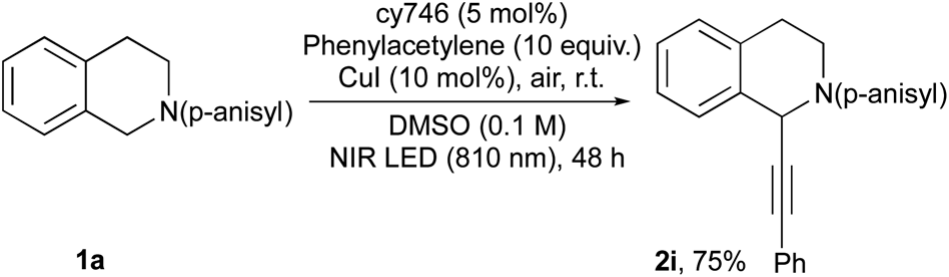
Dual catalysis: alkynylation reaction.

These optimal conditions were applied to a set of tetrahydroisoquinolines **1** with various nucleophiles (Scheme 2). An electronic modification of the *N*-aryl moiety is tolerated given **2a–d** in good yields even with more challenging electron poor substrates (*i.e.*, **2d**). A more sterically congested nitropropane does not affect the reaction and **2e** is obtained in good yield (71%, d.r = 1 : 0.6). Other C–C and C–P bond formation processes have been investigated.^14^ With dimethyl malonate, **2f** is isolated in 74% yield, resulting from the addition of the corresponding enol tautomer onto the iminium intermediate. In the same vein, cyanide anion addition from trimethylsilyl cyanide generates **2g** in 79% yield. Diethyl phosphite is also compatible with our optimized conditions and phosphonate **2h** is isolated in a satisfactory 75% yield. Our optimized conditions demonstrated a very good tolerance to the catalytic alkynylation of **1a** through copper phenylacetylide addition (Scheme 3). The photocatalytic system seems to be undisturbed by this second organometallic catalytic cycle since the corresponding adduct **2i** is obtained in 75% yield. In this dually catalyzed transformation, copper(i) intermediate as well as its reactivity, seems to be preserved against redox processes as it was also demonstrated for visible light photoredox catalysis.

**Fig. 2:**
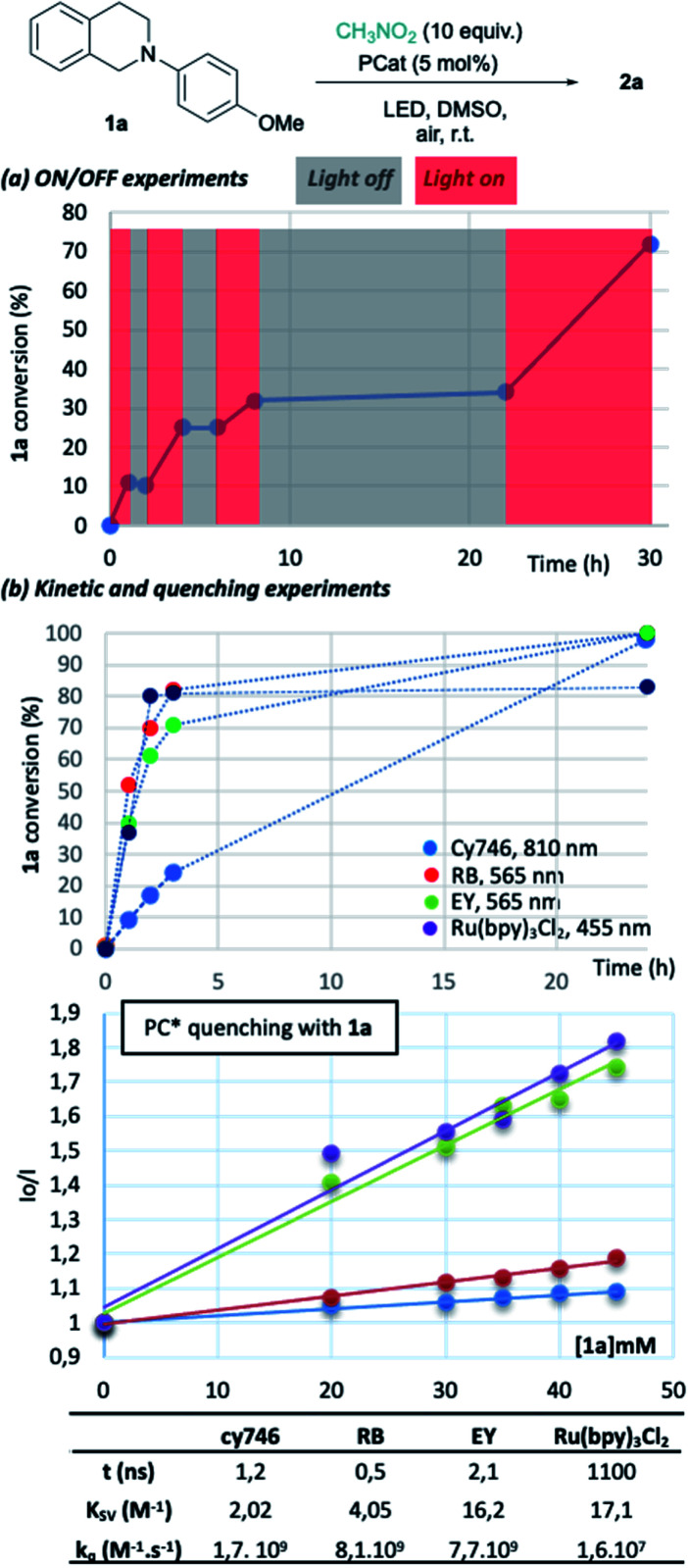
Reaction insights of NIR photoredox process.

To gain insights into the reaction parameters, additional experiments were done. “On/off” experiments demonstrated that the conversion increases during a light-on period and stopped when light is off (Fig. 2a). When the light was switched on after 14 h in the dark, the conversion of **1a** was resumed. This demonstrates the stability of the catalyst which was confirmed by the monitoring (^1^H-NMR) of an irradiated solution of cy746 (DMSO-d_6_) over 24 h (see ESI §5†).

We compared the kinetic profile of cy746 (810 nm) with Rose Bengal (565 nm), Eosin Y (565 nm) and Ru(bpy)_3_Cl_2_ (455 nm) in their respective optimal conditions for the formation of **2a** (Fig. 2b). The visible photocatalysts showed faster formation of **2a** to a plateau (∼80%), reached in 4 h. While the reaction with cy746 appeared to be slower (24% conversion in 3 h), full conversion was achieved in 24 h.

In addition, quenching experiments have been done to determine Stern–Volmer constant of the cy746/**1a** system in comparison with the same three catalysts (Fig. 2b).^15^ cy746 has comparable excited lifetime^11^ to other organic dyes (∼1 ns)^2*d*,16^ and similar excited state quenching constant (*k*_q_ = 1.7 10^9^ M^−1^ s^−1^) which is 100-fold more than Ru(bpy)_3_Cl_2_. Therefore, the slower reactions, observed with cy746 cannot be only explained by those two parameters.

**Scheme 4:**
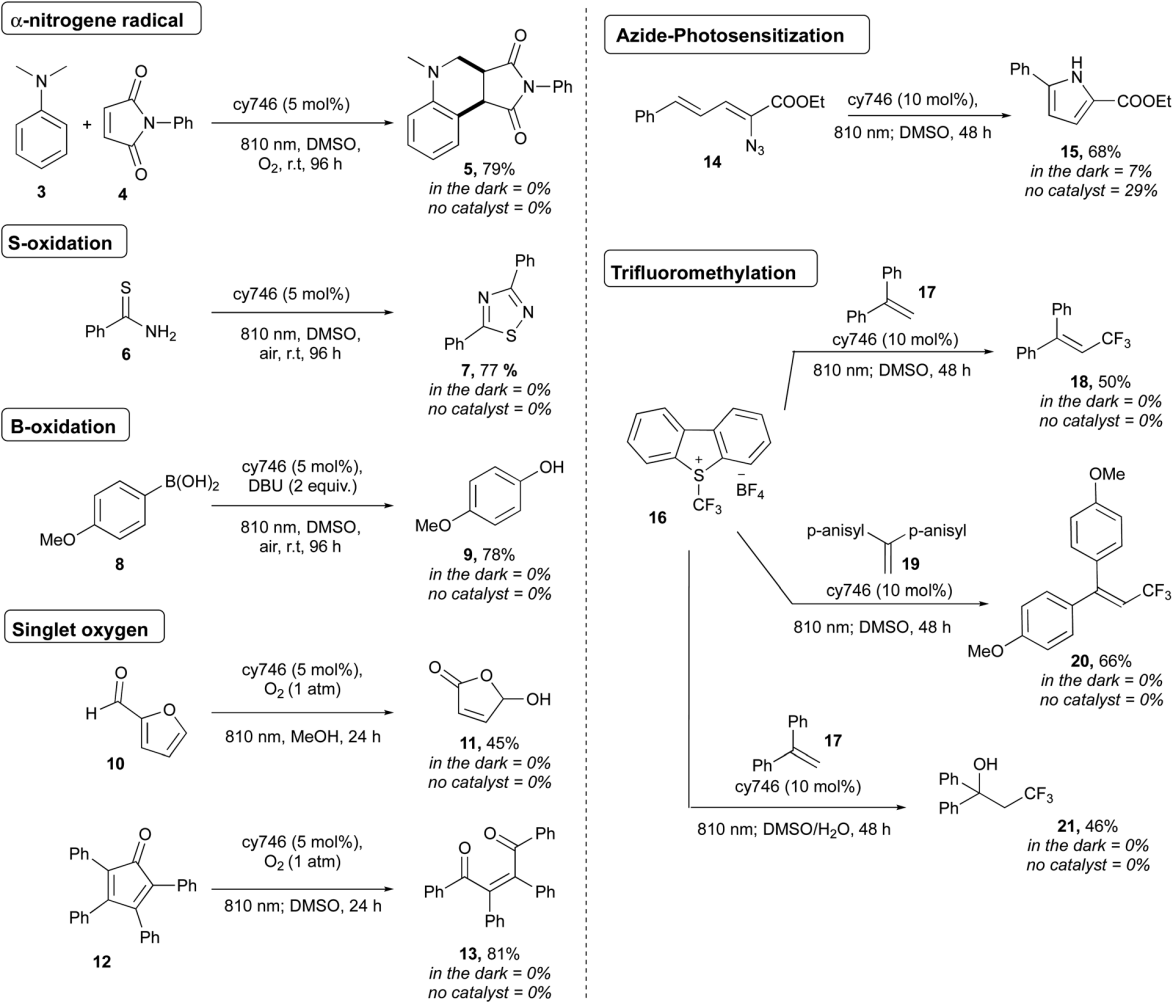
Selected examples of NIR-triggered reactions.

To demonstrate the synthetic potential of cyanines as photoredox catalysts, we extended the scope to heteroatom oxidations, photosensitization and onium salt reduction (Scheme 4). Thus, oxidation of *N*,*N*-dimethylaniline **3** (*E*_ox_ = 0.80 V *vs.* SCE)^17^ afforded the corresponding α-aminoalkyl radical which reacted with maleimide followed through a formal [4 + 2] cycloaddition and gave **cis-5** in 79% yield as a unique diastereoisomer.^18^ Thioamide **6** dimerized according to sulfur atom oxidation to provide 1,2,4-thiadiazole **7** in 77%.^19^ The oxidation of boronic acid **8** was done to generate **9** in 78% yield, as a mild alternative to classical H_2_O_2_ oxidation conditions.^20^

Cyanines are known to generate ^1^O_2_ for dynamic phototherapy.^21^ Thus, we took advantage of this to apply NIR O_2_ photosensitization to the oxidation of furfural **10** and tetraphenylcyclopentadienone **12** at 810 nm under O_2_ atmosphere to get respectively **11** and **13** in 45% and 74% yields.^22^ The photosensitization of vinyl azide was also successfully applied to **14**, producing, after cyclization, the disubstituted pyrrole **15** in 68% yield.^23^ Interestingly, cy746 is also capable to catalyze reductive processes. The Umemoto's reagent **16** (*E*_red_ = −0.75 V *vs.* Fc/Fc^+^) was reduced by cy746 under anaerobic conditions to performed trifluoromethylation reactions through the formation of the trifluoromethyl radical intermediate.^24^,^25^ Thus, this reactive radical added onto **17** to generate the resulting tertiary bis-benzylic radical that could be further oxidized to provide **18** (50%) in the absence nucleophile. This reaction was improved by using the more electron-rich olefin **19** to form **20** (66%). The compatibility of our NIR-photoredox catalysis with water media has been demonstrated by the formation of the hydroxy trifluoromethylation adduct **21** (46%), coming from the [CF_3_ radical addition/oxidation/water addition] sequence. It is important to note that no background reaction was observed without light and cy746 (Scheme 4). Further mechanistic considerations as well as two proposed mechanisms are reported in the ESI† section.

## Conclusions

In conclusion, we developed an original metal-free NIR-photoredox catalytic system as one of the first to promote organic transformations. This user-friendly method, based on commercial and stable cyanine (cy746), demonstrated its versatility for organic transformations (aza-Henry, heteroatom oxidation, photosensitization and reduction) under mild irradiation (810 nm). Additionally, we compared some photophysical properties with conventional photoredox catalysts. The design and synthesis of new cyanine-based photoredox catalysts are currently in progress in order to improve the reaction parameters.

## Author contributions

AROK, NS, AB, MC and JPG planned, ran and analyzed the experiments. MC and JPG designed and directed the project and wrote the manuscript with the help of AROK, NS and AB. All the authors contributed to the discussions.

## Conflicts of interest

There are no conflicts to declare.

## Supplementary Material

SC-012-D1SC00998B-s001
