# Supplementary material for: Cyanine-based near infra-red organic photoredox catalysis
Source: Chem Sci. 2021 Apr 13;12(20):6964–8. doi: 10.1039/d1sc00998b (PMC8153078; doi:10.1039/d1sc00998b)

Electronic Supplementary Material (ESI) for Chemical Science.
This journal is © The Royal Society of Chemistry 2021

Supporting Information

Cyanine-Based Near Infra-Red Organic Photoredox Catalysis

Anne Roly Obah Kosso, Nicolas Sellet, Alexandre Baralle,

Morgan Cormier* and Jean-Philippe Goddard*

Université de Haute-Alsace (UHA), Université de Strasbourg, CNRS, Laboratoire d'Innovation Moléculaire et Applications (LIMA) UMR 7042, 68100 Mulhouse, France

Contents

1. General methods 2
2. Properties of NIR-Photocatalysts 3
3. Synthesis of starting materials 7
4. NIR-Photoredox catalysis 9
5. Cy746 stability 17
6. Aza-Henry reaction: Kinetic experiments 19
7. Stern-Volmer experiments 22
8. Proposition of two mechanistic pathways 27
9. References 30
10. 1H and 13C NMR Spectra of Products 31

1. General methods

Unless otherwise stated, all the reagents were commercially available and used without further purification. A newly opened commercial grade dry DMSO and dry DMF were used directly. Other solvents (CH_3CN, DCM, MeOH, THF, CH_3NO_2) were distillated and stored under N_2 in absence of light. Thin Layer Chromatography (TLC) was performed using Merck© silica gel 60 F254 Aluminum sheets. Column chromatography was performed using Merk© Geduran© Si 60A silica gel (0.040-0.063mm) or Fluka© neutral Aliminum oxide (CAS = 1344-28-1). Cyanines photocatalysts (Indocyanine Green, IR-813, DTTCI, cy746) were purchased from TCI.
The absorption and emission spectra were recorded using a Molecular Devices SpectraMax ID3 UV-Visible multimode microplate reader.
IR spectra were recorded on a Perkin, Elmer Spectrum Two spectrometer equipped with a detector type (DTGS t) with a resolution of 0.5 cm^{-1}.
Liquid state ^{1}H and ^{13}C NMR spectra were recorded at 400.16 and 100.62 MHz respectively or 500 MHz and 126 MHz on a Bruker 400 spectrometer or a Bruker 500 spectrometer, respectively. All spectra were reported in δ (ppm) relative to TMS, with $CDCl_3$ as solvent. ^{1}H NMR splitting patterns are designated as singlet (s), doublet (d), triplet (t), quadruplet (q), dd (doublet of doublets), or m (multiplets).All the photocatalytic reactions were conducted using the folling NIR-LED from Thorlabs :

- M780LP1, light-emitting diode (LED, nominal wavelength: 780 nm, output power: 950 mW, irradiance: 13.3 μW/mm^2).
- M810L4 light-emitting diode (LED, nominal wavelength: 810 nm, output power: 542 mW, irradiance: 23.7 μW/mm^2).
- M940 L3 light-emitting diode (LED, nominal wavelength: 940 nm, output power: 1000 mW, irradiance: 19.1 μW/mm^2).

For some reactions, a Thorlabs adjustable collimation optic for IR was used (Ref: SM2F32-B).

Emission spectra of NIR-LED[1]

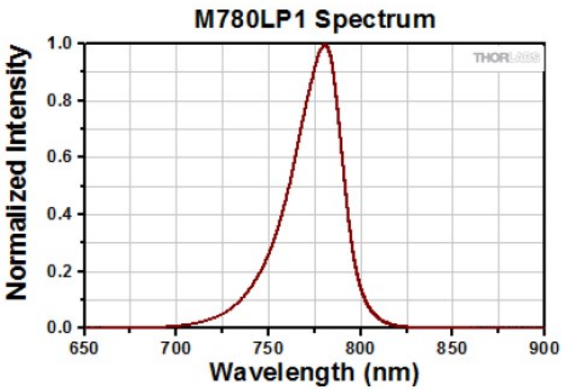

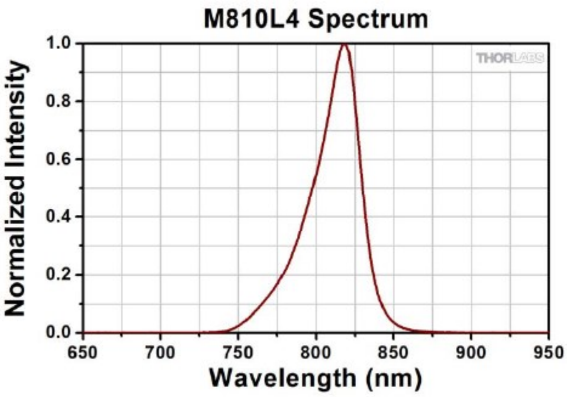

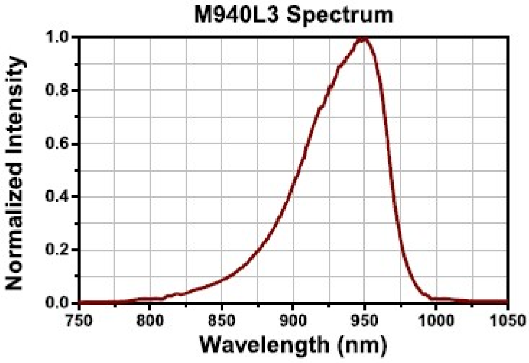

2. Properties of NIR-Photocatalysts

2.1. Redox Data for NIR-Photocatalysts

Structure	***Redox potentials***	***references***
IR-813	$E_{ox}[PC/PC^{+\cdot}]$ = 0.65 V vs Ag/AgCl	*Z. Phys. Chem.* **2014**, *228*,129–153
Indocyanine Green	$E_{ox}[PC/PC^{+\cdot}]$ = 0.56 V vs SHE<br>$E_{red}[PC/PC^{-\cdot}]$ = -0.59 V vs SHE	*J. Phys. Org. Chem.* **2010**, *23*, 893–903
DTCCI	$E_{red}[PC/PC^{-\cdot}]$ = -0.78 V vs SCE	*Helv. Chim. Acta* **2001**, *84*, 2796–2812

2.2. Procedure for recording Absorption and Fluorescence spectra

Absorption and fluorescence spectra of NIR-photocatalysts (ICG, DTTCI, IR-813, cy746) were recorded in DMSO (at 20 µM) using a SpectraMax ID3 spectrometer at ambient temperature (25 ◦C).

2.3. Indocyanine Green (ICG)

Chemical Formula: $C_{43}H_{47}N_2NaO_6S_2$ **Molecular Weight**: 774.97 g/mol **CAS**: 35599-32-4

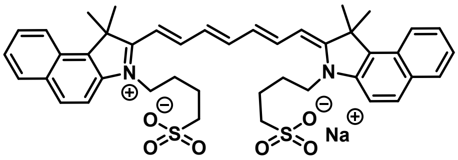

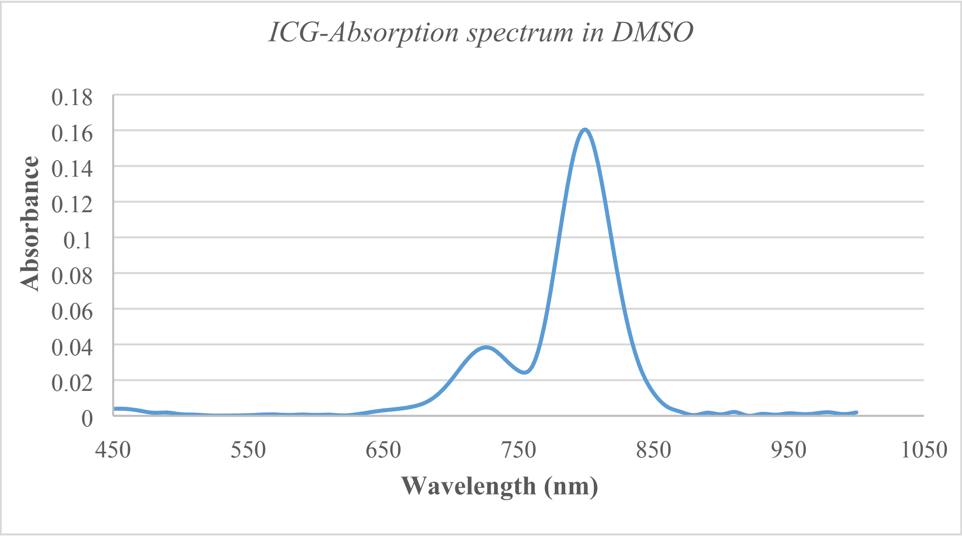

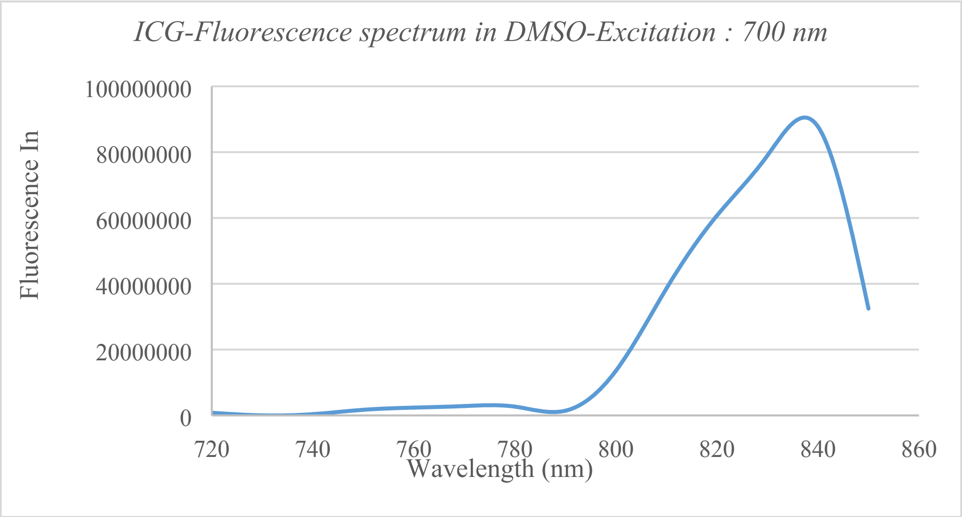

2.4. 3,3'-Diethylthiatricarbocyanine iodide (DTTCI)

Chemical Formula: $C_{25}H_{25}N_2S_2I$ **Molecular Weight**: 544.51 g/mol **CAS**: 3071-70-3

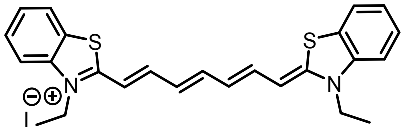

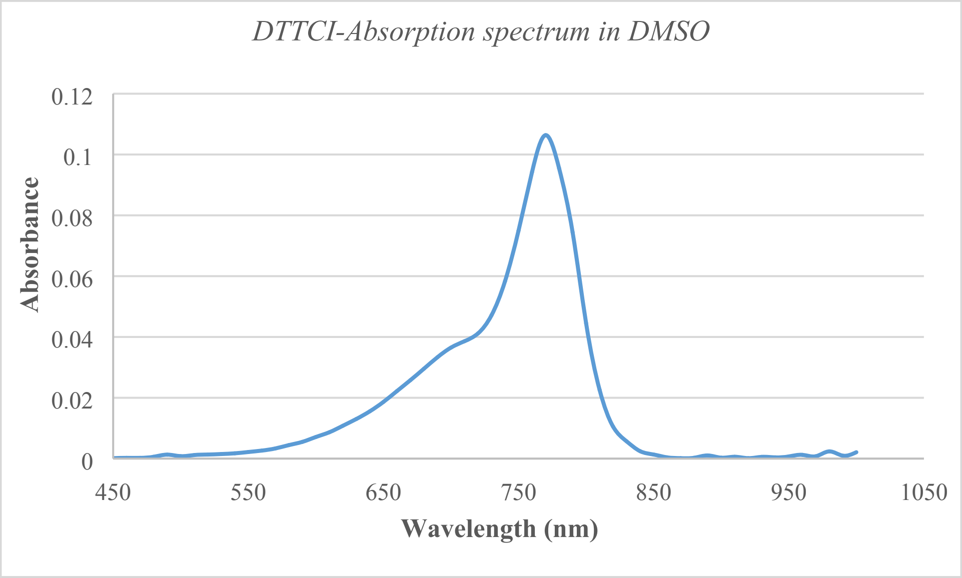

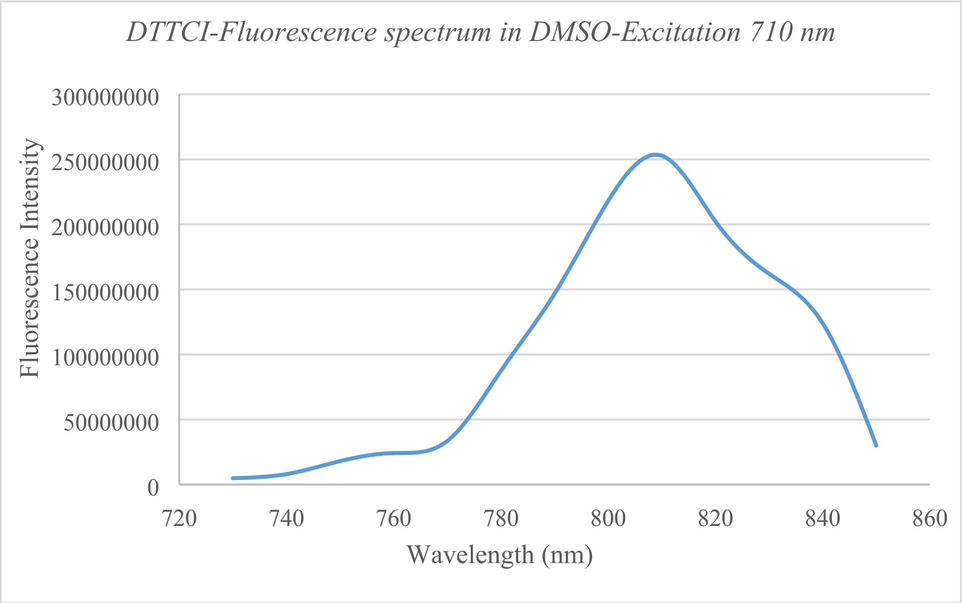
DTTCI-Fluorescence spectrum in DMSO-Excitation 710 nm
Fluorescence Intensity
300000000
250000000
200000000
150000000
100000000
50000000
0
720
740
760
780
800
820
840
860
Wavelength (nm)

2.5. IR-813 p-Toluenesulfonate (IR-813)

Chemical Formula: $C_{47}H_{47}ClN_2O_3S$ **Molecular Weight**: 755.41 g/mol **CAS**: 134127-48-3

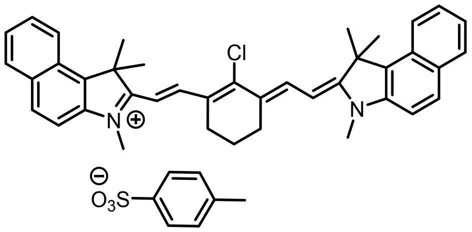

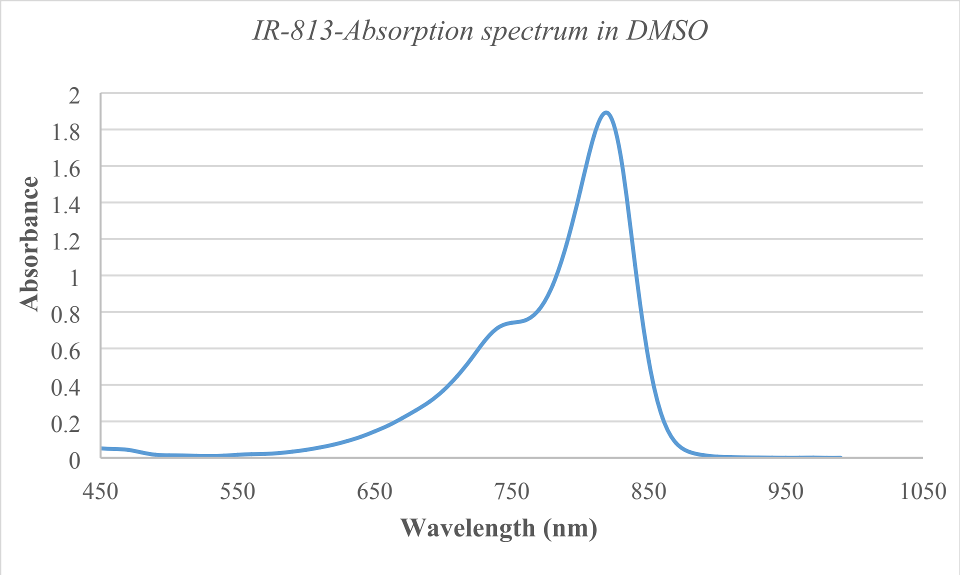

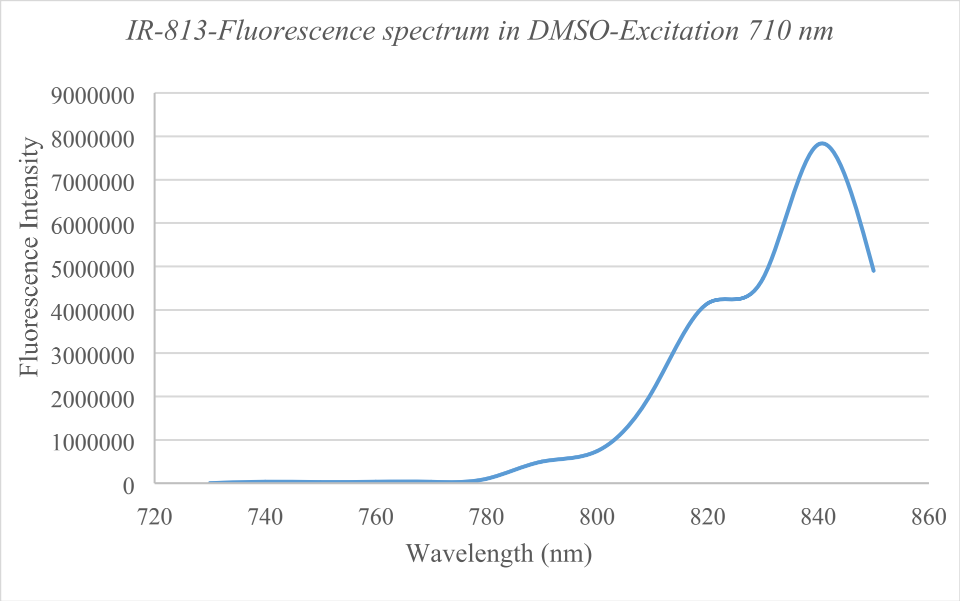

2.6. 1,1'-Dibutyl-3,3,3',3'-tetramethylindotricarbocyanine Hexafluorophosphate (IR-746)

Chemical Formula: $C_{35}H_{45}F_6N_2P$ **Molecular Weight**: 638.72 g/mol **CAS**: 134339-08-5

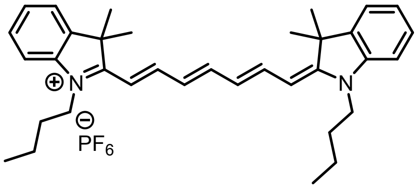

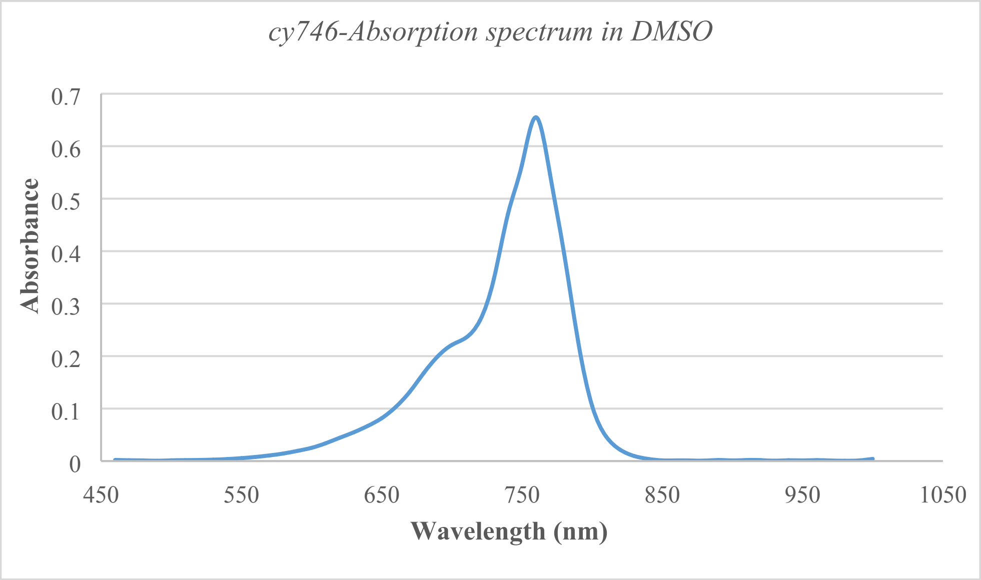

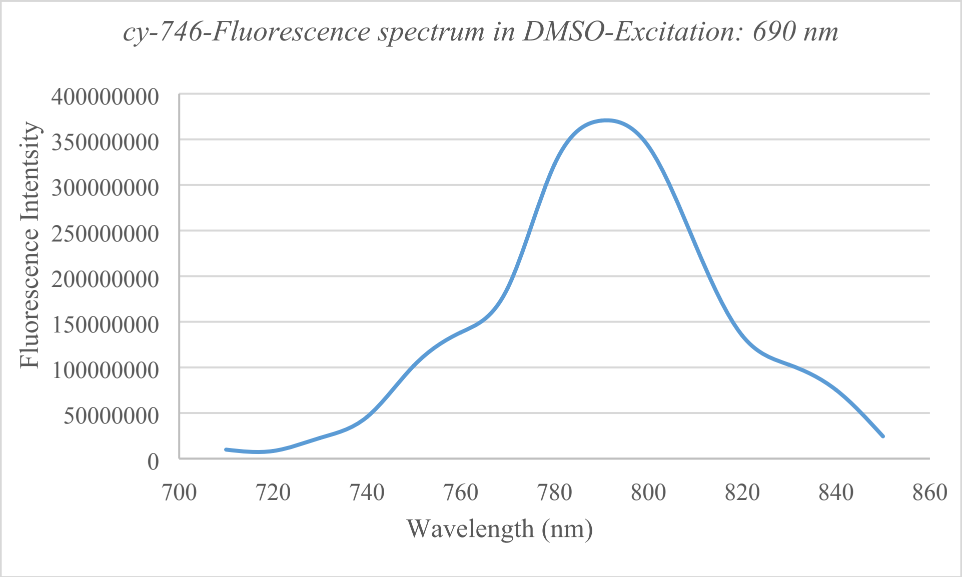

3. Synthesis of starting materials

3.1. Synthesis of N-aryl-tetrahydroisoquinoline derivatives

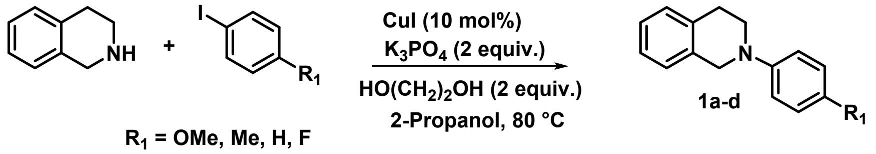

General procedure : The substrates **1a-d** were synthetized according to established procedure:[2] Copper(I) iodide (200 mg, 1.05 mmol, 10 mol%) and potassium phosphate (4.25 g, 20.0 mmol, 2 equiv.) were put into a Schlenk tube. The tube was evacuated and back filled with nitrogen. 2-Propanol (10.0 mL), ethylene glycol (1.11 mL, 20.0 mmol, 2 equiv.), 1,2,3,4-tetrahydro-isoquinoline (2.0 mL, 15 mmol, 1.5 equiv.) and iodoarene (10.0 mmol, 1 equiv.) were added successively at room temperature. The reaction mixture was heated at 85-90 °C and kept for 24 h and then allowed to cool to room temperature. Diethyl ether (20 mL) and water (20 mL) were then added to the reaction mixture. The organic layer was extracted by diethyl ether (2 × 20 mL). The combined organic phases were washed with brine and dried over magnesium sulfate. The solvent was removed by rotary evaporation and purified by column chromatography on silica gel (petroleum ether; petroleum ether/ethyl acetate = 95/5) to give the pure products **1a-d**.

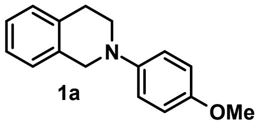

1a

2-(4-Methoxyphenyl)-1,2,3,4-tetrahydroisoquinoline (1a): Following the general procedure, **1a** is obtained as a white solid (m = 2 g, yield = 56%). Data are conformed to the literature.[2] **R_f** = 0.35 (petroleum ether/ethyl acetate = 95/5). **^{1}H NMR** (400 MHz, $CDCl_3$) δ 7.19 – 7.13 (m, 4H), 6.99 (d, *J* = 9.0 Hz, 2H), 6.87 (d, *J* = 9.0 Hz, 2H), 4.31 (s, 2H), 3.79 (s, 3H), 3.45 (t, *J* = 5.8 Hz, 2H), 2.99 (t, *J* = 5.7 Hz, 2H). **^{13}C NMR** (100 MHz, $CDCl_3$) δ 153.8, 144.9, 134.8, 131.7, 128.8, 126.7, 126.4, 126.0, 118.2 (2C), 114.7 (2C), 55.8, 52.8, 48.6, 29.3.

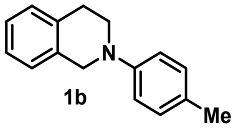

1b

2-(*p*-tolyl)-1,2,3,4-Tetrahydroisoquinoline (1b): Following the general procedure, **1b** is obtained as a light yellow oil (m = 1.38 g, yield = 41%). Data are conformed to the literature.[3] **R_f** = 0.37 (petroleum ether/ethyl acetate = 95/5). **^{1}H NMR** (400 MHz, $CDCl_3$) δ 7.21 – 7.15 (m, 4H), 7.12 (d, *J* = 8.2 Hz, 2H), 6.94 (d, *J* = 8.6 Hz, 2H), 4.38 (s, 2H), 3.53 (t, *J* = 5.9 Hz, 2H), 3.00 (t, *J* = 5.8 Hz, 2H), 2.30 (s, 3H). **^{13}C NMR** (100 MHz, $CDCl_3$) δ 148.8, 134.9, 134.7, 129.8 (2C), 128.7, 128.5, 126.7, 126.4, 126.1, 116.0 (2C), 51.6, 47.4, 29.2, 20.5.

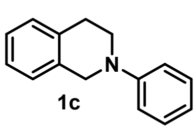

1c

2-phenyl-1,2,3,4-Tetrahydroisoquinoline (1c): Following the general procedure, **1c** is obtained as a light yellow oil (m = 2.1 g, yield = 63%). Data are conformed to the literature.[2] **R_f** = 0.45 (petroleum ether/ethyl acetate = 95/5). **^{1}H NMR** (400 MHz, $CDCl_3$) δ 7.32 – 7.26 (m, 2H), 7.20 – 7.16 (m, 4H), 7.00 (dd, *J* = 8.8, 0.9 Hz, 2H), 6.84 (tt, *J* = 7.3, 1.0 Hz, 1H), 4.42 (s, 2H), 3.58 (t, *J* = 5.9 Hz, 2H), 3.00 (t, *J* = 5.8 Hz, 2H). **^{13}C NMR** (100 MHz, $CDCl_3$) δ 150.7, 135.0, 134.6, 129.3 (2C), 128.6, 126.7, 126.4, 126.1, 118.8, 115.3 (2C), 50.9, 46.6, 29.3.

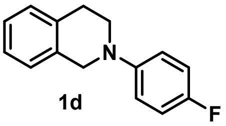

1d

2-(4-fluorophenyl)-1,2,3,4-tetrahydroisoquinoline (1d): Following the general procedure, **1d** is obtained as a white solid (m = 1.27g, yield = 37%). Data are conformed to the literature.[3] **R_f** = 0.50 (petroleum ether/ethyl acetate = 95/5). **^{1}H NMR** (400 MHz, $CDCl_3$) δ 7.21 – 7.01 (m, 4H), 7.00 – 6.91 (m, 4H), 4.34 (s, 2H), 3.50 (t, *J* = 5.9 Hz, 2H), 3.00 (t, *J* = 5.8 Hz, 2H). **^{13}C NMR** (100 MHz, $CDCl_3$) δ 156.9 (d, *J* = 238 Hz), 147.5 (d, *J* = 1.8 Hz), 134.7, 134.4, 128.8, 126.5, 126.2,117.3, 117.2, 115.8, 115.6, 52.0, 47.9, 29.2.

3.2. Synthesis of ethyl (2E,4E)-2-methyl-5-phenylpenta-2,4-diene 24

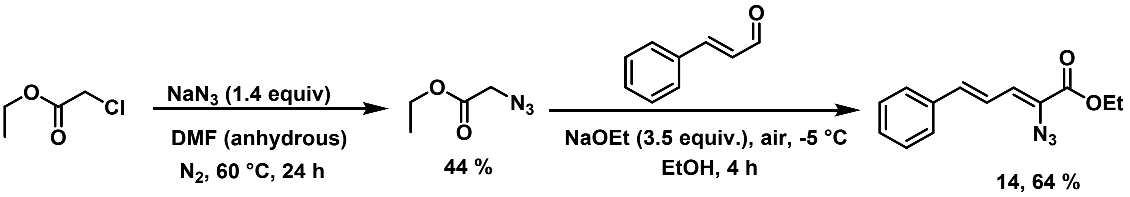

Ethyl 2-chloroacetate (6.7 g, 55,2 mmol, 1 equiv.), sodium azide (5.1 g, 78 mmol, 1.4 equiv.) and 60 mL of anhydrous DMF were charged in a round bottom flask with a magnetic stirring bar. The mixture was heated at 60 °C for 24 h. After the reaction, the mixture was cooled down to r.t. and 100 mL of H_2O was added. After the reaction, H_2O was added. The crude product was extracted with AcOEt, the combined organic phases were dried ($MgSO_4$), and filtered. The filtrate was concentrated *in vacuo* to afford **ethyl 2-azidoacetate** (m = 3.1 g, crude yield = 44%) as a colorless oil which was used for the next step without any further purification. Data are conformed to the literature.[4] **R_f** = 0.55 (1/9 : EtOAc/PE). **^{1}H NMR** (400 MHz, $CDCl_3$) δ 4.26 (q, *J*= 14.3, 7.2 Hz, 2H), 3.86 (s, 2H), 1.31 (t, *J*= 7.2 Hz, 3H).**^{13}C NMR** (126 MHz, $CDCl_3$) δ 168.4, 62.0, 50.5, 14.3. **FT-IR (ν/cm^{-1}, neat):** 2103, 1740, 1191.

To a cooled solution (-5 °C) of trans-cynnamaldehyde (2.21 mmol, 1 equiv.) and ethyl 2-azidoacetate (1 g, 7.74 mmol, 3.5 equiv.) in EtOH (4 mL) was added dropwise with a syringe pump a solution of NaOEt (7.7 mmol, 3.5 equiv.) in EtOH (1.5 mL) over 30 min. The reaction was stirred at -5 °C for 2 h. Then the solution was diluted with 10 mL of H_2O, extracted with DCM (2 x 20 mL) and washed with H_2O (2 x 20 mL) and brine (20 mL). Organic phase was dried (Na_2SO_4) and filtered. The filtrate was concentrated *in vacuo* to afford **14** (m = 344 mg, crude yield = 64 %) as a light yellow solid which was used for the next step without any further purification. Data are conformed to the literature.[5] **R_f** = 0.65 (1/9 : EtOAc/PE). **^{1}H NMR** (400 MHz, $CDCl_3$) δ 7.50-7.48 (m, 2H), 7.37-7.34 (m, 2H), 7.31-7.28 (m, 1H), 7.20-7.14 (m, 1H), 6.82-6.74 (m, 2H), 4.33 (q, *J*= 14.3, 7.2 Hz, 2H), 1.38 (t, *J*= 7.2 Hz, 3H). **^{13}C NMR** (126 MHz, $CDCl_3$) δ 163.3, 139.1, 136.5, 129.1, 128.9 (2C), 127.4 (2C), 127, 125.9, 122.4, 62.1, 14.4. **FT-IR (ν/cm^{-1}, neat):** 2102, 1699, 1232.

3.3. Synthesis of 1,1-bis(4-methoxyphenyl)ethene 19

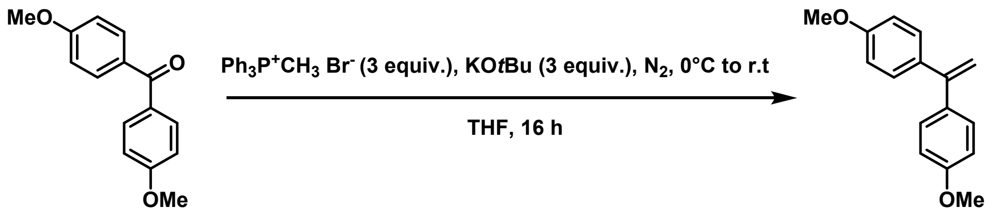

19, 99 %

Methyltriphenylphosphonium bromide (2.1 g, 6 mmol, 3 equiv.) was suspended in dry THF (10 mL, 0.2 M) at 0 °C. KO*t*Bu (672 mg, 6 mmol, 3 equiv.) was added to the suspension and a bright yellow color was observed. The mixture was stirred at 0 °C for 30 min. Then 4, 4'-Dimethoxybenzophenone (484 mg, 2 mmol, 1 equiv.) was added and the reaction mixture was stirred at 0 °C to rt for 16 h. The reaction was quenched with H_2O (10 mL) and extracted with diethyl ether (4 x 6 mL). The combined organic layers were dried over Na_2SO_4, filtered, and concentrated *in vacuo* to give crude alkene **19**. The crude was purified by column chromatography on silica gel (EtOAc: cyclohexane = 2:8) to afford 1,1-bis(4-methoxyphenyl)ethene **19** (m = 475 mg, yield = 99%) as a white solid. Data are conformed to the literature.[6] $\mathbf{R_f}$ = 0.83 (EtOAc:Cyclohexane = 2:8). **^{1}H NMR** (400 MHz, $CDCl_3$) δ 7.27 (m, 4H), 6.87 (m, 4H), 5.29 (s, 2H), 3.83 (s, 6H). **^{13}C NMR** (126 MHz, $CDCl_3$) δ 159.5 (2C), 149.1, 134.5 (2C), 129.6 (4C), 113.6 (4C), 111.8, 55.4 (2C). **FT-IR (ν/cm^{-1}, neat):** 2102, 1699, 1232.

4. NIR-Photoredox catalysis

4.1. Set-up

The reactions were run in 10 mL round bottom single neck flask or in a 5 mL glass tube equipped with magnetic stir bar and closed by a septum. One near infrared LED (810 nm) is placed at 3 cm away from the light source. The system is fully covered by aluminum foil to remove the external visible light . Reaction was performed at room temperature.

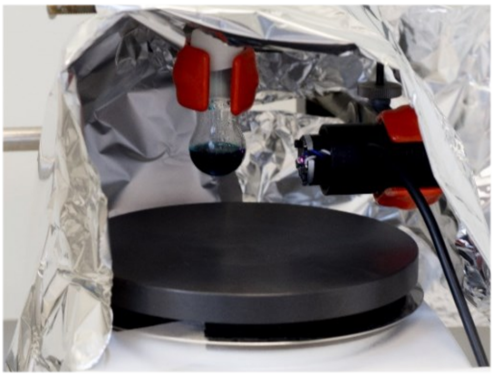

4.2. Optimization Table of Aza-Henry

Reactions were run in 0.13 mmol scale, C = 0.1 M and x mol% of Photocatalyst (PC). ^{1}H-NMR conversions determined on the crude reaction mixture.

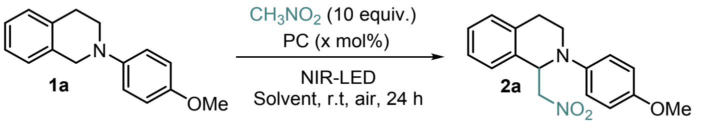

Entry	PC	x(mol%)	solvent	λ (nm)	Conv.(%)
1	ICG	10	CH_3NO_2	810	16
2	IR-813	10	CH_3NO_2	810	28
3	DTCCI	10	CH_3NO_2	810	14
4	cy746	10	CH_3NO_2	810	57
5	cy746	10	CH_3NO_2	780	14

6	cy746	10	CH_3NO_2	940	<5
7	cy746	10	DCM	810	48
8	cy746	10	DMF	810	n.r
9	cy746	10	MeOH	810	28
10	cy746	10	DMSO	810	91
11	cy746	5	DMSO	810	85
12	cy746	1	DMSO	810	67
13	cy746	-	DMSO	810	16
14	cy746	5	DMSO	dark	n.r

4.3. Aza-henry with nitroalkanes

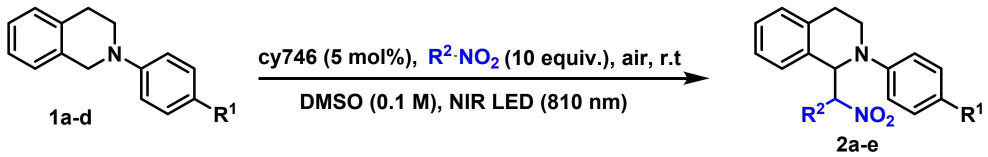

Entry	R^1	R^2	Time	Yields
1	OMe	H	24 h	87%
2	Me	H	24 h	79%
3	H	H	48 h	70%
4	F	H	48 h	82%
5	H	Et	24 h	71%

General Procedure: 2-Aryl-1,2,3,4-tetrahydroisoquinoline (0.13 mmol, 1 equiv.), nitroalkane (1.3 mmol, 10 equiv.), cy746 (4 mg, 0.0065 mmol, 5 mol%) and DMSO (1.3 mL) were charged in a reaction glass tube with magnetic stirring bar. The reaction mixture was stirred under air atmosphere and near infrared LED (810 nm) irradiation (3 cm away from the light source) at room temperature for 24 to 48 h. The crude mixture was extracted with ethyl acetate (3 x 10 mL). The combined organic layers were dried over Na_2SO_4, filtered and concentrated under vacuum pressure. The crude was purified by column chromatography on aluminum oxide (EtOAc: cyclohexane = 10:90, R_f = 0.25, 0.29, 0.32, 0.38, 0.41) to give **2a-e**.

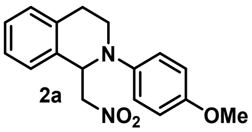

2-(4-methoxyphenyl)-1-(nitromethyl)-1,2,3,4-tetrahydroisoquinoline (2a): According to the general procedure, **2a** is obtained after 24 h of reaction as yellow oil (m = 34 mg, yield = 87%). Data are conformed to the literature.[3] **R_f** = 0.25 (EtOAc: cyclohexane = 10:90). **^{1}H NMR** (400 MHz, $CDCl_3$) δ 7.25 – 7.13 (m, 4H), 6.92 (dt, *J* = 9.0, 2.5 Hz, 2H), 6.84 (dt, *J* = 9.1, 2.6 Hz, 2H), 5.39 (dd, *J* = 8.5, 5.9 Hz, 1H), 4.83 (dd, *J* = 11.9, 8.5 Hz, 1H), 4.56 (dd, *J* = 11.9, 5.9 Hz, 1H), 3.76 (s, 3H), 3.58 - 3.56 (m, 2H), 3.00 (ddd, *J* = 16.2, 9.1, 6.8 Hz, 1H), 2.69 (dt, *J* = 16.5, 3.9 Hz, 1H). **^{13}C NMR** (100 MHz, $CDCl_3$) δ 154.1, 143.2, 135.6,

133.0, 129.6, 128.0, 127.0, 126.8, 119.0 (2C), 114.8 (2C), 79.1, 59.0, 55.7, 43.3, 25.9. **FT-IR (ν/cm^{-1}, neat):** 3675, 2970, 2901, 1550, 1509, 1379, 1244, 1037.

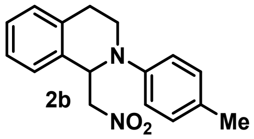

1-(nitromethyl)-2-(*p*-tolyl)-1,2,3,4-tetrahydroisoquinoline (2b): According to the general procedure, **2b** is obtained after 24 h of reaction as yellow oil (m = 29 mg, yield = 79%). Data are conformed to the literature.[3] **R$_f$** = 0.29 (EtOAc: cyclohexane = 10:90). **^{1}H NMR** (400 MHz, $CDCl_3$) δ 7.27 – 7.13 (m, 4H), 7.08 (d, *J* = 8.3 Hz, 2H), 6.90 (dt, *J* = 8.6, 2.3 Hz, 2H), 5.50 (t, *J* = 7.2, 1H), 4.85 (dd, *J* = 11.8, 8.1 Hz, 1H), 4.56 (dd, *J* = 11.8, 6.4 Hz, 1H), 3.63 – 3.59 (m, 2H), 3.07 – 3.05 (m, 1H), 2.78 (dt, *J* = 16.4, 4.5 Hz, 1H), 2.27 (s, 3H). **^{13}C NMR** (100 MHz, $CDCl_3$) δ 146.5, 135.5, 133.1, 130.1 (2C), 129.4, 129.2, 128.1, 127.1, 126.7, 116.0 (2C), 79.0, 58.5, 42.4, 26.4, 20.5. **FT-IR (ν/cm^{-1}, neat):** 3675, 2973, 2900, 1553, 1514, 1265, 1048, 733.

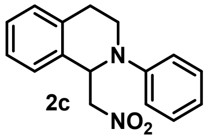

1-(nitromethyl)-2-phenyl-1,2,3,4-tetrahydroisoquinoline (2c): According to the general procedure, **2c** is obtained after 48 h of reaction as yellow oil (m = 24 mg, yield = 70%). Data are conformed to the literature.[3] **R$_f$** = 0.32 (EtOAc: cyclohexane = 10:90). **^{1}H NMR** (400 MHz, $CDCl_3$) 7.31 – 7.13 (m, 6H), 6.99 (d, *J* = 8.4 Hz, 2H), 6.86 (t, *J* = 7.2 Hz, 1H), 5.56 (t, *J* = 7.3 Hz, 1H), 4.88 (dd, *J* = 11.8, 7.8 Hz, 1H), 4.57 (dd, *J* = 11.8, 6.6 Hz, 1H), 3.68 – 3.62 (m, 2H), 3.14 – 3.06 (m, 1H), 2.80 (dt, *J* = 16.3, 4.9 Hz, 1H). **^{13}C NMR** (100 MHz, $CDCl_3$) δ 148.5, 135.4, 133.0, 129.6 (2C), 129.3, 128.2, 127.1, 126.8, 119.5, 115.2 (2C), 78.9, 58.3, 42.2, 26.6. **FT-IR (ν/cm^{-1}, neat):** 3670, 2986, 2923, 1552, 1264, 1086, 737.

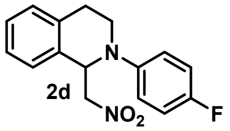

2-(4-fluorophenyl)-1-(nitromethyl)-1,2,3,4-tetrahydroisoquinoline (2d): According to the general procedure, **2d** is obtained after 48 h of reaction as yellow oil (m = 30 mg, yield = 82%). Data are conformed to the literature.[3] **R$_f$** = 0.32 (EtOAc: cyclohexane = 10:90). **^{1}H NMR** (400 MHz, $CDCl_3$) δ 7.28 – 7.14 (m, 4H), 6.97 – 6.88 (m, 4H), 5.43 (dd, *J* = 8.6, 5.9 Hz, 1H), 4.84 (dd, *J* = 12.0, 8.7 Hz, 1H), 4.58 (dd, *J* = 12.0, 5.9 Hz, 1H), 3.60 (dd, *J* = 9.0, 4.3 Hz, 2H), 3.07 – 2.99 (m, 1H), 2.73 (dt, *J* = 16.5, 4.2 Hz, 1H). **^{13}C NMR** (100 MHz, $CDCl_3$) δ 157.3 (d, *J* = 239.2 Hz), 145.4 (d, *J* = 2.2 Hz), 135.4, 132.7, 129.6, 128.2, 127.1, 126.9, 118.1 (d, *J* = 7.7 Hz, 2C), 116.0 (d, *J* = 22.2 Hz, 2C), 79.0, 58.8, 42.9, 25.9. **^{19}F NMR** (376 MHz, $CDCl_3$) δ -124.3. **FT-IR (ν/cm^{-1}, neat):** 3005, 2928, 1555, 1508, 1264, 737.

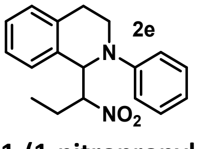

1-(1-nitropropyl)-2-phenyl-1,2,3,4-tetrahydroisoquinoline (2e): According to the general procedure, **2e** is obtained after 24 h of reaction as yellow oil (m = 27 mg, yield = 71%). Data are conformed to the literature.[7] **R$_f$** = 0.41 (EtOAc: cyclohexane = 10:90). **^{1}H NMR** (500 MHz, $CDCl_3$) diastereomeric ratio (1:0.6) was determined from the crude reaction δ 7.31 – 7.15 (m, 11H, mixture of isomers), 7.01 – 6.94 (m, 5H, mixture of isomers), 6.84 – 6.78 (m, 2H, mixture of isomers), 5.25 (d, *J* = 9.3 Hz, 1H, minor

isomer), 5.14 (d, J = 9.6 Hz, 1H, major isomer), 4.87 (ddd, J = 3.0, 9.6, 11.9 Hz, 1H, major isomer), 4.68 (ddd, J = 3.1, 9.3, 11,8 Hz, 1H, minor isomer), 3.86 – 3.82 (m, 1H, major isomer), 3.69 – 3.59 (m, 2H, mixture of isomers), 3.56 – 3.53 (m, 1H, minor isomer), 3.09 – 3.04 (m, 2H, mixture of isomers), 2.93 – 2.86 (m, 2H, mixture of isomers), 2.22 – 2.11 (m, 3H, mixture of isomers), 1.83 (dqd, J = 14.8, 7.5, 3.1 Hz, 1H, major isomer), 0.97-0.93 (m, 6H, mixture of isomers). **^{13}C NMR** (125 MHz, $CDCl_3$) For the mixture of diastereoisomers δ 149.2, 149.1, 135.7, 134.8, 134.0, 132.7, 129.6 (2C), 129.5, 129.3 (2C), 128.8, 128.7, 128.4, 128.3, 127.4, 126.8, 126.0, 119.5, 118.7, 116.0 (2C), 114.2 (2C), 96.3, 93.2, 62.3, 60.8, 43.7, 42.4, 27.0, 25.9, 25.1, 24.8, 10.8. **FT-IR (ν/cm^{-1}, neat):** 3675, 2973, 2901, 1552, 1393, 1265, 1050, 879, 737.

4.4. Synthesis of Dimethyl-2-(2-phenyl-1,2,3,4-tetrahydroisoquinolin-1-yl)malonate 2f

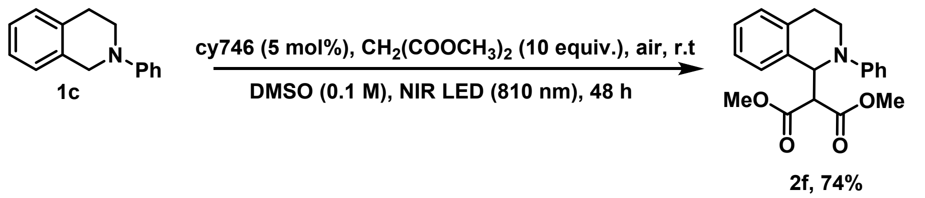

2-Phenyl-1,2,3,4-tetrahydroisoquinoline **1c** (27 mg, 0.13 mmol, 1 equiv.), dimethyl malonate (172 mg, 1.3 mmol, 10 equiv.), cy746 (4 mg, 0.0065mmol, 5 mol%) and DMSO (1.3 mL) were charged in a reaction glass tube with magnetic stirring bar. The reaction mixture was stirred under air atmosphere and near infrared LED (810 nm) irradiation (3 cm away from the light source) at room temperature for 48 h. The crude mixture was extracted with ethyl acetate (3 x 10 mL). The combined organic layers were dried over Na_2SO_4, filtered and concentrated under vacuum pressure. The crude was purified by column chromatography on aluminum oxide (EtOAc: cyclohexane = 10:90,) to give **2f** (m = 33 mg, yield = 74%) as a yellow oil. Data are conformed to the literature.[8] **R_f** = 0.30 (EtOAc: cyclohexane = 10:90,) **^{1}H NMR** (400 MHz, $CDCl_3$) δ 7.23-7.16 (m, 4H), 7.13-7.11 (m, 2H), 6.98 (d, J = 8.3 Hz, 2H), 6.76 (t, J = 7.2 Hz, 1H), 5.70 (d, J = 9.4 Hz, 1H), 3.95 (d, J = 9.4 Hz, 1H), 3.73-3.62 (m, 5H), 3.55 (s, 3H), 3.11-3.03 (m, 1H), 2.88 (dt, J = 16.5, 5.2 Hz, 1H). **^{13}C NMR** (100 MHz, $CDCl_3$) δ168.3, 167.4, 148.8, 135.7, 134.8, 129.1 (2C), 129.0, 127.6, 127.1, 126.0, 118.6, 115.2 (2C), 59.1, 58.2, 52.6, 52.5, 42.2, 26.1. **FT-IR (ν/cm^{-1}, neat):** 2973, 2896, 1735, 1586, 1265, 1049, 736.

4.5. Synthesis 2-(4-methoxyphenyl)-1,2,3,4-tetrahydroisoquinoline-1-carbonitrile 2g

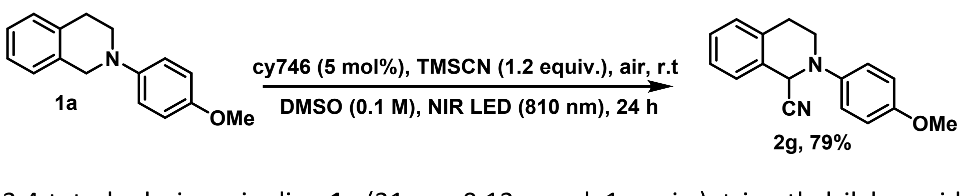

2-Aryl-1,2,3,4-tetrahydroisoquinoline **1a** (31 mg, 0.13 mmol, 1 equiv.), trimethylsilyl cyanide (TMSCN) (16 mg, 0.16 mmol, 1.2 equiv.), cy746 (4 mg, 0.0065 mmol, 5 mol%) and DMSO (1.3 mL) were charged in a reaction glass tube with magnetic stirring bar. The reaction mixture was stirred under air atmosphere and near infrared LED (810 nm) irradiation (3 cm away from the light source) at room temperature for 24 h. The crude mixture was extracted with ethyl acetate (3 x 10 mL). The combined organic layers were dried over Na_2SO_4, filtered and concentrated under vacuum pressure. The crude was purified by column chromatography on aluminum oxide (EtOAc: cyclohexane = 10:90,) to give **2g** (m = 27 mg, yield = 79%) as a yellow oil. Data are conformed to the literature.[10] **R_f** = 0.22 (EtOAc: cyclohexane = 10:90) **^{1}H NMR** (500 MHz, $CDCl_3$) δ 7.33-7.31 (m, 1H), 7.30-7.27 (m, 2H), 7.26-7.23 (m, 1H), 7.09 (d, J = 9.0 Hz, 2H), 6.92 (d, J = 9.0 Hz, 2H), 5.37 (s, 1H), 3.81 (s, 3H), 3.61-3.56 (m, 1H), 3.44

(td, J = 7.8, 4.17 Hz, 1H), 3.21-3.14 (m, 1H), 2.96-2.92 (m, 1H).**^{13}C NMR** (125 MHz, $CDCl_3$) δ 155.8, 142.7, 134.5, 129.8, 129.6, 128.8, 127.2, 126.8, 121.2 (2C), 117.7, 114.9 (2C), 55.8, 55.7, 45.0, 28.8. **FT-IR (ν/cm^{-1}, neat):** 2927, 2834, 1716, 1510, 1462, 1245, 1034, 936, 826.

4.6. Synthesis of diethyl (2-(4-methoxyphenyl)-1,2,3,4-tetrahydroisoquinolin-1-yl)phosphonate 2h

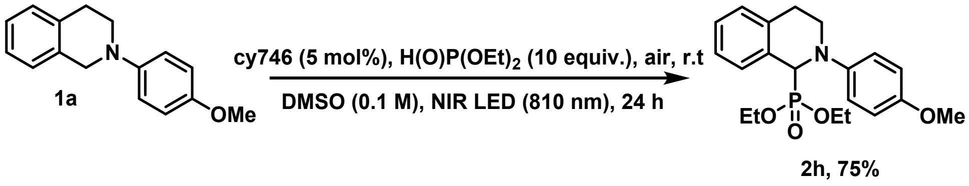

2-Aryl-1,2,3,4-tetrahydroisoquinoline **1a** (31 mg, 0.13 mmol, 1 equiv.), diethyl phosphite (180 mg, 1.3 mmol, 10 equiv.), cy746 (4 mg, 0.0065 mmol, 5 mol%) and DMSO (1.3 mL) were charged in a reaction glass tube with magnetic stirring bar. The reaction mixture was stirred under air atmosphere and near infrared LED (810 nm) irradiation (3 cm away from the light source) at room temperature for 24 h. The crude mixture was extracted with ethyl acetate (3 x 10 mL). The combined organic layers were dried over Na_2SO_4, filtered and concentrated under vacuum pressure. The crude was purified by column chromatography on aluminum oxide (EtOAc:Cyclohexane = 20:80) to give **2h** (m = 37 mg , yield = 75%). Data are conformed to the literature.[11] **R_f** = 0.2 (EtOAc:Cyclohexane = 20:80). **^{1}H NMR** (500 MHz, $CDCl_3$) δ 7.39-7.38 (m, 1H), 7.19-7.12 (m, 3H), 6.92 (d, J = 8.8 Hz, 2H), 6.81 (d, J = 8.8 Hz, 2H), 5.02 (d, J = 21.2 Hz, 1H), 4.11-3.9 m, 5H), 3.75 (s, 3H), 3.56-3.53 (m, 1H), 2.93-2.91 (m, 2H), 1.25 (t, J = 7.0 Hz, 3H), 1.15 (t, J = 7.0 Hz, 3H). **^{13}C NMR** (125 MHz, $CDCl_3$) δ 153.2, 144.3 (d, J = 8.4 Hz), 136.5 (d, J = 5.8 Hz), 130.6, 129.0 (d, J = 2.7 Hz), 128.3 (d, J = 4.7 Hz), 127.4 (d, J = 3.6 Hz), 125.9 (d, J = 2.8 Hz), 117.7 (2C), 114.6 (2C), 63.4 (d, J = 7.5 Hz), 62.3 (d, J = 7.6 Hz), 60.2, 59.0, 55.8, 44.8, 26.2, 16.6 (dd, J = 12.6, 5.7 Hz). **^{31}P NMR** (203 MHz, $CDCl_3$) δ 22.2. **FT-IR (ν/cm^{-1}, neat):** 3369, 2974, 1511, 1265, 1046, 971, 737.

4.7. Synthesis of 2-(4-methoxyphenyl)-1-(phenylethynyl)-1,2,3,4-tetrahydroisoquinoline 2i

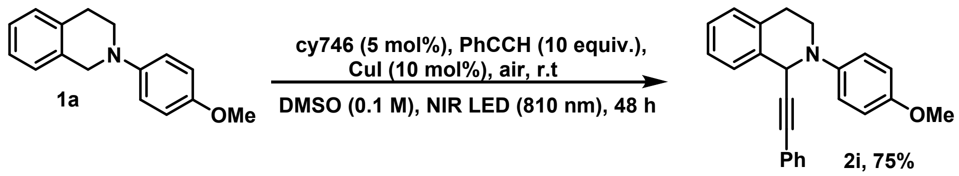

2-Aryl-1,2,3,4-tetrahydroisoquinoline **1a** (31 mg, 0.13 mmol, 1 equiv.), phenylethyne (27 mg, 0.26 mmol, 2 equiv.), cy746 (4 mg, 0.0065mmol, 5 mol%), CuI (2.5 mg, 0.013 mmol, 10 mol%) and DMSO (1.3 mL) were charged in a reaction glass tube with magnetic stirring bar. The reaction mixture was stirred under air atmosphere and near infrared LED (810 nm) irradiation (3 cm away from the light source) at room temperature for 48 h. The crude mixture was extracted with ethyl acetate (3 x 10 mL). The combined organic layers were dried over Na_2SO_4, filtered, and concentrated under vacuum pressure. The crude was purified by column chromatography on aluminum oxide (EtOAc: cyclohexane = 10:90,) to give **2i** (m = 33 mg, yield = 75%) as a yellow oil. Data are conformed to the literature.[9] **R_f** = 0.45 (EtOAc : cyclohexane = 10:90). **^{1}H NMR** (500 MHz, $CDCl_3$) δ 7.36-7.34 (m, 1H), 7.29-7.27 (m, 3H), 7.24-7.19 (m, 5H), 7.12 (d, J = 9.2 Hz, 2H), 6.89 (d, J = 9.1 Hz, 2H), 5.51 (s, 1H), 3.79 (s, 3H), 3.67-3.55 (m, 2H), 3.19-3.12 (m, 1H), 2.94 (dt, J = 10.3, 3,2 Hz, 1H).**^{13}C NMR** (125 MHz, $CDCl_3$) δ 154.4, 144.3,

135.6, 134.2, 131.8 (2C), 129.2, 128.2 (2C), 128.1, 127.6, 127.3, 126.2, 123.2, 120.3 (2C), 114.5 (2C), 88.6, 85.6, 55.7, 54.5, 44.3, 29.2. **FT-IR (ν/cm^{-1}, neat):** 3374, 2974, 1511, 1265, 1047, 879, 737

4.8. Synthesis of 5-Methyl-2-phenyl-3a,4,5,9b-tetrahydro-1H-pyrrolo[3,4-c]quinoline1,3(2H)-dione (5)

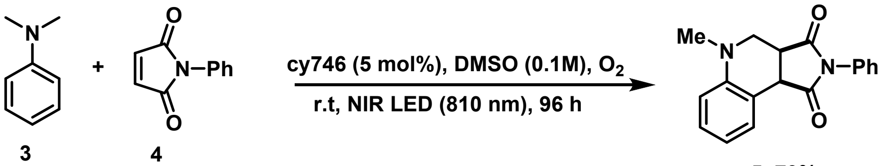

3 4 5, 79%

The *N,N*-dimethylanilines **3** (146 mg, 1.21 mmol, 7 equiv), *N*-phenyl maleimide **4** (29 mg, 0.17 mmol, 1 equiv), cy746 (5 mg, 0.0085 mmol, 5 mol%) and DMSO (1.7 mL) were placed in a standard glass reaction tube with magnetic stir bar. The reaction mixture was stirred under oxygen atmosphere (1 atm, balloon) and near infrared LED (810 nm), irradiation (3 cm away from the light source) at room temperature for 96 h. The crude mixture was extracted with ethyl acetate (3 x 10 mL). The combined organic layers were dried over Na_2SO_4, filtered and concentrated under vacuum pressure. The crude was purified by column chromatography on silica gel (EtOAc: cyclohexane = 20:90) to give **5** (m= 39 mg, yield = 79%) as a yellow oil and single diastereoisomer. Data are conformed to the litereature.[12] **R_f** = 0.2 (EtOAc: cyclohexane = 20:90). **^{1}H NMR** (500 MHz, $CDCl_3$) δ 7.54 (d, *J* = 7.5 Hz, 1H), 7.45 (t, *J* = 7.6 Hz, 2H), 7.40-7.37 (m, 1H), 7.30-7.25 (m, 3H), 6.93 (t, *J* = 7.2 Hz, 1H), 6.76 (d, *J* = 8.1 Hz, 1H), 4.18 (d, *J* = 10.2 Hz, 1H), 3.64 (dd, *J* = 11.2, 2.6 Hz, 1H), 3.58-3.55 (m, 1H), 3.15 (dd, *J* = 11.6, 4.5 Hz, 1H), 2.87 (s, 3H). **^{13}C NMR** (125 MHz, $CDCl_3$) δ 177.8, 175.9, 148.6, 132.1, 130.4, 129.1 (2C), 128.8, 128.6, 126.5 (2C), 119.8, 118.7, 112.7, 50.8, 43.7, 42.2, 39.7. **FT-IR (ν/cm^{-1}, neat):** 2967, 2862, 2811, 1706, 1597, 1497, 1391, 1196, 751.

4.9. Synthesis of 3,5-diphenyl-1,2,4-thiadiazole (7)

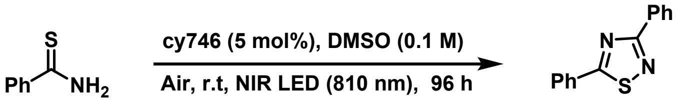

6 7, 77%

Benzothioamide **6** (30 mg, 0.22 mmol, 1 equiv.), cy746 (7 mg, 0.011 mmol, 5 mol%) and DMSO (1.9 mL) were charged in a reaction glass tube with magnetic stirring bar. The reaction mixture was stirred under air atmosphere and near infrared LED (810 nm) irradiation (3 cm away from the light source) at room temperature for 96 h. The crude mixture was extracted with ethyl acetate (3 x 10 mL). The combined organic layers were dried over Na_2SO_4, filtered and concentrated under vacuum pressure. The crude was purified by column chromatography on silica gel (EtOAc: cyclohexane = 5:95) to give 3,5-diphenyl-1,2,4-thiadiazole **7** (m = 20 mg, yield = 77%) as a white solid. Data are conformed to the litereature.[13] **R_f** = 0.42 (EtOAc: cyclohexane = 5:95). **^{1}H NMR** (500 MHz, $CDCl_3$) 8.40 (dd, *J* = 7.4, 1.4 Hz, 2H), 8.06 (dd, *J* = 7.7, 1.7 Hz, 2H) 7.55-7.49 (m, 6H). **^{13}C NMR** (125 MHz, $CDCl_3$) δ 188.3, 174.0, 133.0, 132.7, 132.1, 130.1, 130.5, 129.4 (2C), 129.1, 128.9 (2C), 128.5, 127.6. **FT-IR (ν/cm^{-1}, neat):** 2933, 2254, 1719, 1266, 1101, 906, 731.

4.10. *Synthesis of 4-methoxyphenol (9)*

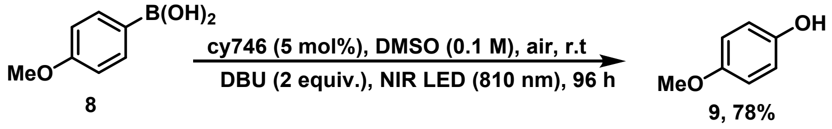

(4-Methoxyphenyl)boronic acid **8** (30 mg, 0.2 mmol, 1 equiv.), DBU (46 mg, 0.3 mmol, 1.5 equiv.), cy746 (6 mg, 0.01 mmol, 5 mol%) and DMSO (2 mL) were charged in a reaction glass tube with magnetic stirring bar. The reaction mixture was stirred under air atmosphere and near infrared LED (810 nm) irradiation (3 cm away from the light source) at room temperature for 96 h. The crude mixture was extracted with ethyl acetate (3 x 10 mL). The combined organic layers were dried over Na_2SO_4, filtered and concentrated under vacuum pressure. The crude was purified by column chromatography on silica gel (EtOAc: cyclohexane = 5:95) to give 4-methoxyphenol **9** (m = 19 mg, yield = 78%) as a white solid. Data are conformed to the literature.[14] $\mathbf{R_f}$ = 0.25 (EtOAc: cyclohexane = 5:95). **^{1}H NMR** (500 MHz, $CDCl_3$) 6.78 (d, *J* = 3.18 Hz, 4H), 5.14 (bs, 1H) 3.77 (s, 3H). **^{13}C NMR** (125 MHz, $CDCl_3$) δ 153.8, 149.6, 116.2 (2C), 115.1 (2C), 56. **FT-IR (ν/cm^{-1}, neat):** 3389, 3344, 2951, 2833, 1503, 1440, 1208, 1111, 1030, 821.

4.11. *Synthesis of 5-hydroxyfuran-2(5H)-one (11)*

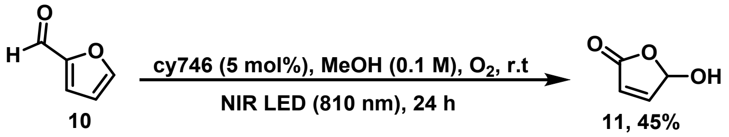

In a single-neck round-bottom flask, furfural **10** (34 µL, 0.42 mmol, 1 equiv.), cy746 (13 mg, 0.02 mmol, 5 mmol%) were added in dry methanol (4 mL). Then, O_2 was bubbled for 15 min and the mixture was stirred under 810 nm LED irradiation and O_2 (1 atm, balloon) for 24 h. The volatiles were removed under reduced pressure, 80 % of conversion was observed by ^{1}H NMR. The crude mixture was then purified by flash chromatography on silica gel (DCM pure) to give **11** as a yellow solid (m= 19 mg, yield = 45%). Data are conformed to the literature.[15] $\mathbf{R_f}$ = 0.8 (DCM) **^{1}H NMR** (500 MHz, $CDCl_3$) δ 7.29 (dd, *J* = 5.6, 1.2 Hz, 1H), 6.24 (dd, *J* = 5.6, 1.2 Hz, 1H), 6.23-6.21 (m, 1H), 4.06 (brs, 1H, OH) **^{13}C NMR** (125 MHz, $CDCl_3$) δ 170.8, 151.7, 125.0, 98.5. **FT-IR (ν/cm-1 , neat):** 3358, 2974, 2901, 1760, 1394, 1266, 1048, 880, 738.

4.12. *Synthesis of (Z)-1,2,3,4-Tetraphenyl-2-butene-1,4-dione (13)*

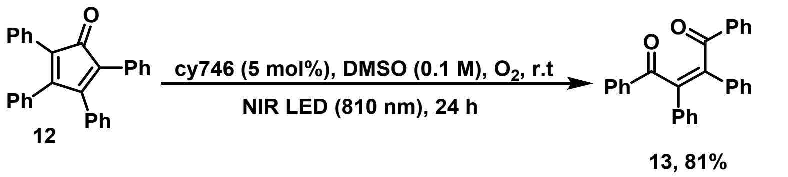

In a single-neck round-bottom flask, tetraphenyl-2,4-cyclopentadienone **12** (70 mg, 0.18 mmol, 1 equiv.), cy746 (6 mg, 0.01 mmol, 5 mmol%) were added in dry DMSO (2 mL). Then, O_2 was bubbled for 15 min and the mixture was stirred under 810 nm LED irradiation and O_2 (1 atm., balloon) for 24 h. Then, water was added to the mixture (5 mL), extraction with DCM (3 x 10 mL), and the combined organic phases were dried over anhydrous magnesium sulfate and evaporated under reduced pressure. The residue was purified by flash chromatography on silica gel (petroleum ether/AcOEt, 9:1) to give **13** as a white solid (m = 57 mg, yield = 81%). Data are conformed to the literature.[16] $\mathbf{R_f}$ = 0.5 (8:2

Petroleum Ether/AcOEt) **^{1}HNMR** (500 MHz, $CDCl_3$) δ 7.86-7.84 (m, 4H), 7.44-7.40 (m, 2H), 7.31 (t, *J* = 7.7 Hz, 4H), 7.21-7.15 (m, 10 H) **^{13}C NMR** (100 MHz, $CDCl_3$) δ 191.1(2C), 144.7(2C), 136.5(2C), 135. 4(2C), 133.1(2C), 130.1(4C), 130.0(4C), 128.8(4C), 128.5 (2C), 128.4(4C) **FT-IR (ν/cm-1 , neat):** 2971, 2901, 1658, 1579, 1444, 1258, 1037, 768, 692.

4.13. Synthesis of ethyl 5-phenyl-1H-pyrrole-2-carboxylate 15

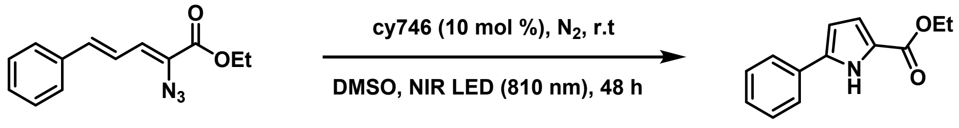

14 **15, 68%**

Ethyl (2E,4E)-2-methyl-5-phenylpenta-2,4-diene **14** (95 mg, 0.39 mmol, 1 equiv.), cy746 (25 mg, 0.039 mmol, 10 mol %)and 3.5 mL of dry DMSO were charged in a reaction glass tube with a magnetic stirring bar. The mixture was degassed by three cycles of freeze-pump-thaw and purged with N_2. Then the mixture was stirred and irradiated with a near infrared LED (810 nm) (approximately 3 cm away from the LED lamp) at room temperature for 48 h. After the reaction, H_2O was added. The resulting mixture was extracted with AcOEt (3 x 10 mL), washed with H_2O (2 x 10 mL), dried (Na_2SO_4), and filtered. The filtrate was concentrated in vacuo, and the crude residue was purified by column chromatography (1/9 : EtOAc/PE) to afford the pure product **15** (m= 57 mg, yield = 68%) as a white solid. Data are conformed to the literature.[17] **R_f** = 0.29 (1/9: EtOAc/PE). **^{1}H NMR** (400 MHz, $CDCl_3$) δ 9.52 (brs, 1H), 7.60-7.58 (m, 2H), 7.42-7.39 (m, 2H), 7.32-7.29 (m, 1H), 6.98-6.96 (dd, *J*= 3.7, 2.5 Hz, 1H), 6.55-6.54 (dd, *J*= 3.7, 2.8 Hz, 1H),4.34 (q, *J*= 14.2, 7 Hz, 2H), 1.38 (t, *J*= 7.1 Hz, 3H). **^{13}C NMR** (126 MHz, CDCl3) δ 161.5, 136.9, 131.5, 129.1 (2C), 127.9, 124.9 (2C), 123.5, 116.8, 108.1, 60.6, 14.6. **FT-IR (ν/cm-1 , neat):** 3315, 1680, 1262, 1162.

4.14. Synthesis of 3,3,3-trifluoroprop-1-ene-1,1-diyl)dibenzene 18

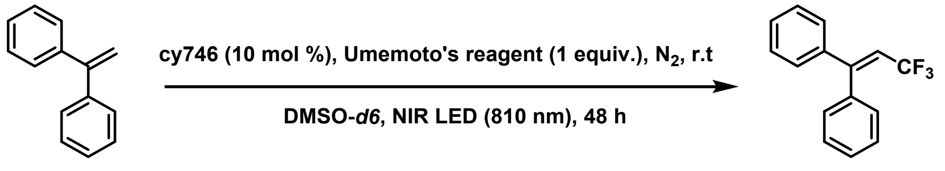

17 **18, 50 % NMR yield**

1,1-diphenylethylene **17** (23 mg, 0.13 mmol, 1 equiv.), Umemoto's reagent [CAS:131880-16-5] (44 mg, 0.13 mmol, 1 equiv.), cy746 (8 mg, 0.013 mmol, 10 mol %) were charged in a reaction glass tube with a magnetic stirring bar. The glass tube was placed under N_2. Then DMSO-*d6* (1 mL) was degassed by three cycles of freeze-pump-thaw before being added at the glass tube. The mixture was stirred and irradiated with a near infrared light source (810 nm) equipped with an adjustable collimation optic (3 cm away from the LED lamp) at room temperature. After 48 h of reaction, anisole (12 µL, 0.13 mmol, 1 equiv.) was added as an internal standard to the glass tube. NMR yield = 50%. Data are conformed to the literature.[18]

4.15. Synthesis of 4,4'-(3,3,3-trifluoroprop-1-ene-1,1-diyl)bis(methoxybenzene) 20

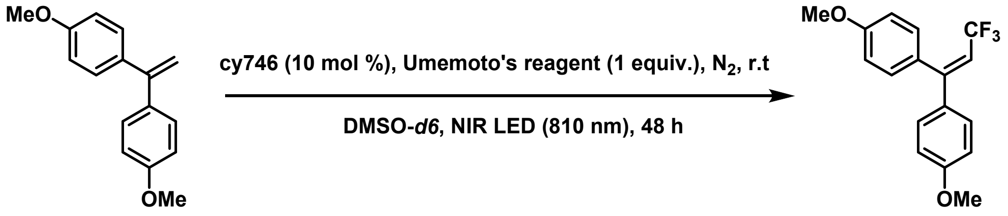

19 **20, 66 % NMR yield**

1,1-bis(4-methoxyphenyl)ethene **20** (24 mg, 0.1 mmol, 1 equiv.), Umemoto's reagent [CAS:131880-16-5] (34 mg, 0.1 mmol, 1 equiv.), cy746 (6 mg, 0.01 mmol, 10 mol %) were charged in a reaction gfzlass tube with a magnetic stirring bar. The glass tube was placed under N_2. Then DMSO-*d6* (1 mL) was degassed by three cycles of freeze-pump-thaw before being added at the glass tube. The mixture was stirred and irradiated with a near infrared light source (810 nm) equipped with an ajustable collimation optic (3 cm away from the LED lamp) at room temperature. After 48 h of reaction, durene (14 mg, 0.1 mmol, 1 equiv.) was added as an internal standard to the glass tube NMR yield = 66 %. Data are conformed to literature.[19]

4.16. Synthesis of 3,3,3-trifluoro-1,1-diphenylpropan-1-ol 21

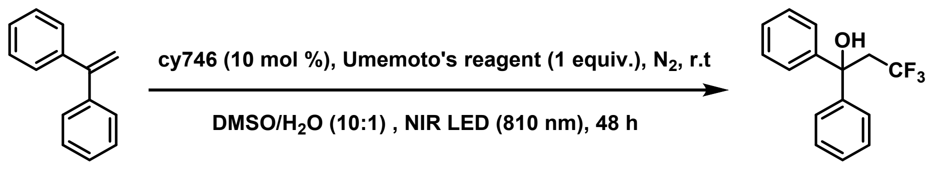

17 **21, 46 %**

1,1-diphenylethylene **16** (23 mg, 0.13 mmol, 1 equiv.), Umemoto's reagent [CAS:131880-16-5] (44 mg, 0.13 mmol, 1 equiv.), cy746 (8 mg, 0.013 mmol, 10 mol%) were charged in a reaction glass tube with a magnetic stirring bar. The glass tube was placed under N_2. Then DMSO (1 mL) was degassed by three cycles of freeze-pump-thaw before being added at the glass tube with 10 % of H_2O (0.1 mL). The mixture was stirred and irradiated with a near infrared LED (810 nm) equipped with an adjustable collimation optic (approximately 3 cm away from the LED lamp) at room temperature for 48 h. After the reaction, H_2O was added. The resulting mixture was extracted with Et_2O, washed with H_2O, dried (Na_2SO_4), and filtered. The filtrate was concentrated *in vacuo.* The crude was purified by column chromatography on silica gel (PE → EtOAc/PE = 1/9, to give 3,3,3-trifluoro-1,1-diphenylpropan-1-ol **21** (m = 15 mg, yield = 46 %) as a brown oil. Data are conformed to the literature.[20] **R_f** = 0.23 (EtOAc/PE = 1/9). **^{1}H NMR** (400 MHz, $CDCl_3$) δ 7.43 (m, 4H), 7.34 (m, 4H), 7.26 (m, 2H), 3.21 (q, *J*= 10.6 Hz, 2H), 2.62 (m, 1H). **^{13}C NMR** (126 MHz, $CDCl_3$) 145.2 (2C), 128.6 (4C), 127.7 (2C), 125.7 (4C), 124.7(q, *J*= 278 Hz), 75.8, 45.1 (q, *J*= 25.5 Hz)

5. Cy746 stability

Procedure: Cy746 (6 mg, 0.01mmol) was dissolved in 0.6 mL of DMSO-*d6* and added into a NMR tube. The mixture was irradiated with a near infrared LED (810 nm) and ^{1}H NMR were recorded at t = 1h, t = 24h, t = 72h. No degradation was observed

^{1}H NMR spectrum of cy746 after 1h of irradiation (LED-810 nm) in DMSO-*d6*

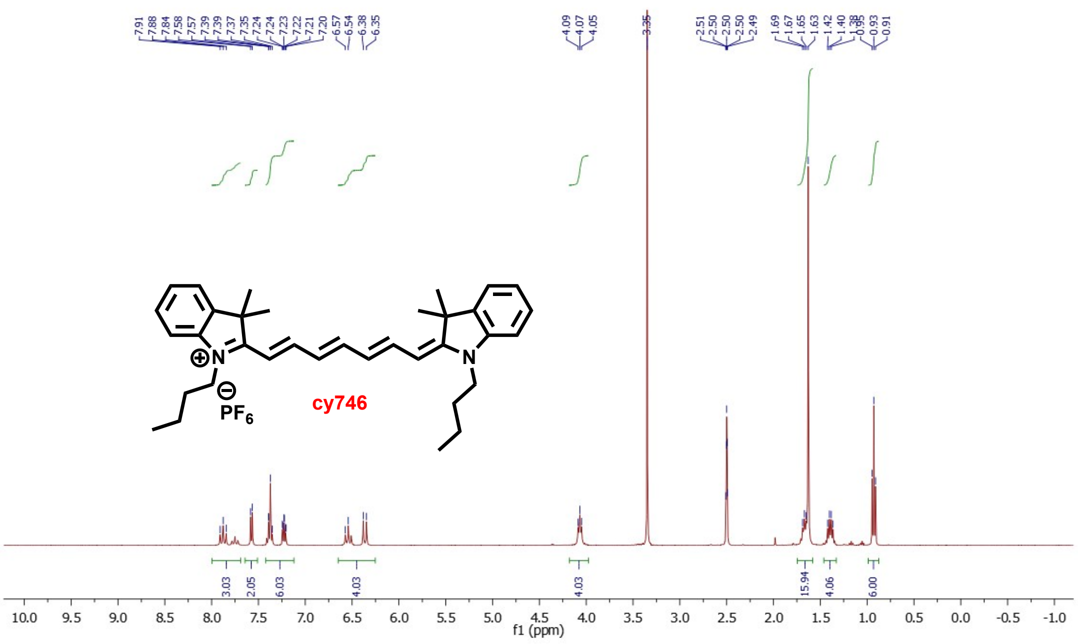

^{1}H NMR spectrum of cy746 after 24 h of irradiation (LED-810 nm) in DMSO-*d6*

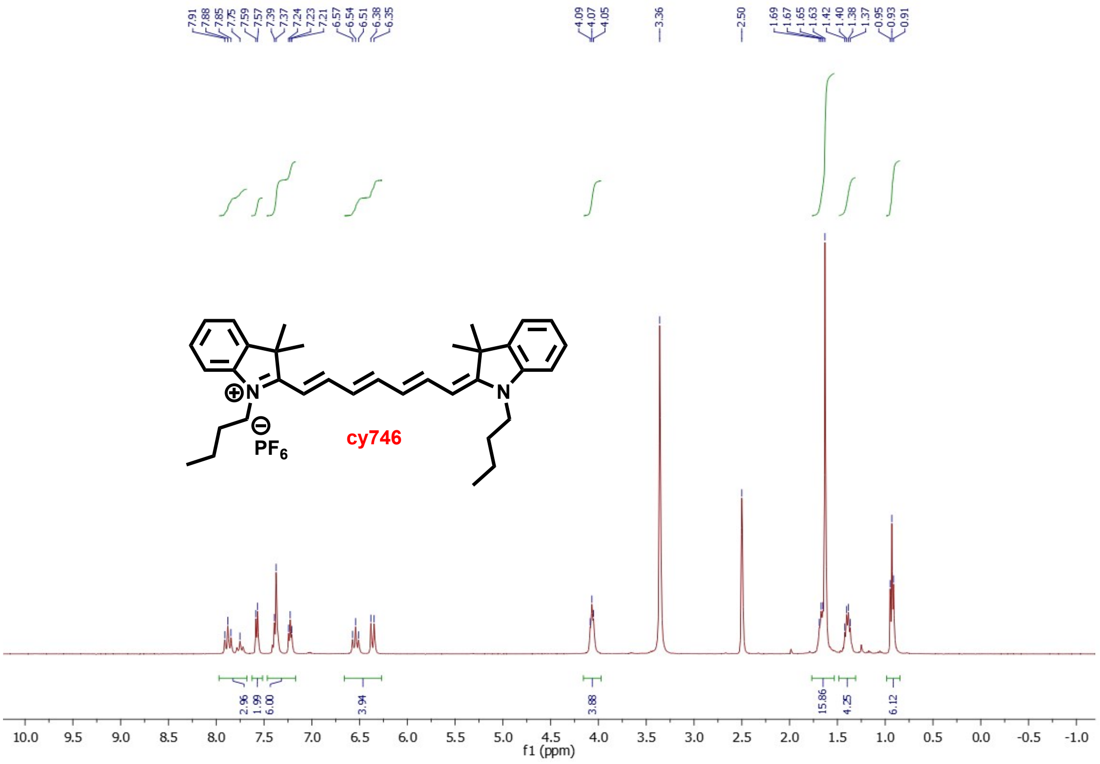

^{1}H NMR spectrum of cy746 after 72 h of irradiation (LED-810 nm) in DMSO-*d6*

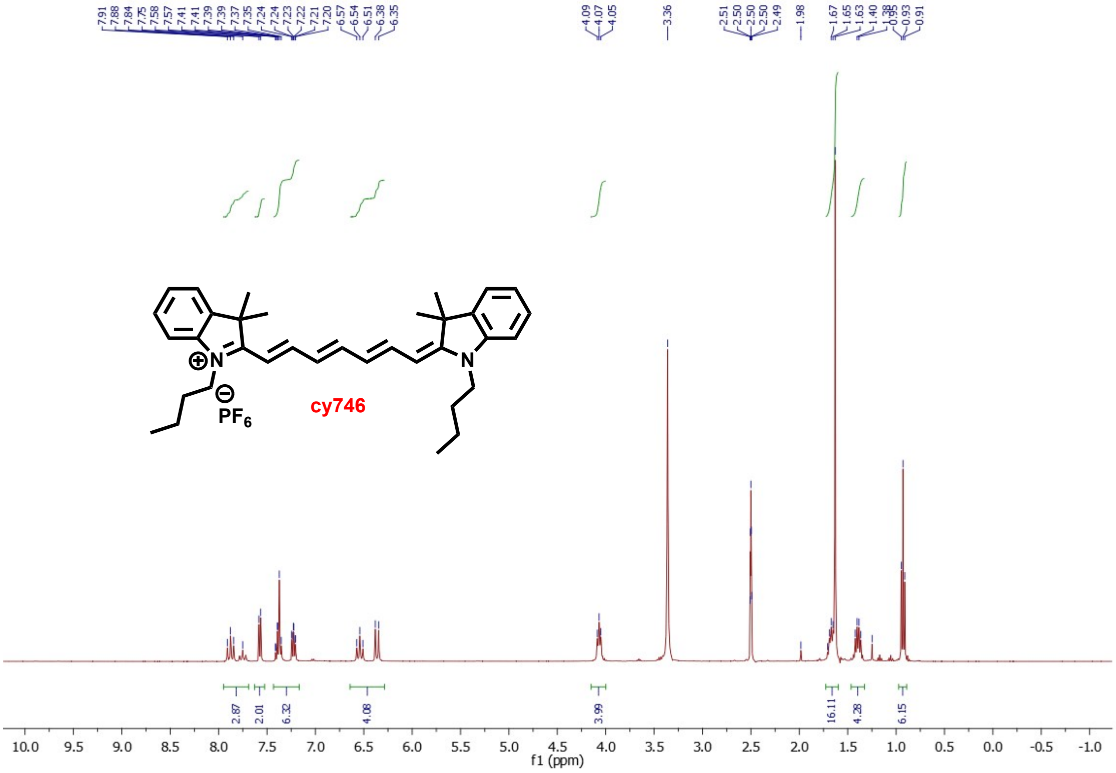

6. Aza-Henry reaction: Kinetic experiments

6.1. Procedure

Kinetic experiments were run on 0.13 mmol of **1a**, 10 equiv. of CH_3NO_2 5 mol% of photocatalyst in DMSO-*d6* (1.3 mL). Each reaction was irradiated by one LED (3 cm away from the tube) at the optimal wavelength of the corresponding photocatalyst. Conversions of **1a** into **2a** were measured by ^{1}H NMR at t = 0, t = 1 h, t = 2 h, t = 3h, t = 24 h.

6.2. Kinetic experiments with cy746 : Irradiation LED-810 nm

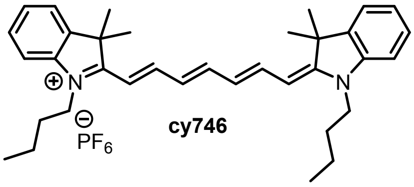

time (h)	Conversion (%)
0	0
1	9
2	14
3	26
24	98

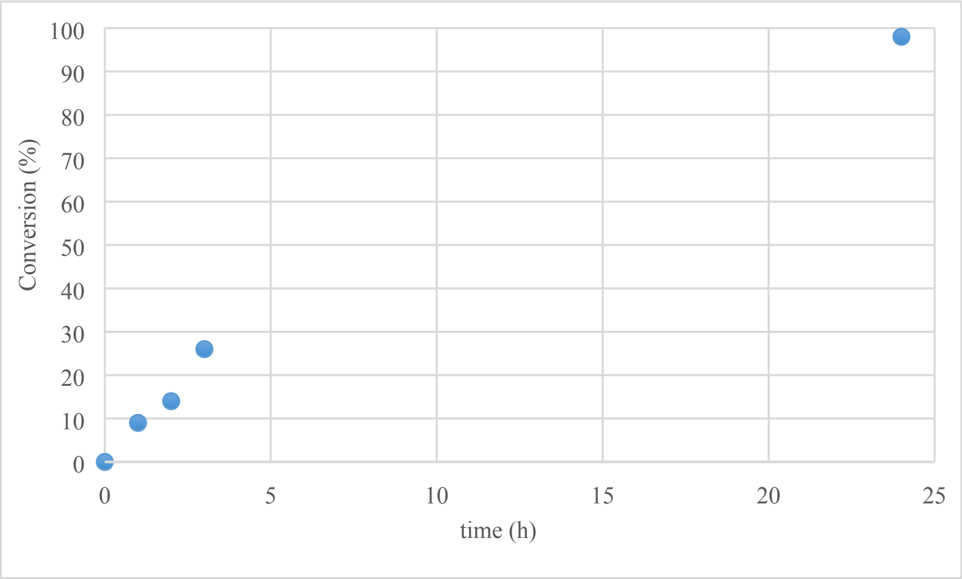

6.3. Kinetic experiments with Rose Bengal : Irradiation LED-565 nm

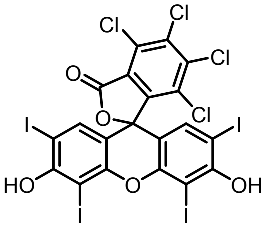

Rose Bengal

time (h)	Conversion (%)
0	0
1	52
2	70
3	82
24	100

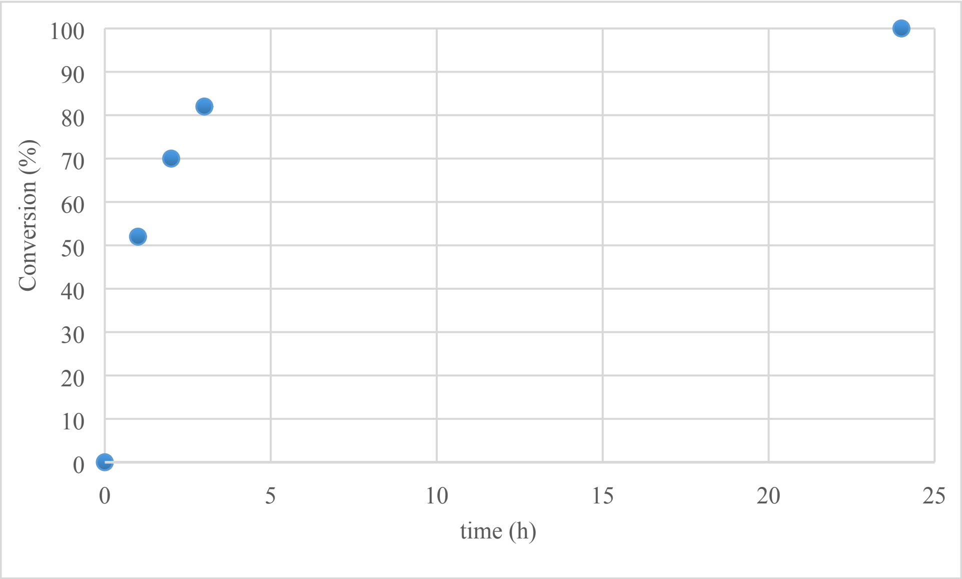

6.4. Kinetic experiments with Eosin Y : Irradiation LED-565 nm

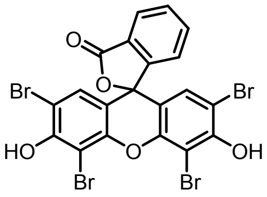

Eosin Y

time (h)	Conversion (%)
0	0
1	40
2	61
3	71
24	100

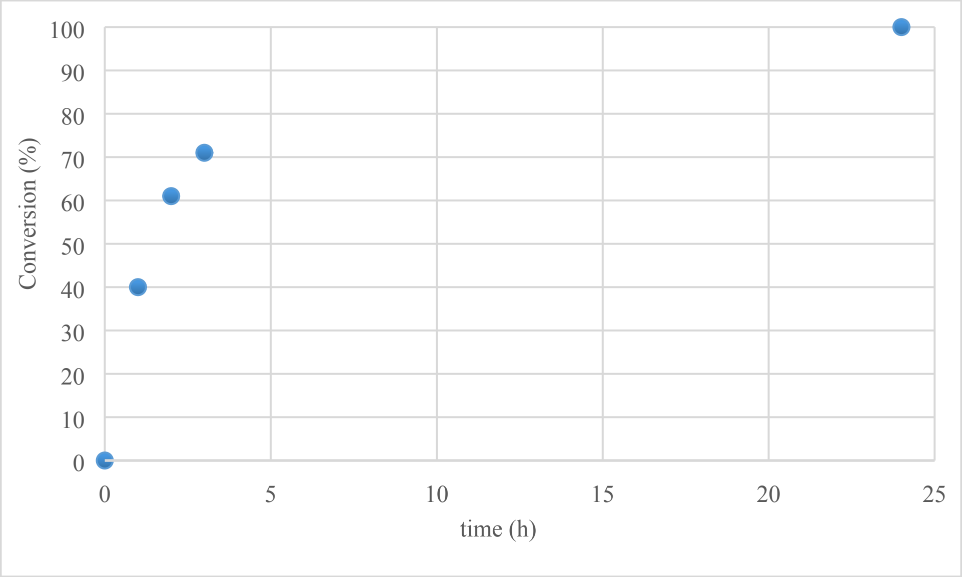

6.5. Kinetic experiments with $Ru(bpy)_3Cl_2$: Irradiation LED-455 nm

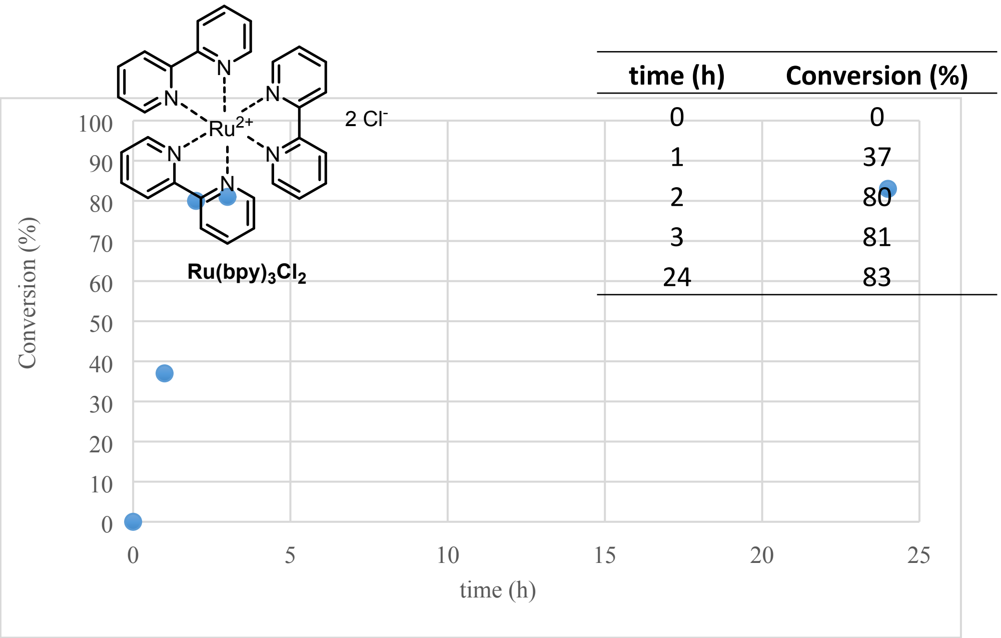

$Ru(bpy)_3Cl_2$

time (h)	Conversion (%)
0	0
1	37
2	80
3	81
24	83

6.6. Kinetic profiles comparisons

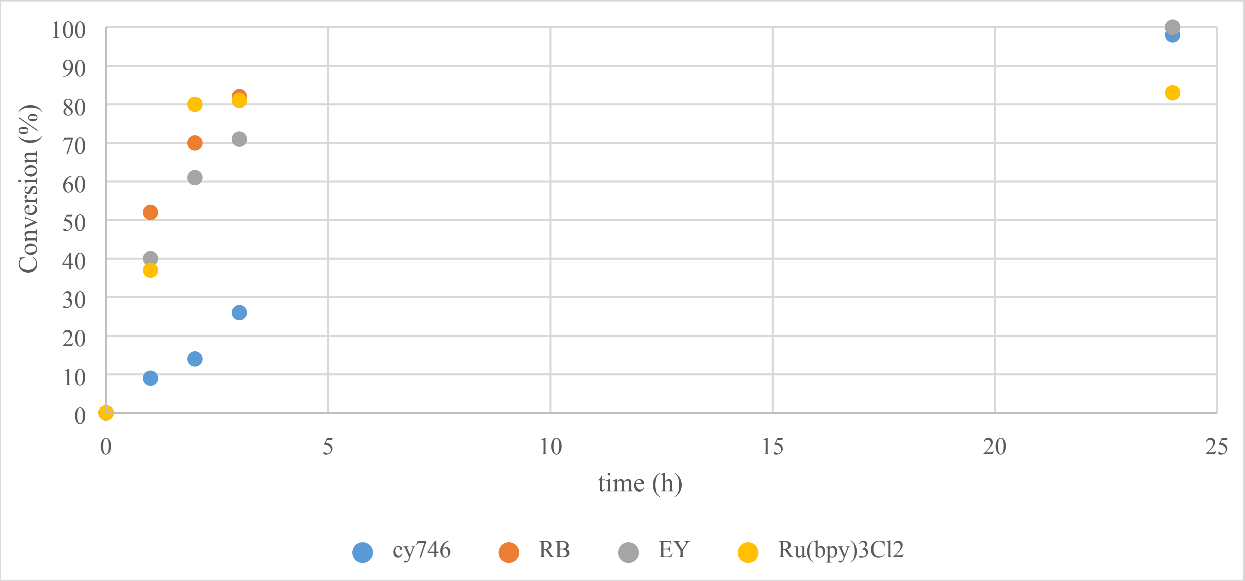

7. Stern-Volmer experiments

Fluorescence quenching studies were performed using a SpectraMax ID3 UV-Visible multimode microplate reader from Molecular Devices.

To a 20 µM solution of organic photocatalysts (IR-746, Eosin Y, Rose Bengal, $Ru(bpy)_3Cl_2$) in DMSO (100ml) are added consecutively 0, 20, 30 35, 40, 45 mM of 2-(4-methoxyphenyl)-1,2,3,4-tetrahydroisoquinoline **1a**. The mixture was charged in a reaction glass tube with magnetic stirring bar. These solutions containing quencher **1a** and IR-746, eosin Y, Rose Bengal or $Ru(bpy)_3Cl_2$ in DMSO were irradiated respectively at 690, 470, 510 and 430 nm and the emission spectra recorded. Plots of fluorescence intensity *versus* wavelength are presented below.
A Stern-Volmer curve is produced by plotting the normalized maximum intensity versus quencher concentration **1a**.

7.1. Stern Volmer experiment of cy746

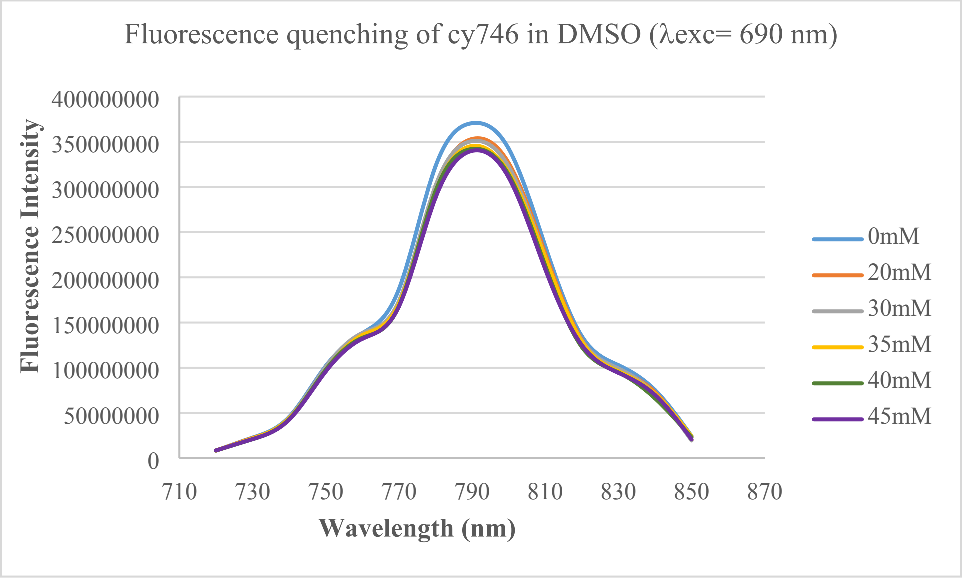

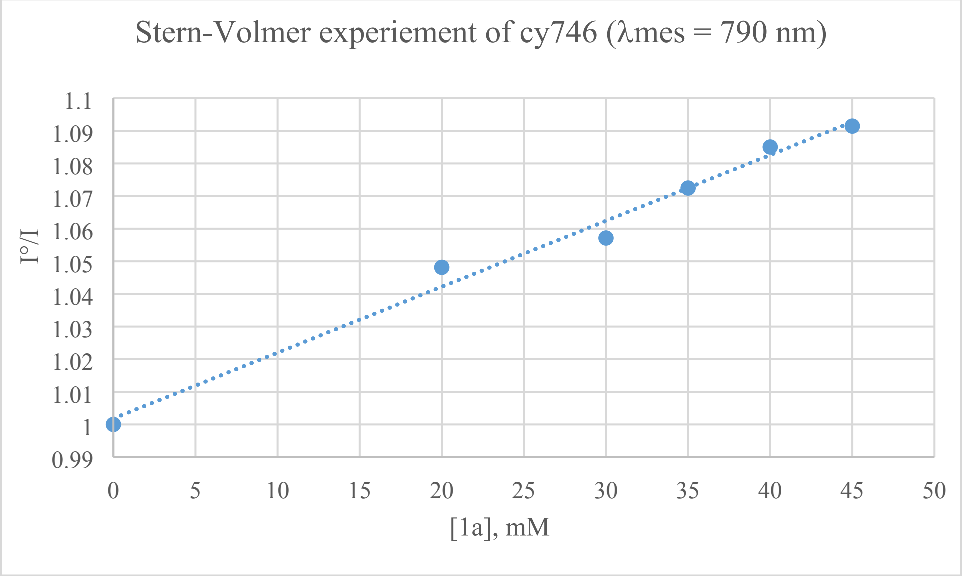

7.2. Stern Volmer experiment of Eosin Y

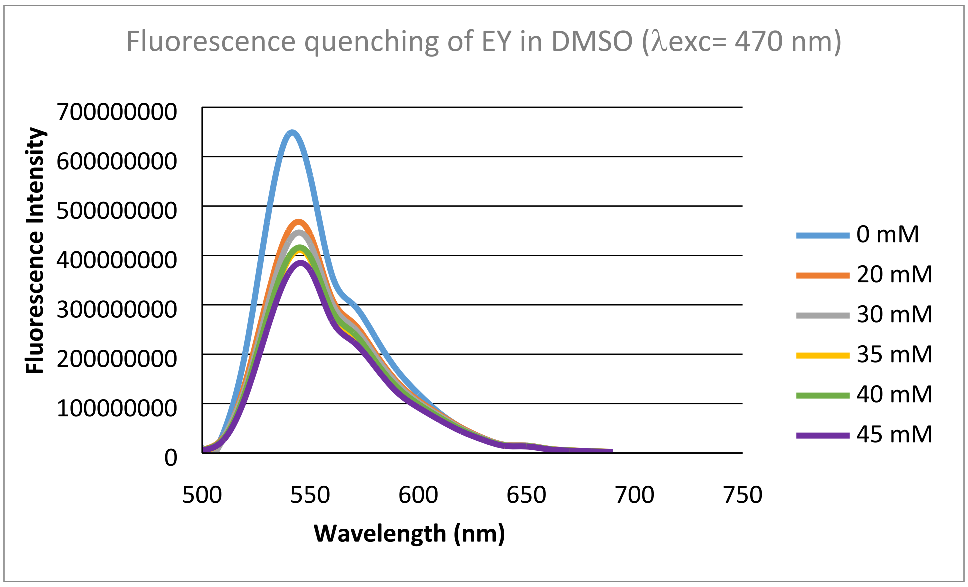

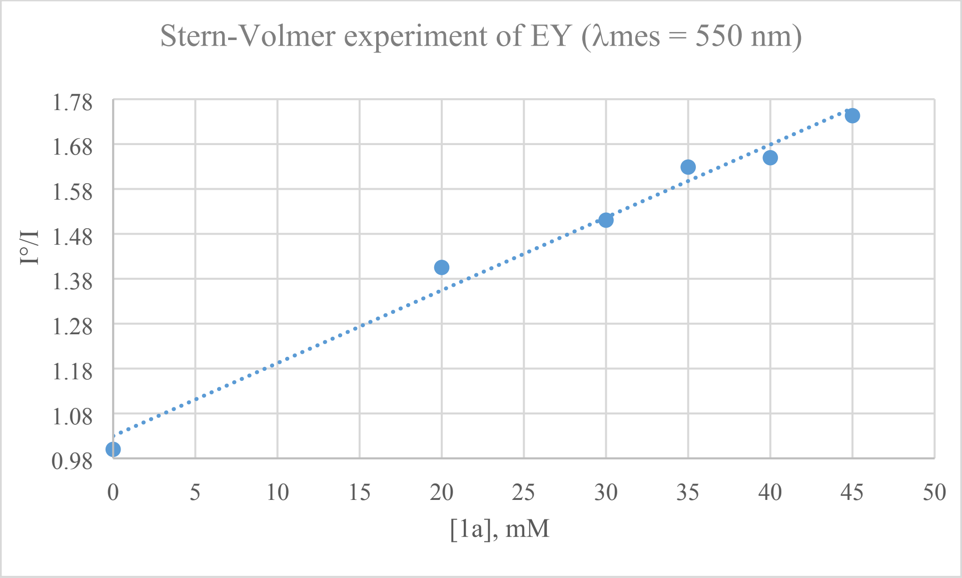

7.3. Stern Volmer experiment of Rose Bengal

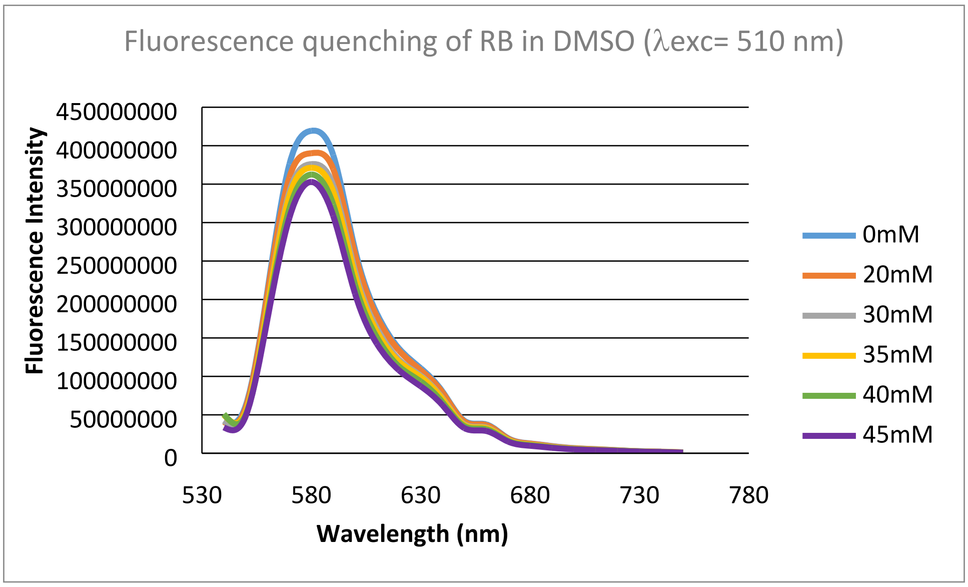

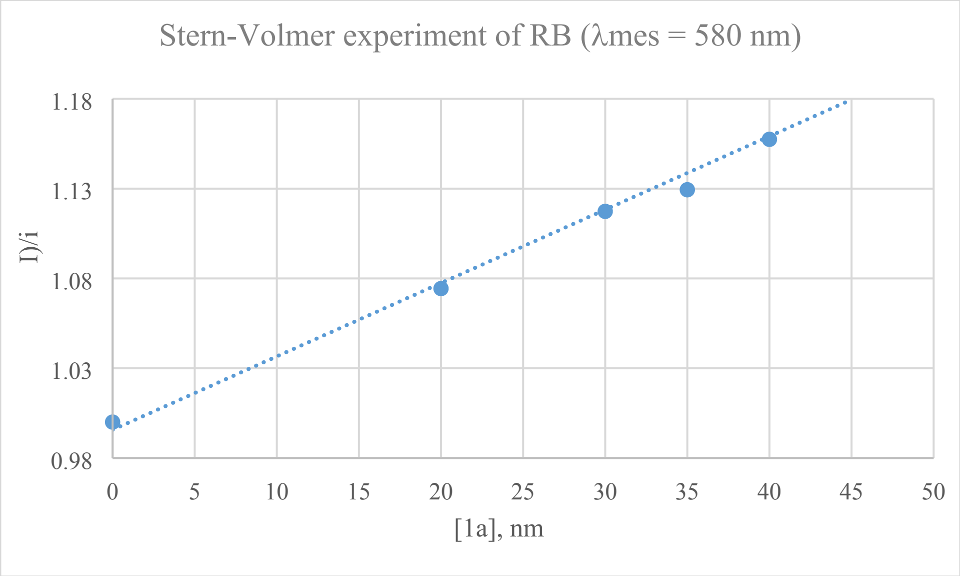

7.4. *Stern Volmer experiment of Ru(bpy)$_3$Cl$_2$*

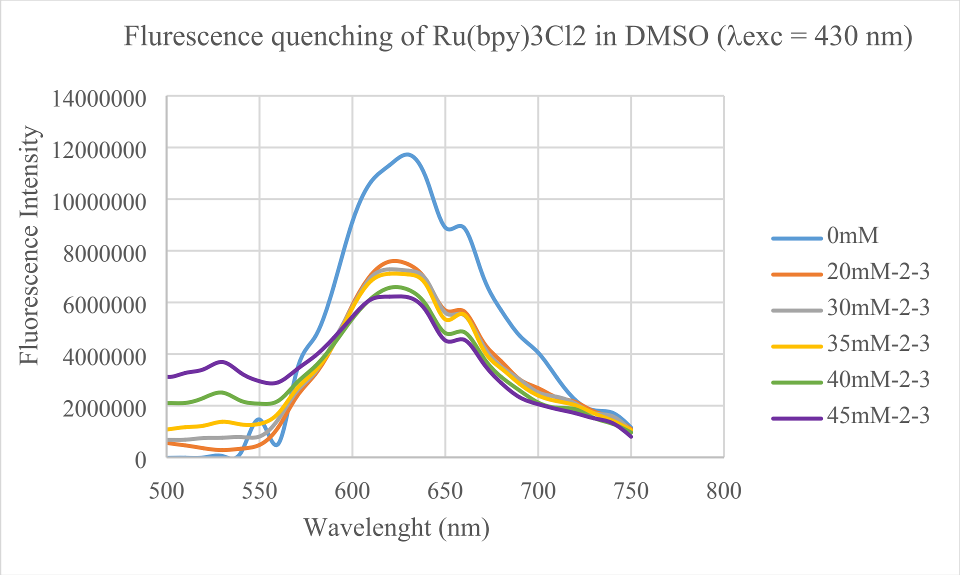

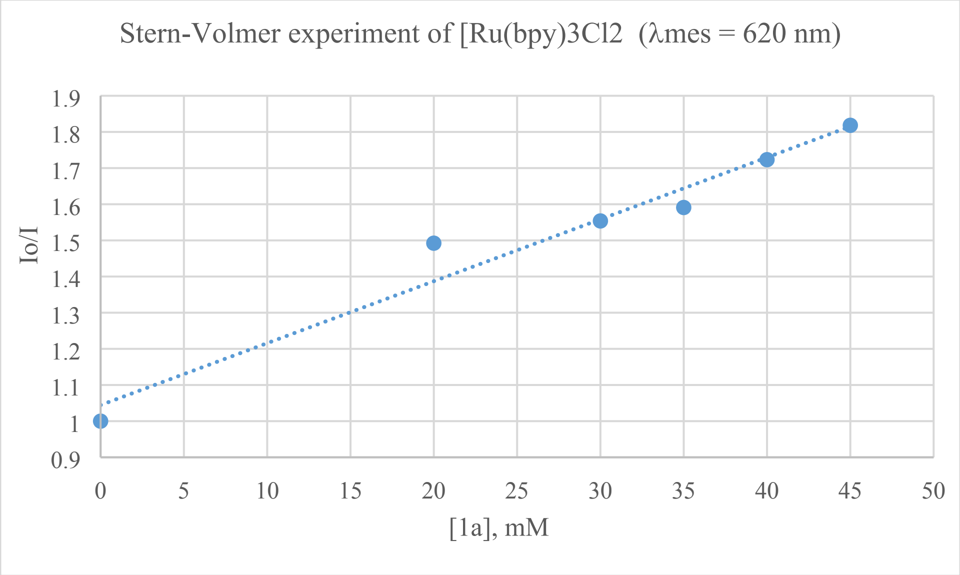

*7.5. Stern Volmer experiment of cy746 with benzencarbothioamide **6***

The experimental conditions are similar to those used with tetrahydroisoquinoline **1a** (section 7.1).

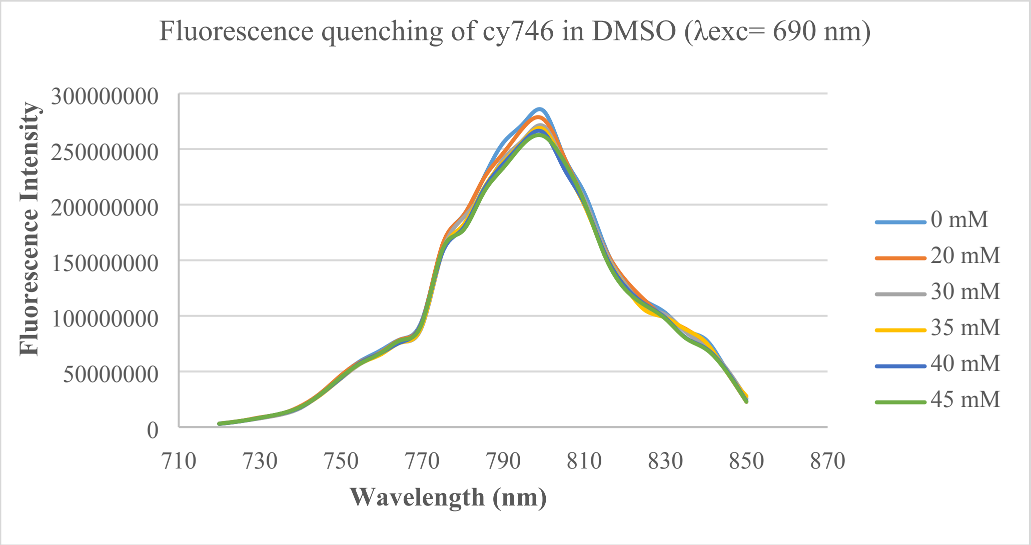

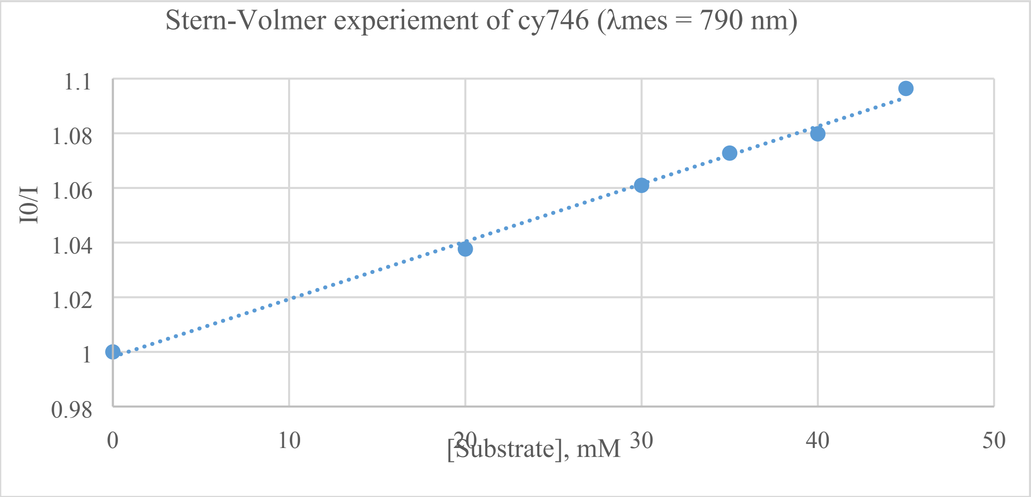

8. Proposition of two mechanistic pathways

The reaction mechanisms of cy746 has not been fully demonstrated but we can share in two distinctive pathways according to the catalyst behavior. Indeed, cyanines can be involved in single electron transfer through redox processes as well as energy transfer leading to photosensitization.

8.1. Single Electron Transfer pathway

Under Near Infra-red irradiation at 810 nm, cy746 could access an excited state that can be quench by organic molecules leading either to oxidation or reduction pathways. Concerning the aza-Henry reaction, we demonstrated by Stern-Volmer experiment that tetrahydroisoquinoline **1a** is able to reductively quench cy746* to generate the corresponding amino radical cation (see section 7.1 page S23)[21]. Similar quenching was observed with benzencarbothioamide **6** (see section 7.5 page S27). In the presence of oxygen, **cy746**$^{\cdot-}$ could be oxidized to the **cy746** ground state and the superoxide anion

which can proceed to a hydrogen atom abstraction and generate the iminium salt. The importance of oxygen is demonstrated by the low conversion (5%), observed when O_2 is omitted (see Table 1, entry 15 and ^{1}H NMR spectrum below). Without oxygen, the recovery of the catalyst ground state is not possible.

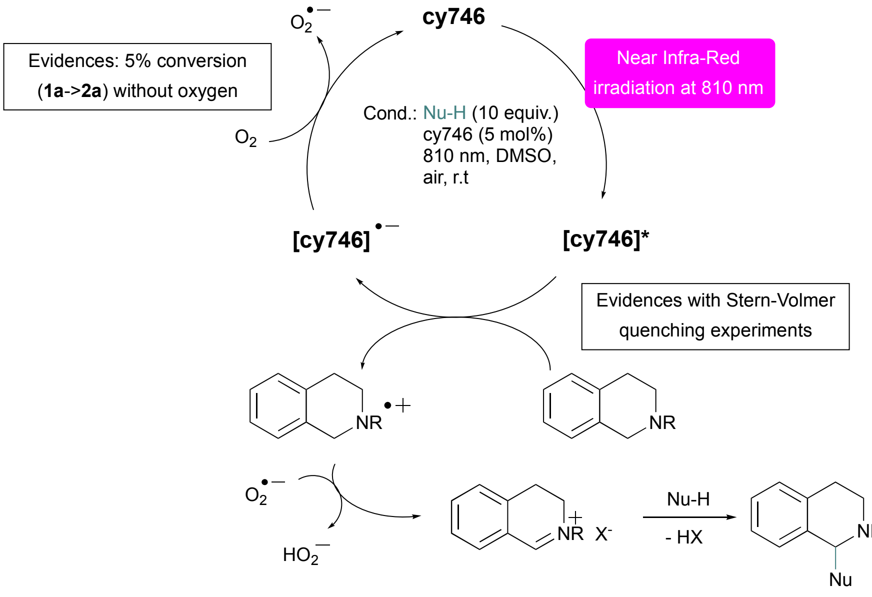

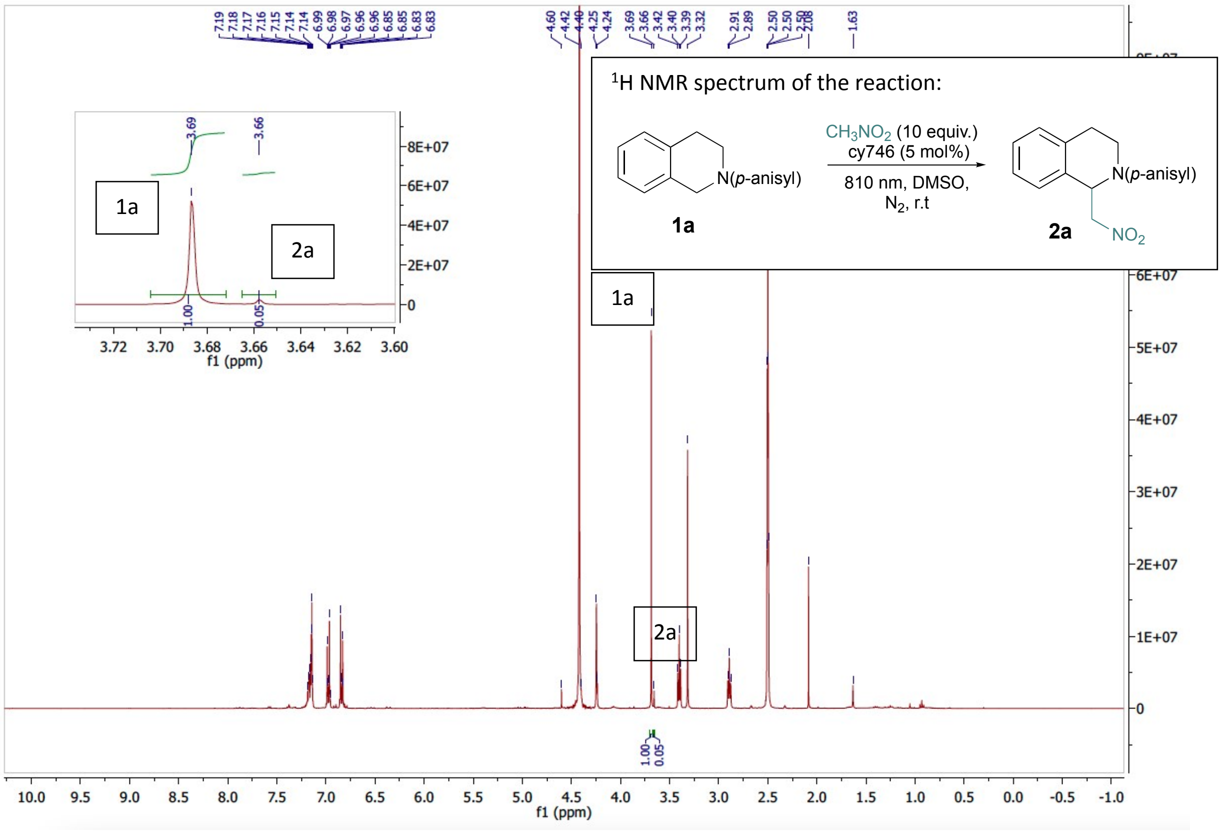

EDA complex investigation

UV-Vis absorption spectroscopy of **cy746** and variable amount of **1a** does not give evidence for the formation of EDA complex, which could modify the absorption properties of **cy746**. Then, we can exclude such interaction in the reaction pathway. Conditions: [**cy746**] = 20μM in DMSO and [**1a**] = from 20μM to 0.4 mM in DMSO. Green curve is [**1a**] = 1μM.

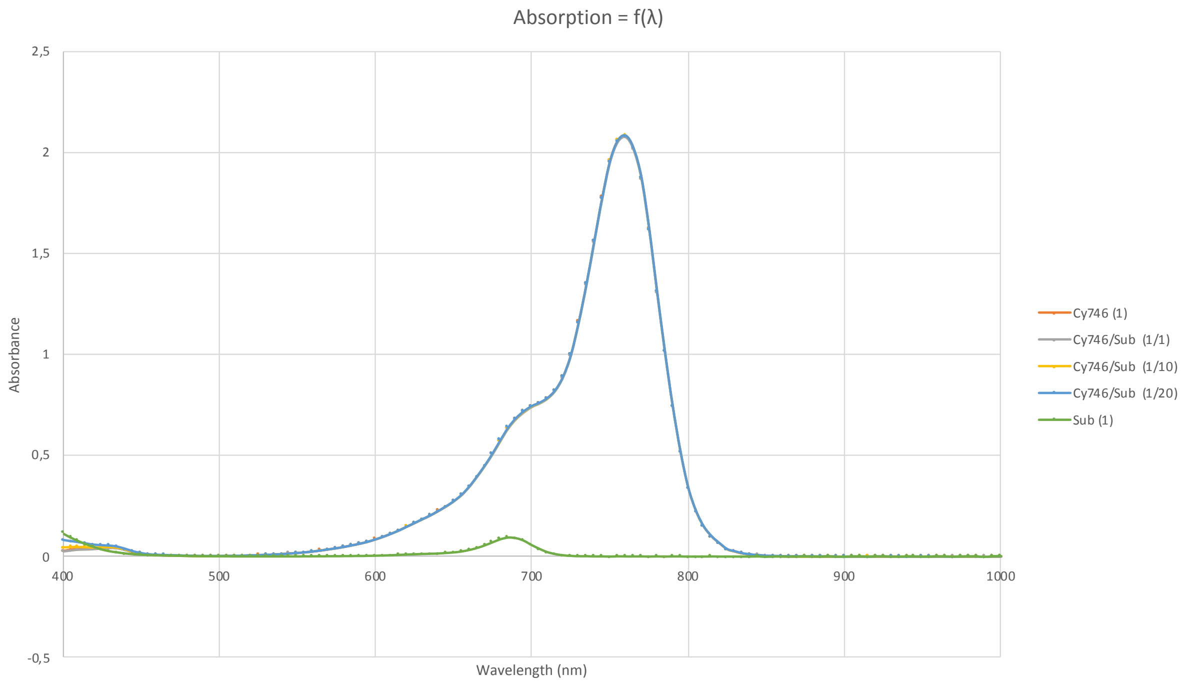

8.2. Energy transfer pathway

Cyanines are known to photosensitize triplet oxygen into singlet oxygen through energy transfer. This property has been extensively developed for medicinal applications like photodynamic therapy [22]. Thus, this mechanistic pathway can be proposed for the oxidation of boronic acid **8** and carbonyl derivatives **10** and **12**. Similar energy transfer process can be proposed for the formation of **15** from **14**.

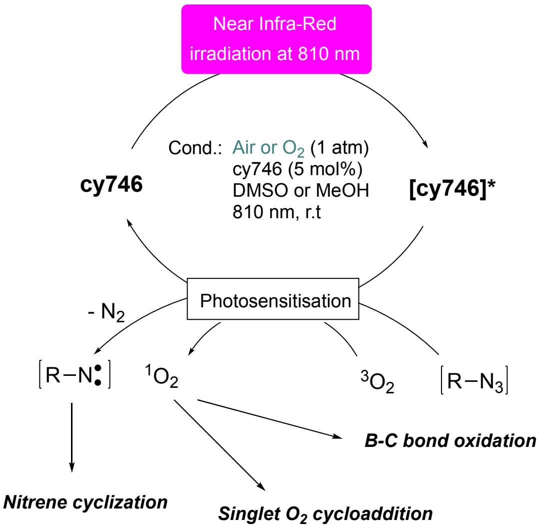

9. References

[1]. Spectra and data sheets are available on the website of Thorlab : https://www.thorlabs.com/

[2]. Z. Li, C.-J. Li, *J. Am. Chem. Soc.* **2005**, *127*, 6968-6969.

[3]. S.M. Soria-Castro, B. Lebeau, M. Cormier, S. Neunlist, T. J. Daou, J.-P. Goddard *Eur. J. Org. Chem.* **2020**, 1572–1578

[4]. D. Chang, D. Zhu, L. Shi, *J. Org. Chem.* **2015**, *80*, 5928-5933.

[5]. Z-B. Chen, D. Hong, Y-G. Wang, *J. Org. Chem.* **2009**, *74*, 903-905.

[6]. T. W. Liwosz, S.R. Chemler, *Chem. Eur. J.* **2013**, *19*, 12771–12777.

[7]. Baslé. O, Chao-Jun.Li, *Green Chem.* **2007**, *9*, 1047-1050

[8]. Q. Liu, Y-N. Li, H-H. Zhang, B. Chen, C-H. Tung, L-Z. Wu. *Chem. Eur. J.* **2012**, *18*, 620-627.

[9]. W. Fu, W. Guo, G. Zou, C. Xu *Journal of Fluorine Chemistry,* **2012**, *140*, 88-94

[10]. M. Rueping, S. Zhu, R. M. Koenigs *Chem. Commun.,* **2011**, *47*, 12709-12711

[11]. H. Durga, B. Koenig, *Org. Lett.* **2011**, *13*, 3852-3855

[12]. (a) J. Mateos, F. Rigodanza, A. Vega-PeÇaloza, A. Sartorel, M.Natali, T. Bortolato, G. Pelosi, X. Companyl, M. Bonchio, L. Dell'Amico *Angew. Chem. Int. Ed*. **2020**, *59*, 1302-1312 (b) T. Mandal, S. Das, S. De Sarkar, *Adv. Synth. Catal.* **2019**, *361*, 3200-3209.

[13]. A-H. A. Shah, Z. A. Khan, N. Choudhary, C. Lohölter, S. Schäfer, G.P. L. Marie, U. Farooq, B. Witulski, T. Wirth. *Org. Lett.* **2009**, *11*, 3578-3581

[14]. E. J. Rayment, N. Summerhill, E. A. Anderson, *J. Org. Chem*. **2012**, *77*, 7052-7060

[15]. Wahlen, J.; Moens, B.; De Vos, D. E.; Alsters, P. L.; Jocobs, P. A. *Adv. Synth. Catal.* **2004**, *346*, 333-338

[16]. Carney, J. M.; Hammer, R. J.; Hulce, M.; Lomas, C. M.; Miyashiro. *Synthesis* **2012**, *44*, 2560–2566

[17]. T. Elder, L. C. Gregory, A. Orozco, J.L. Pflug, P.S. Wiens, T.J. Wilkinson *Synth. Commun.* **1989**, *19*, 763-767

[18]. R. Tomita, Y. Yasu, T. Koike, M.Akita, *Beilstein J. Org. Chem.* **2014**, *10*, 1099-1106

[19]. C. Xu, W. Huang, R. Zhang, C. Gao, Y. Li, M. Wang, *J. Org. Chem.* **2019**, *84*, 14209-14216.

[20]. Y. Yasu, T. Koike, M. Akita, *Angew. Chem. Int. Ed.* **2012**, *51*, 9567-9571.

[21]. a) J. Hu, J. Wang, T. H. Nguyen, N. Zheng *Beilstein J. Org. Chem.* **2013**, *9*, 1977–2001. b) W. Luo, J.-D. Yang, J.-P. Cheng *iScience* **2020,** *23*, 10085.

[22]. E. Delaey, F. van Laar, D. De Vos, A. Kamuhabwa, P. Jacobs, P. de Witte *J. Photochem. Photobiol. B: Biol.* **2000**, *55*, 27-36 and references cited herein.

10. 1H and 13C NMR Spectra of Products

2-(4-methoxyphenyl)-1,2,3,4-tetrahydroisoquinoline (1a)

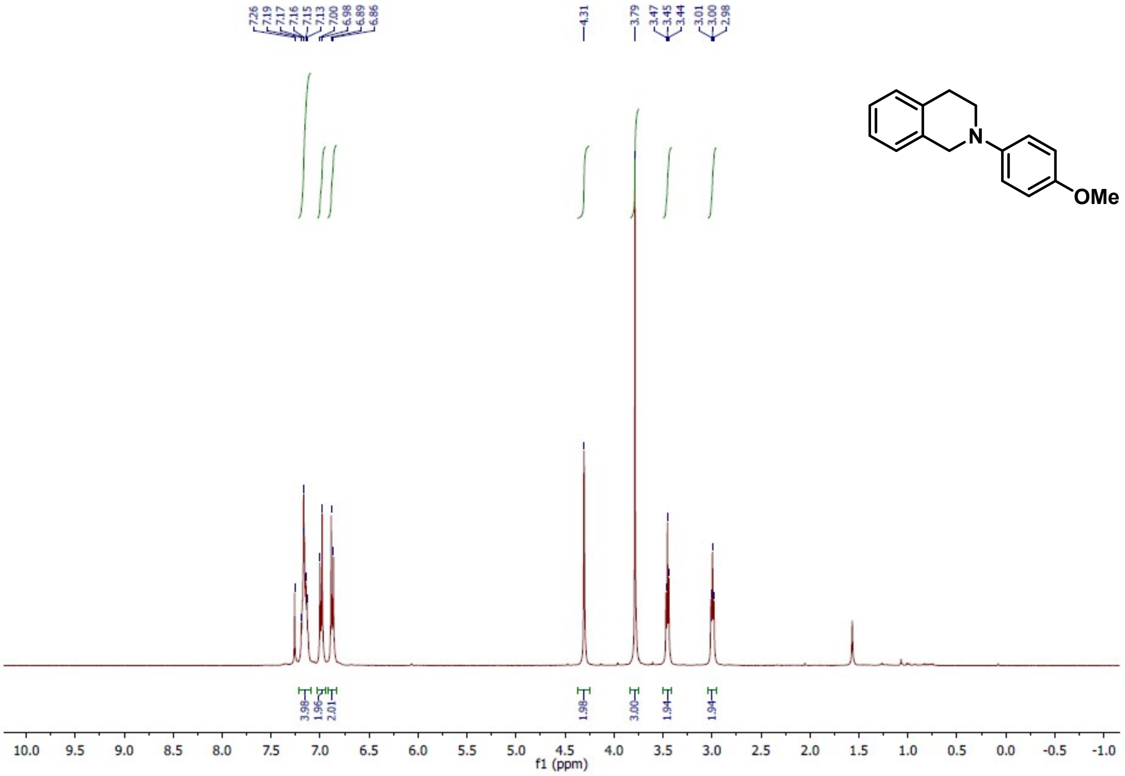

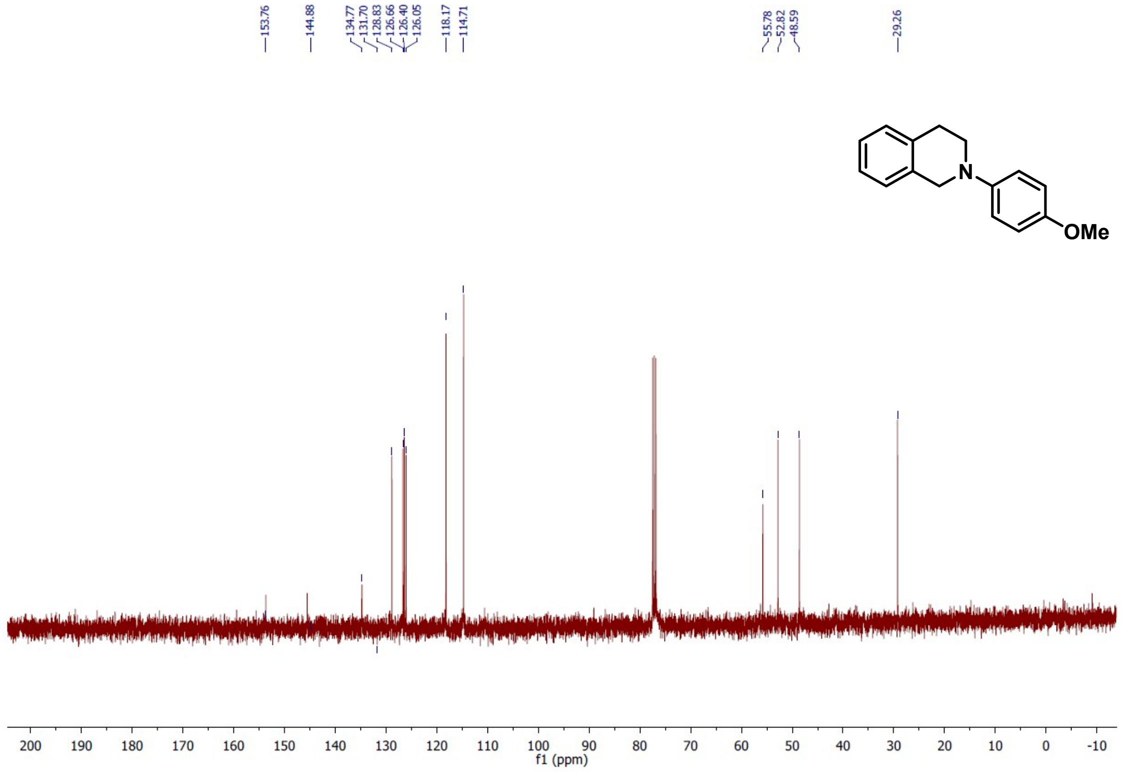

2-(p-tolyl)-1,2,3,4-tetrahydroisoquinoline (1b)

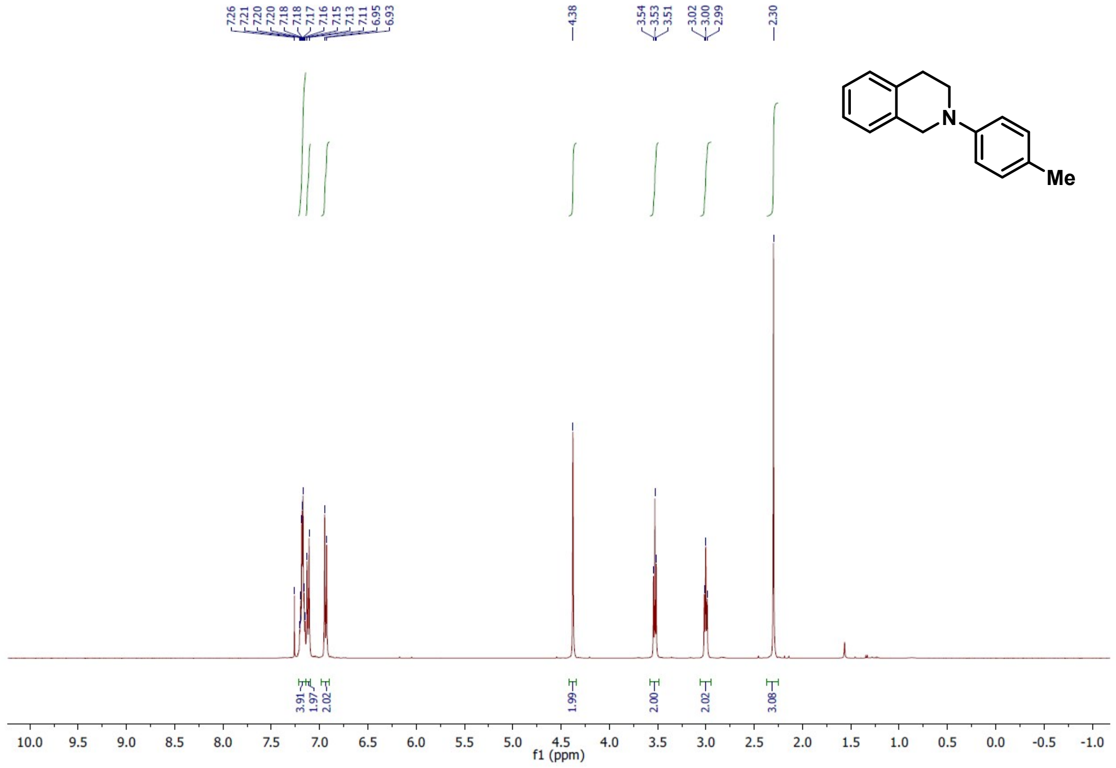

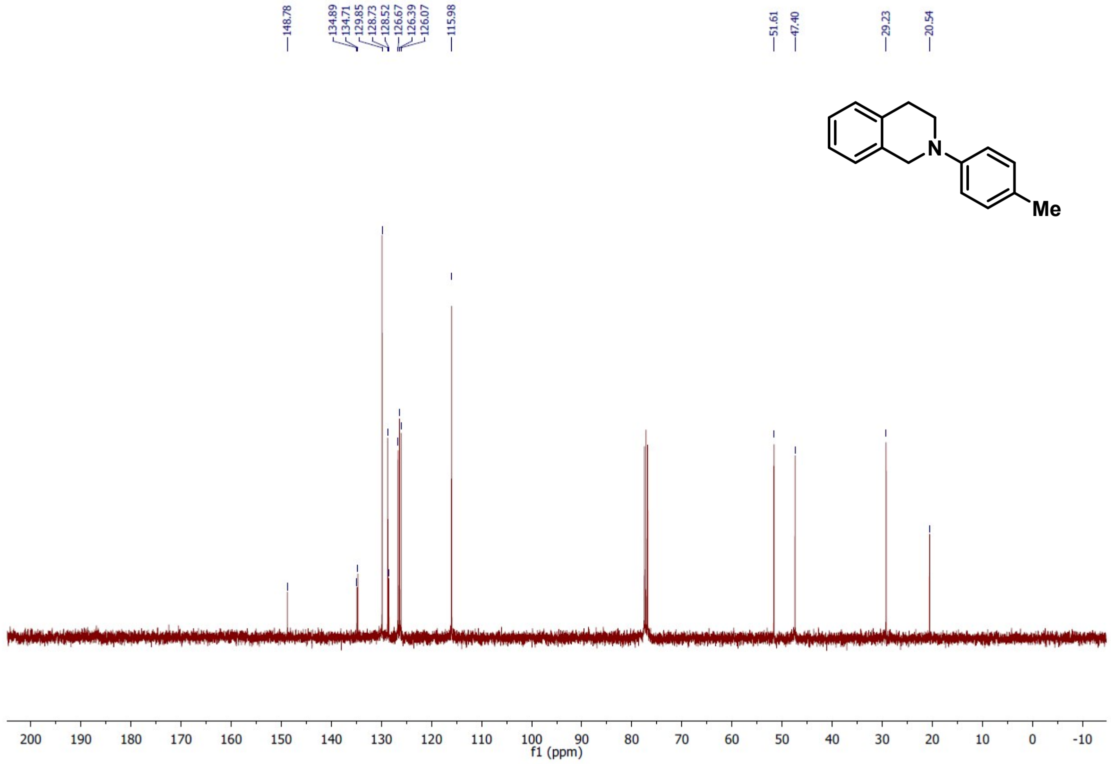

2-phenyl-1,2,3,4-tetrahydroisoquinoline (1c)

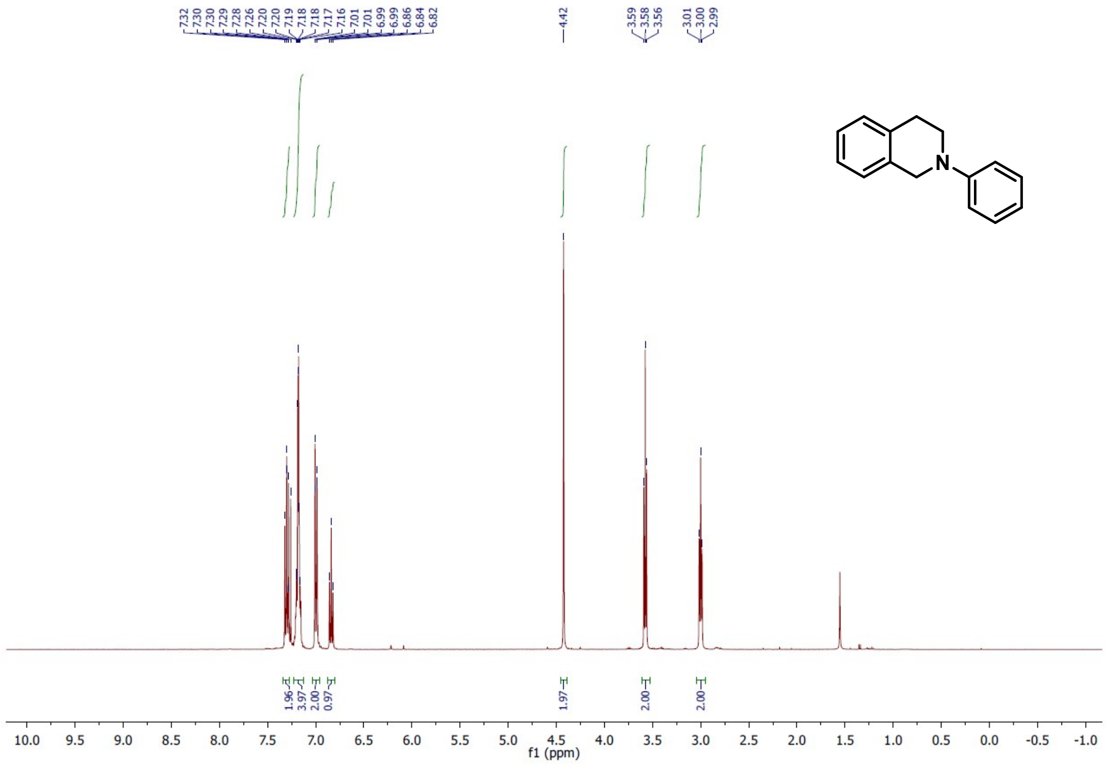

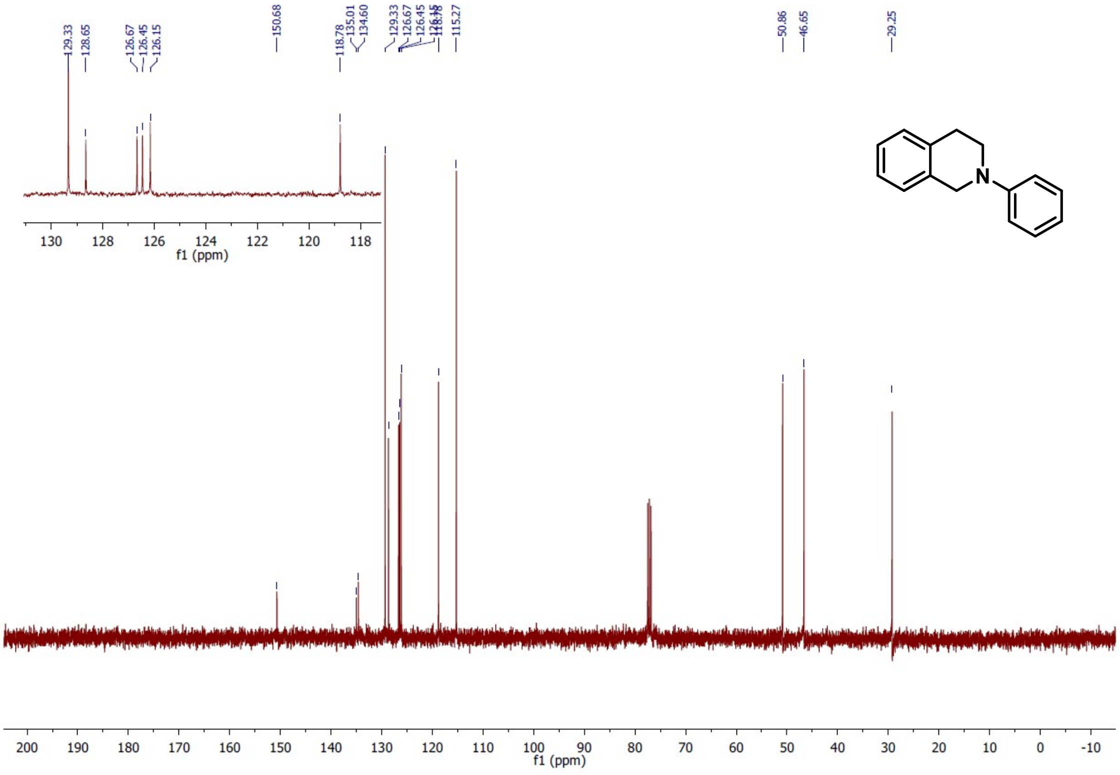

2-(4-fluorophenyl)-1,2,3,4-tetrahydroisoquinoline (1d)

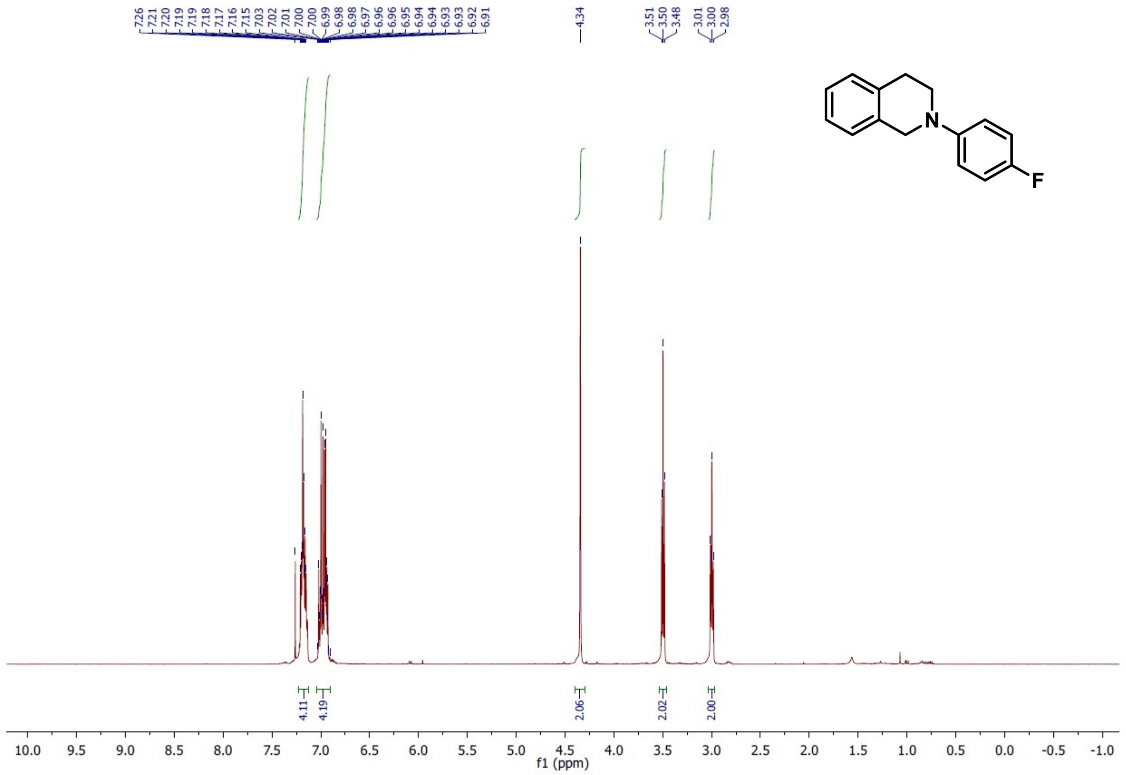

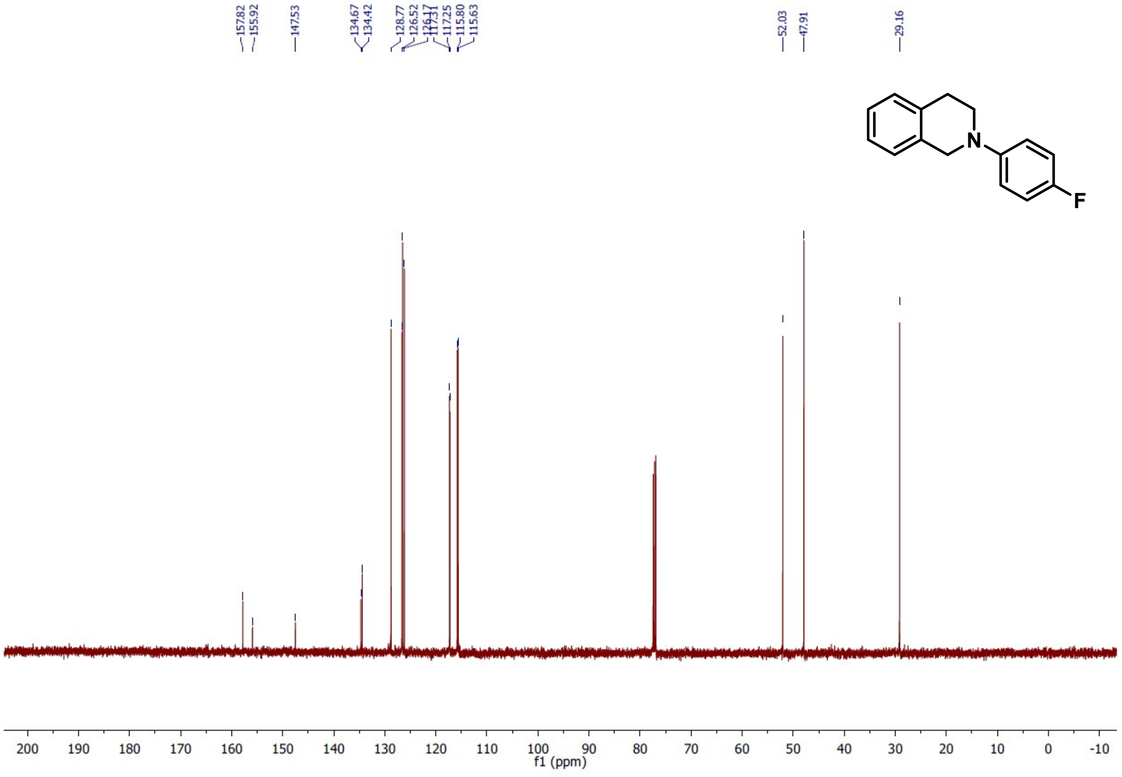

Ethyl 2-azidoacetate

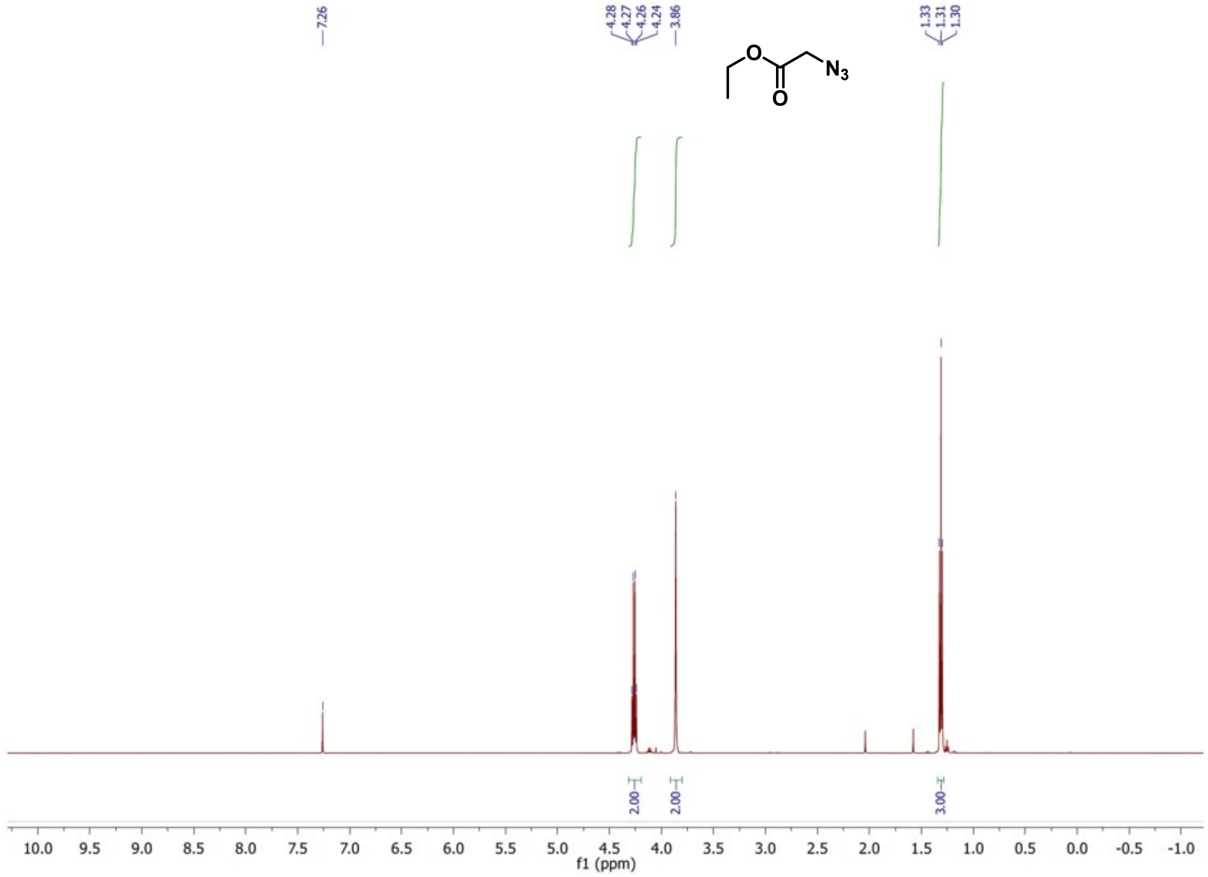

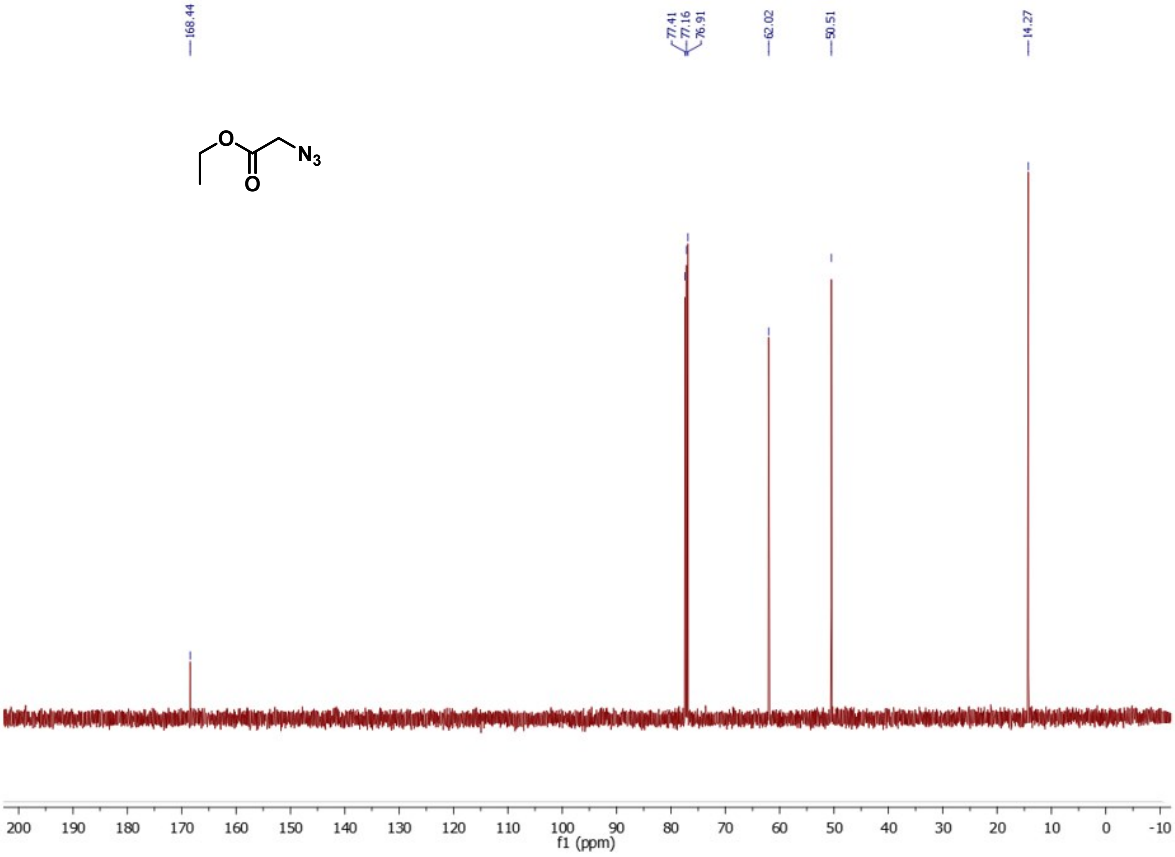

Ethyl (2E,4E)-2-methyl-5-phenylpenta-2,4-diene (14)

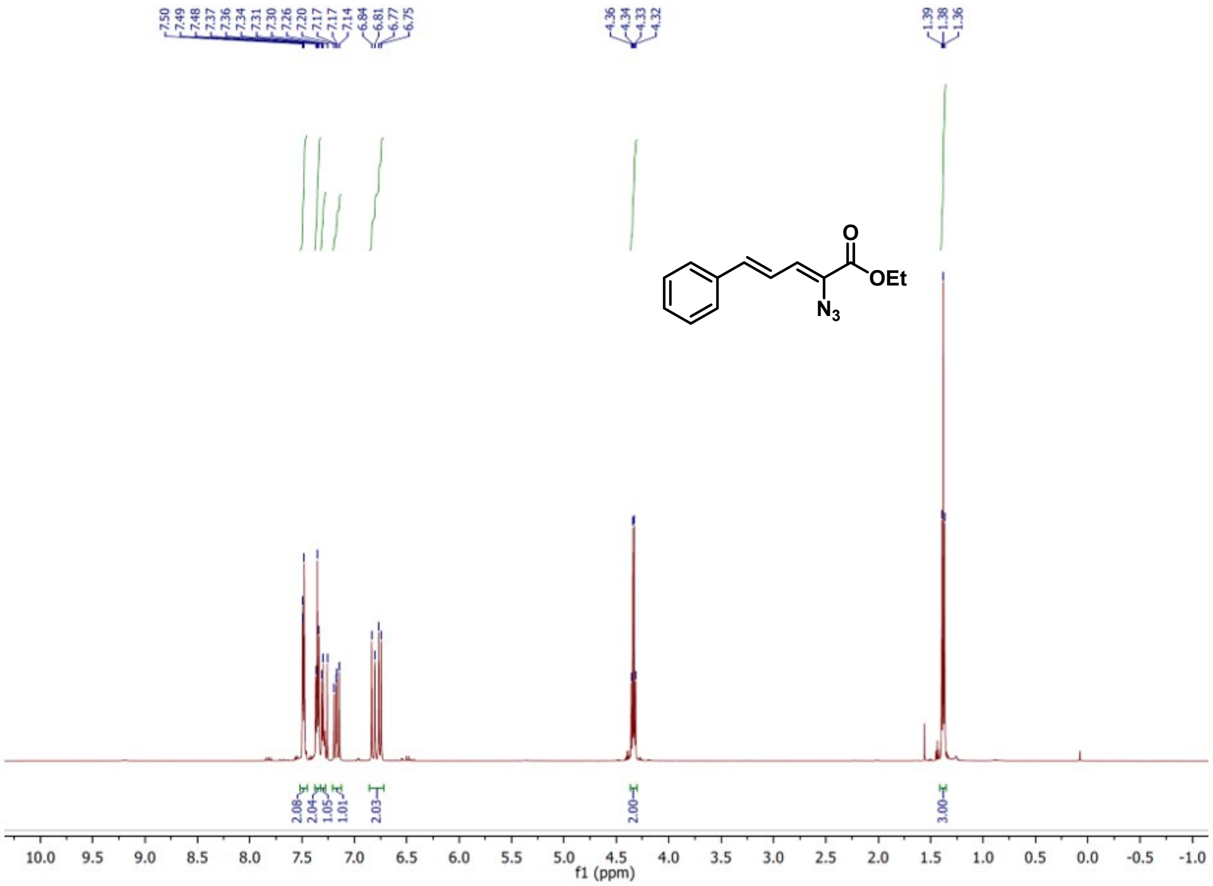

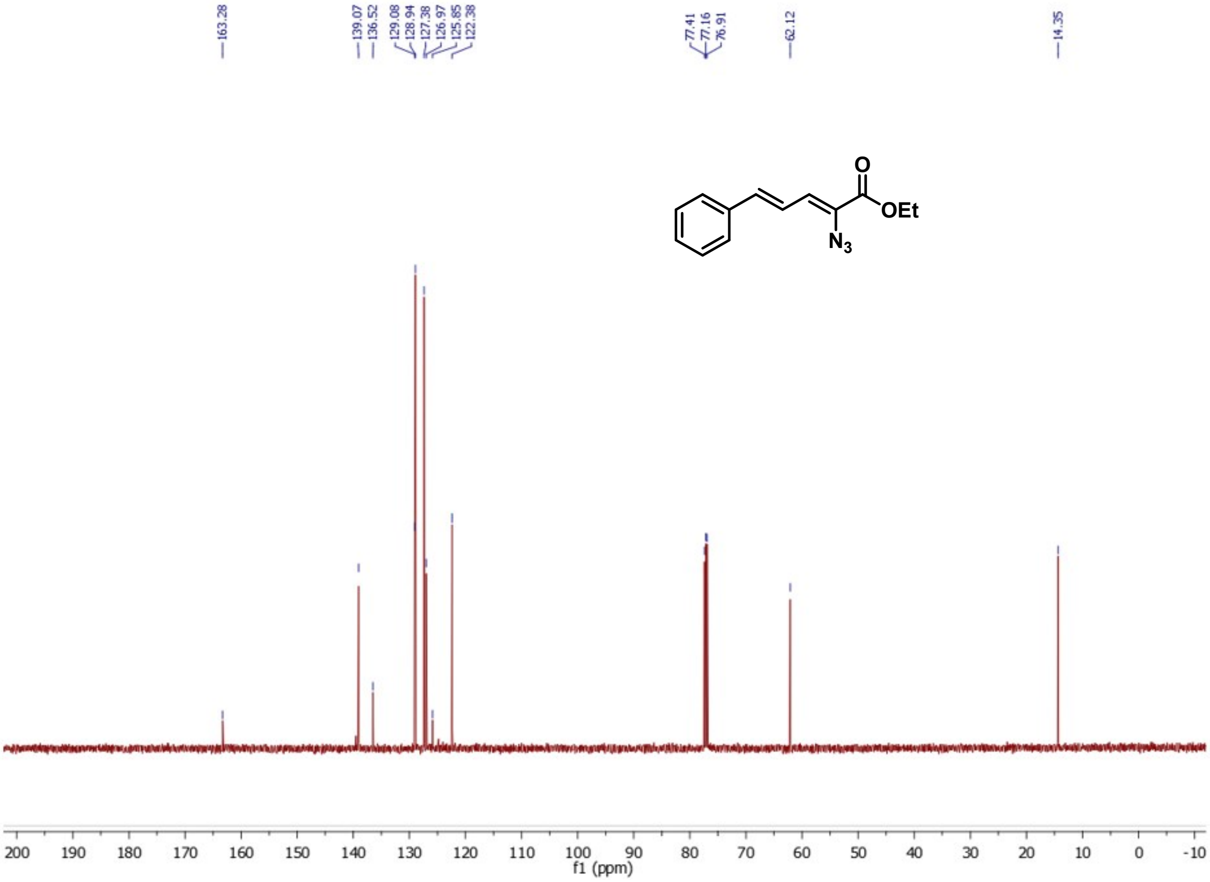

1,1-bis(4-methoxyphenyl)ethene (19)

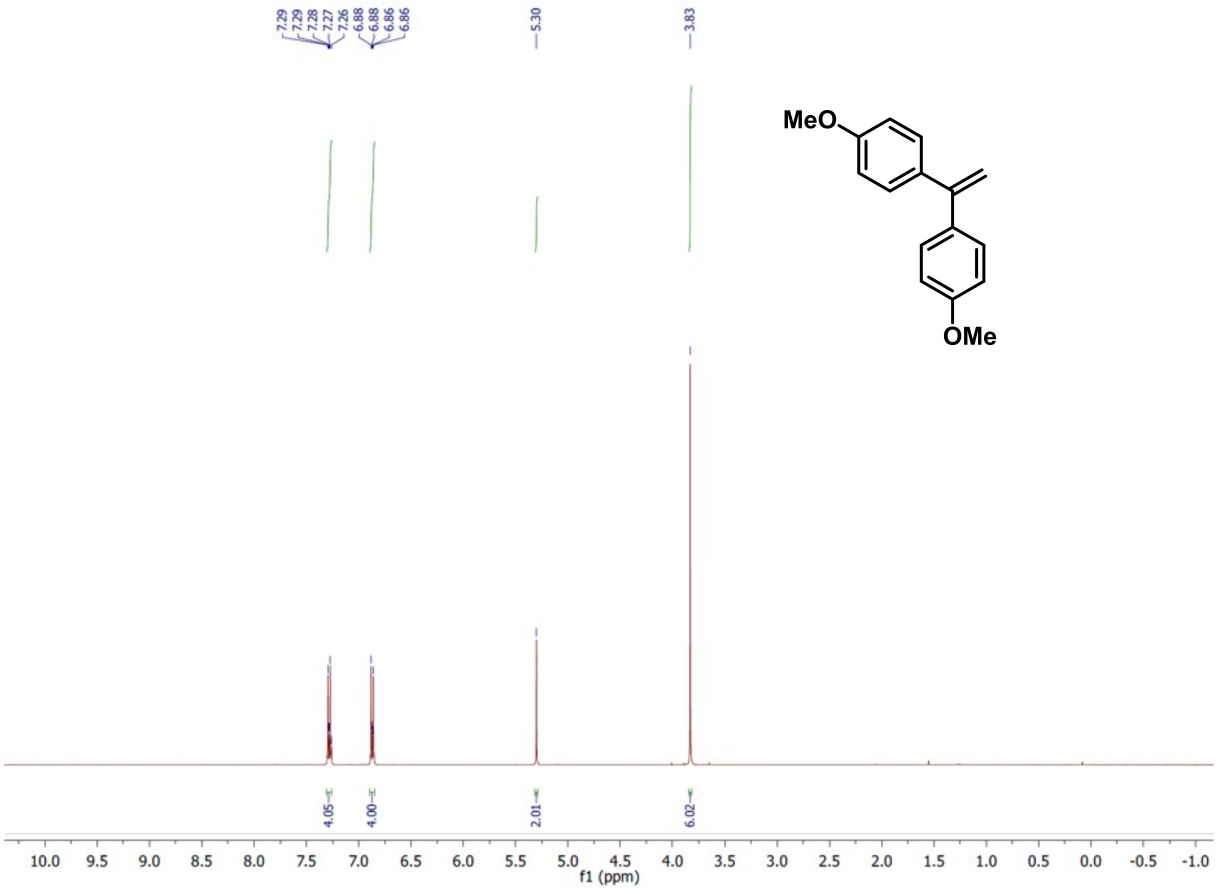

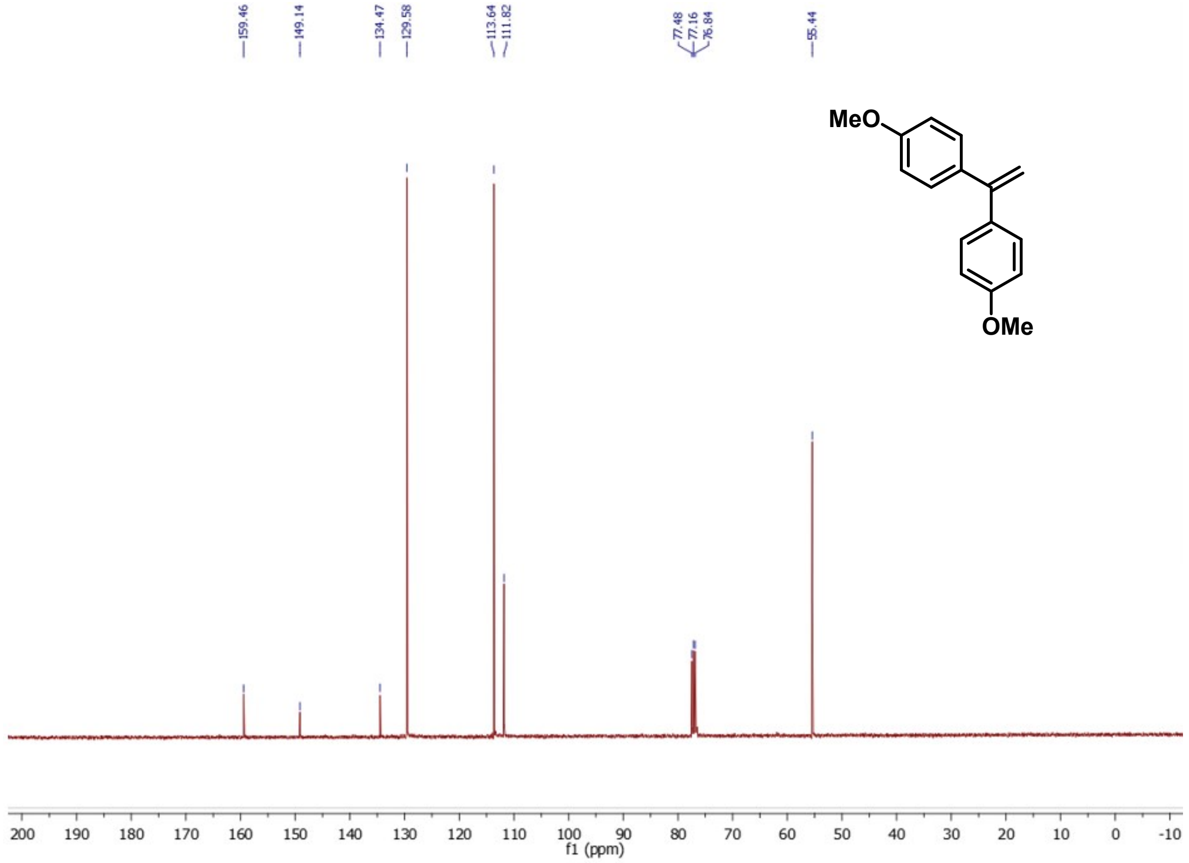

2-(4-methoxyphenyl)-1-(nitromethyl)-1,2,3,4-tetrahydroisoquinoline (2a)

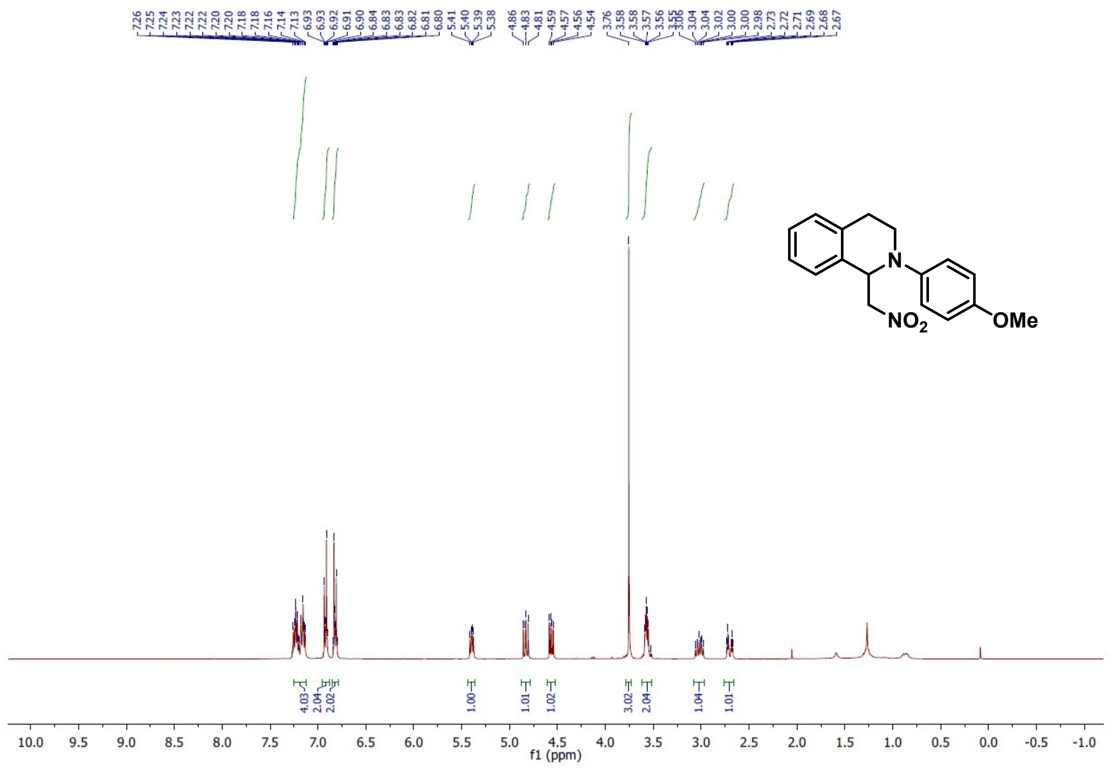

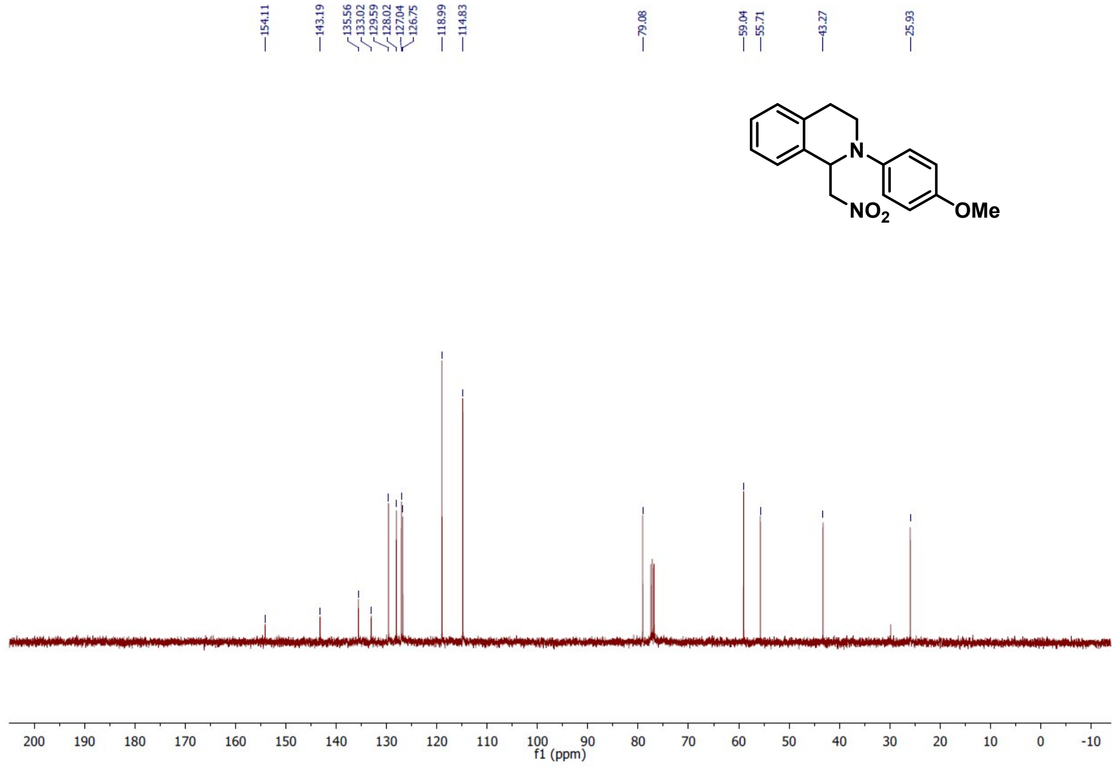

1-(nitromethyl)-2-(p-tolyl)-1,2,3,4-tetrahydroisoquinoline (2b)

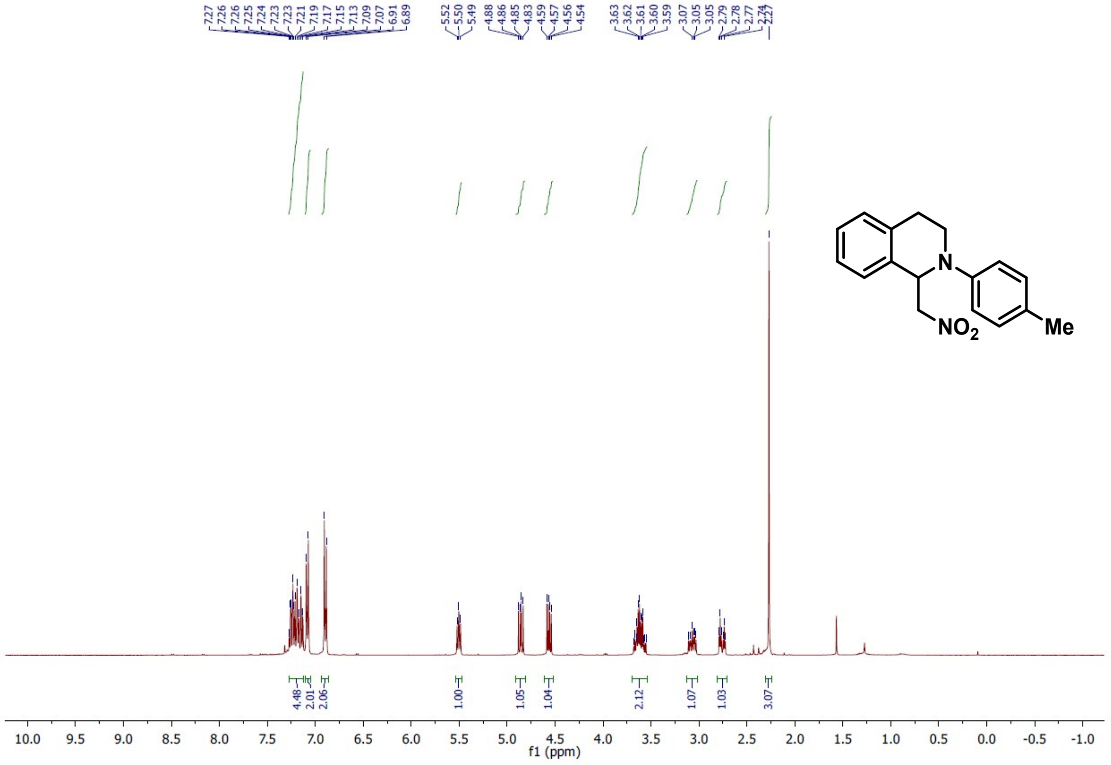

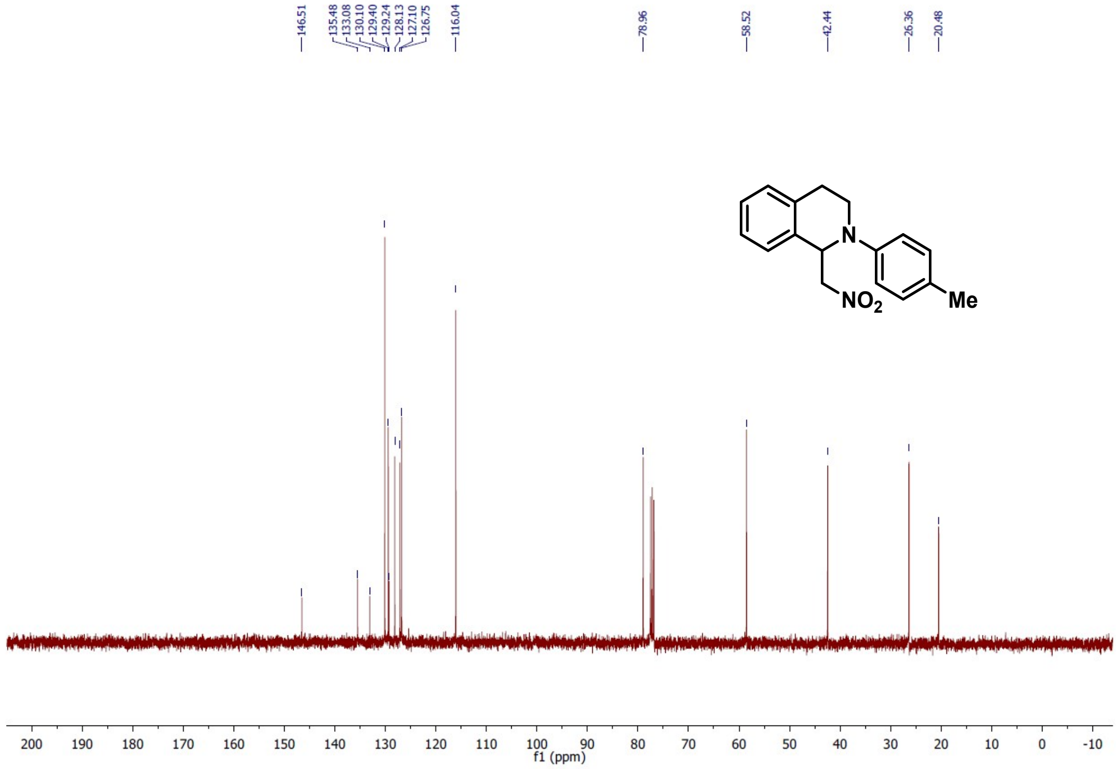

1-(nitromethyl)-2-phenyl-1,2,3,4-tetrahydroisoquinoline (2c)

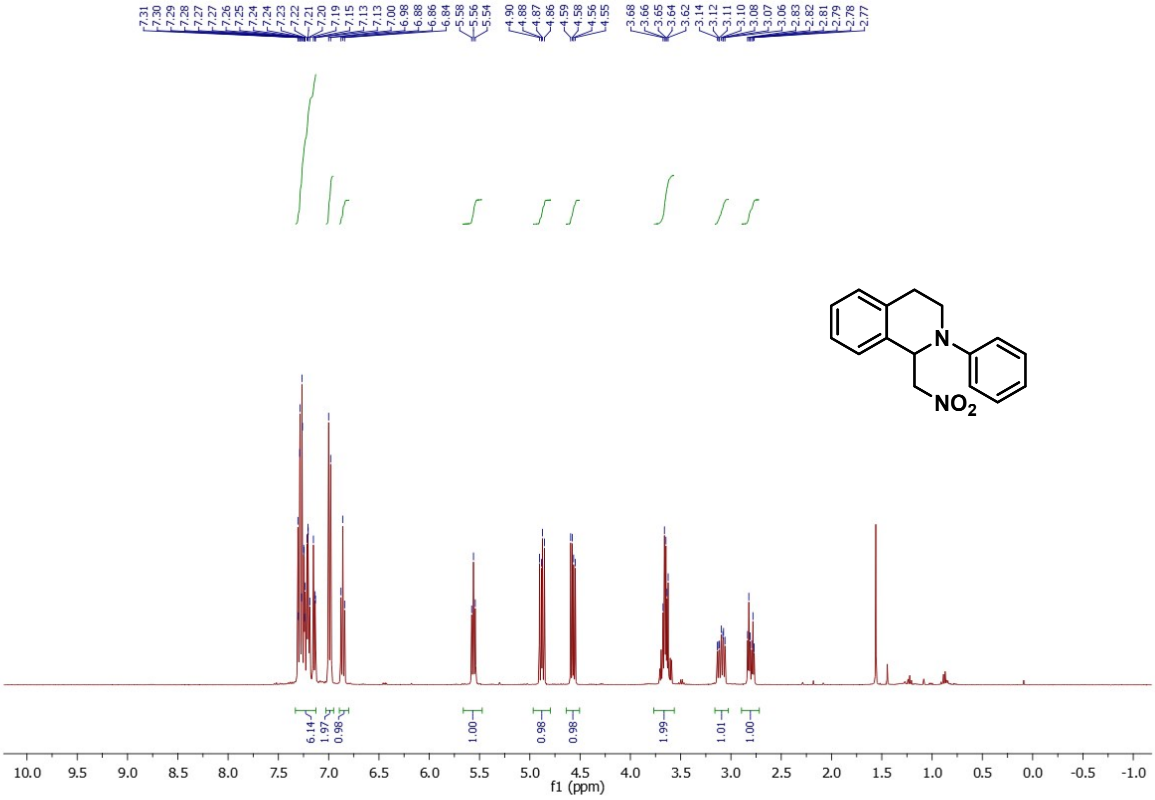

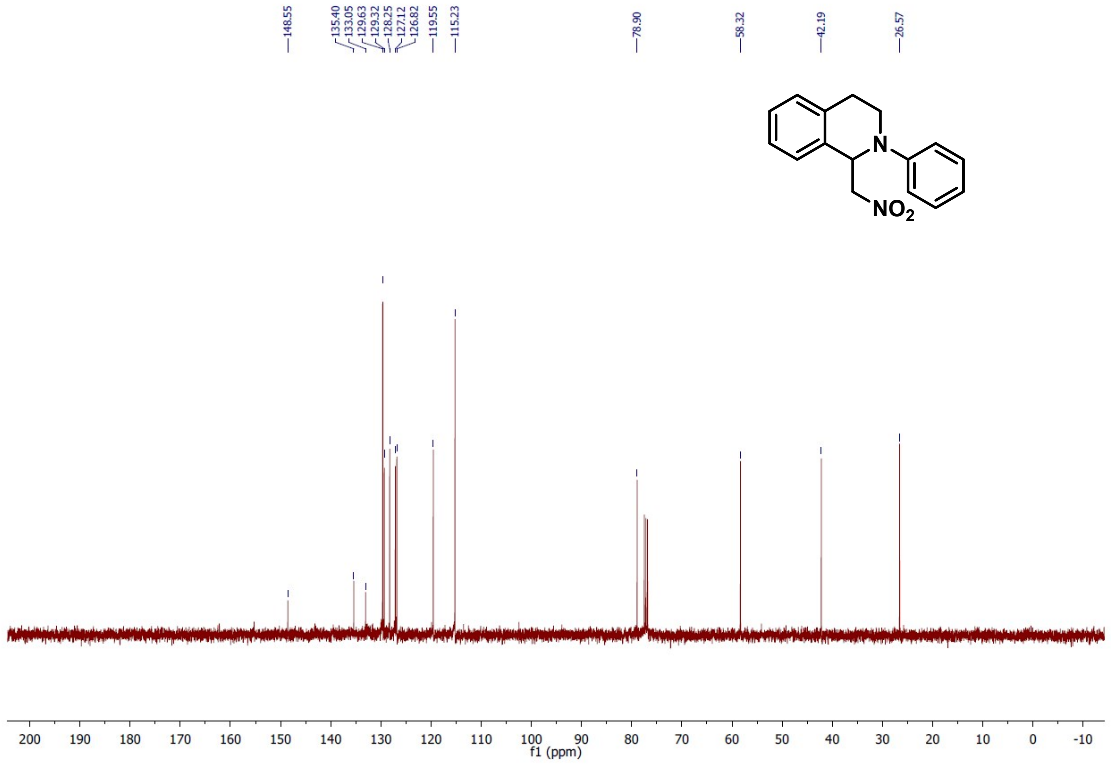

2-(4-fluorophenyl)-1-(nitromethyl)-1,2,3,4-tetrahydroisoquinoline (2d)

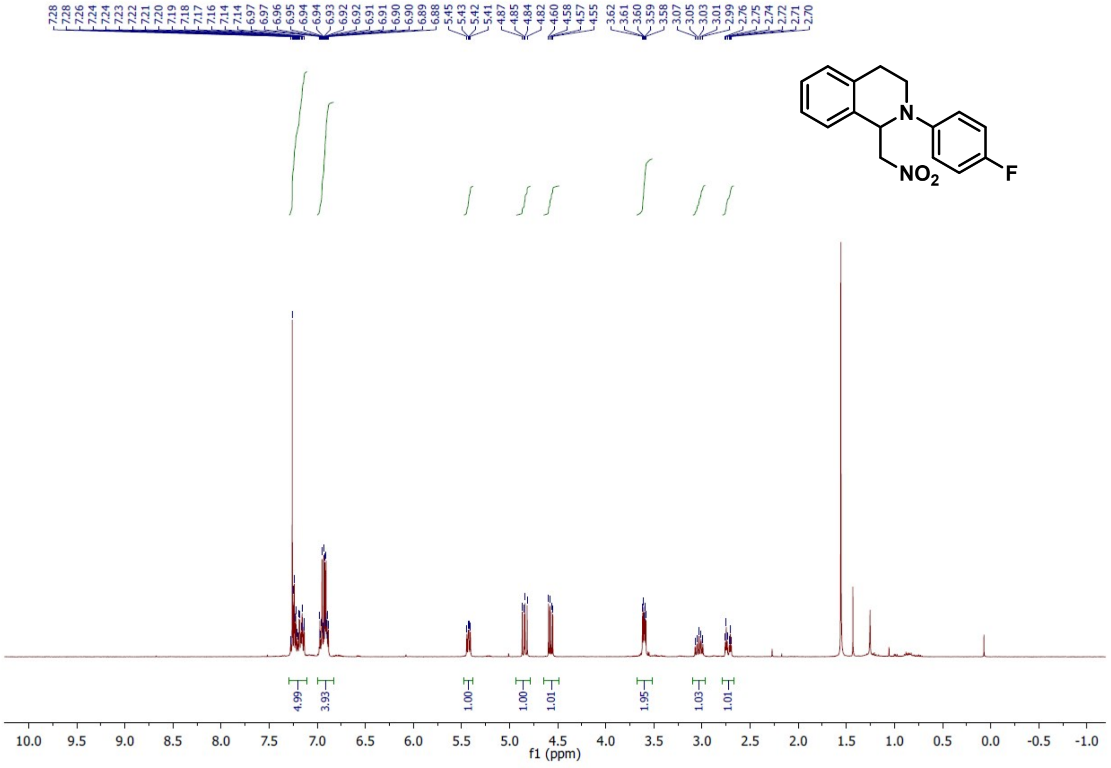

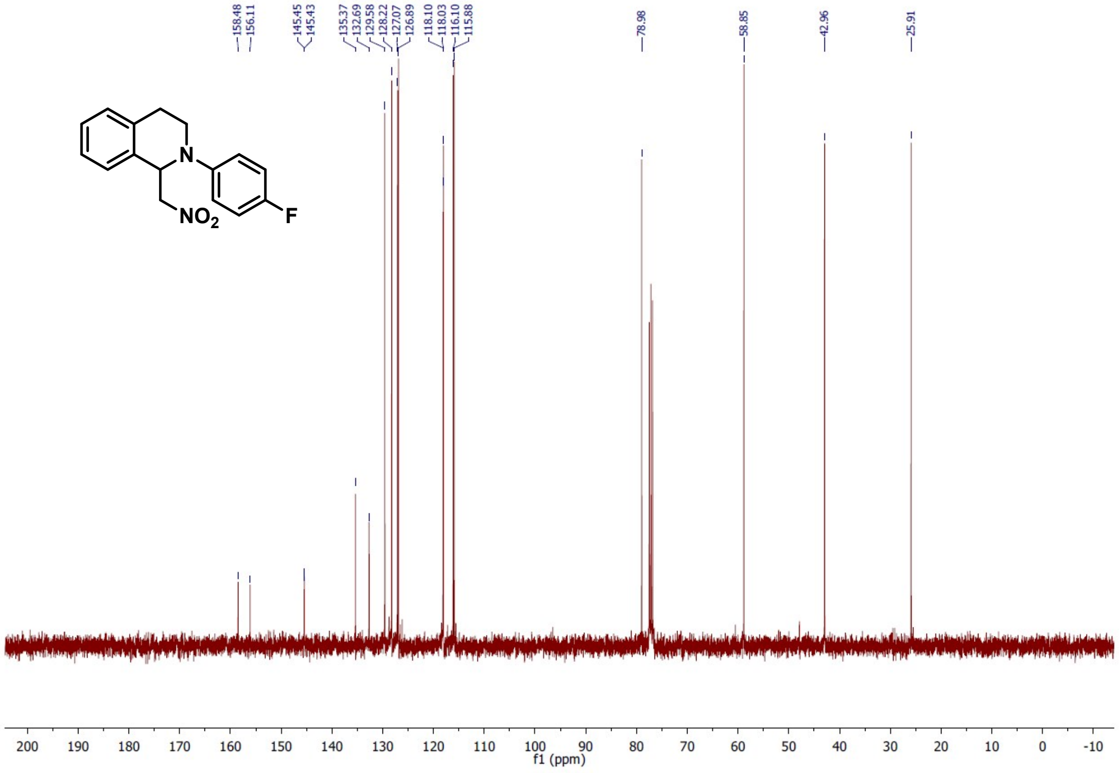

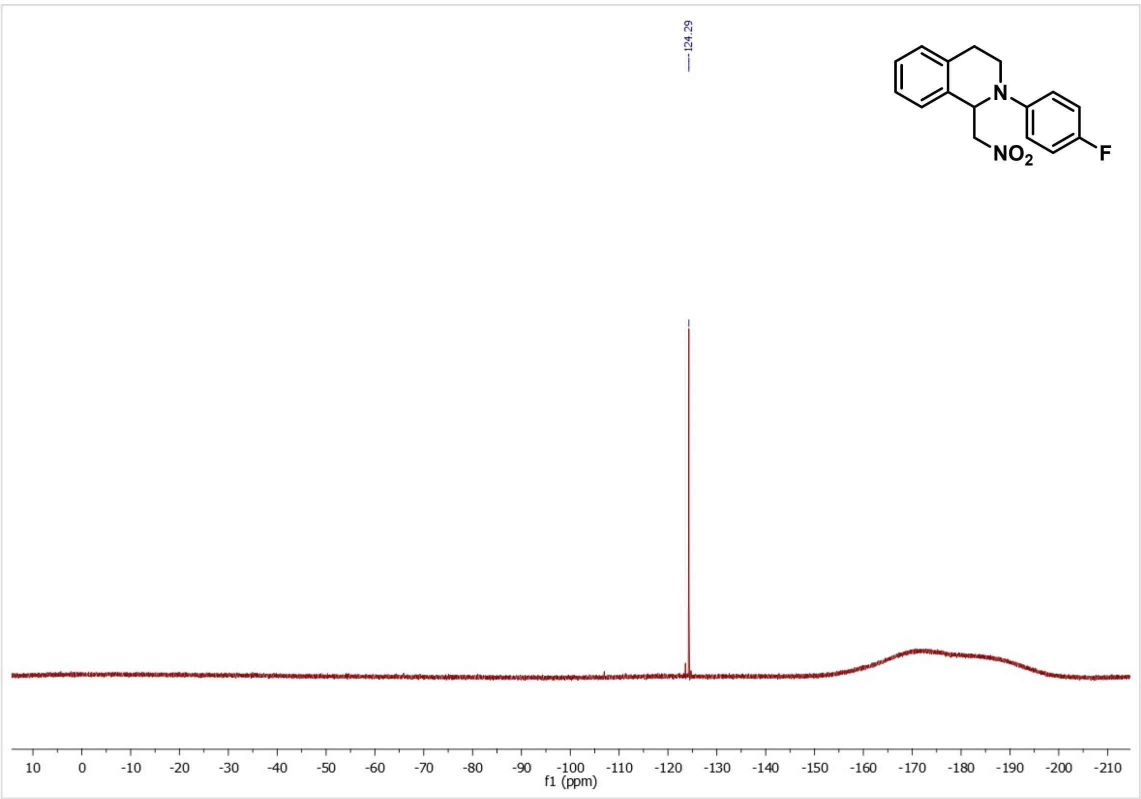

^{19}F NMR spectrum of 2d

1-(1-nitropropyl)-2-phenyl-1,2,3,4-tetrahydroisoquinoline (2e)

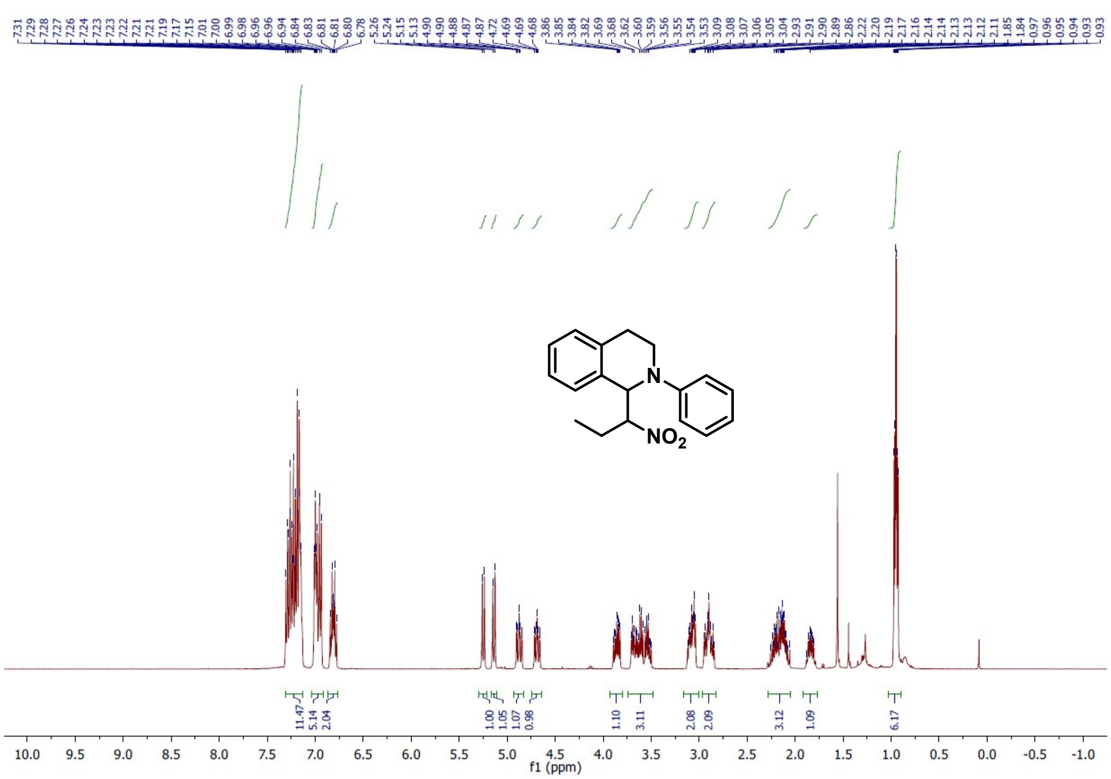

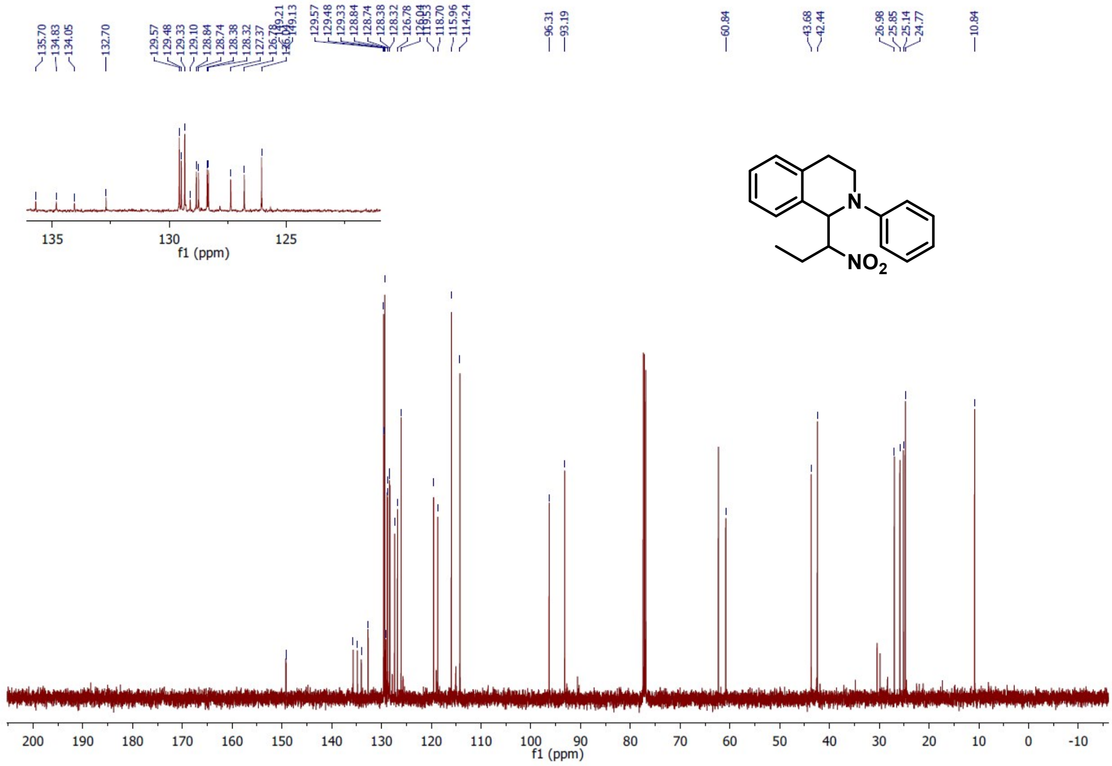

Dimethyl-2-(2-phenyl-1,2,3,4-tetrahydroisoquinolin-1-yl)malonate (2f)

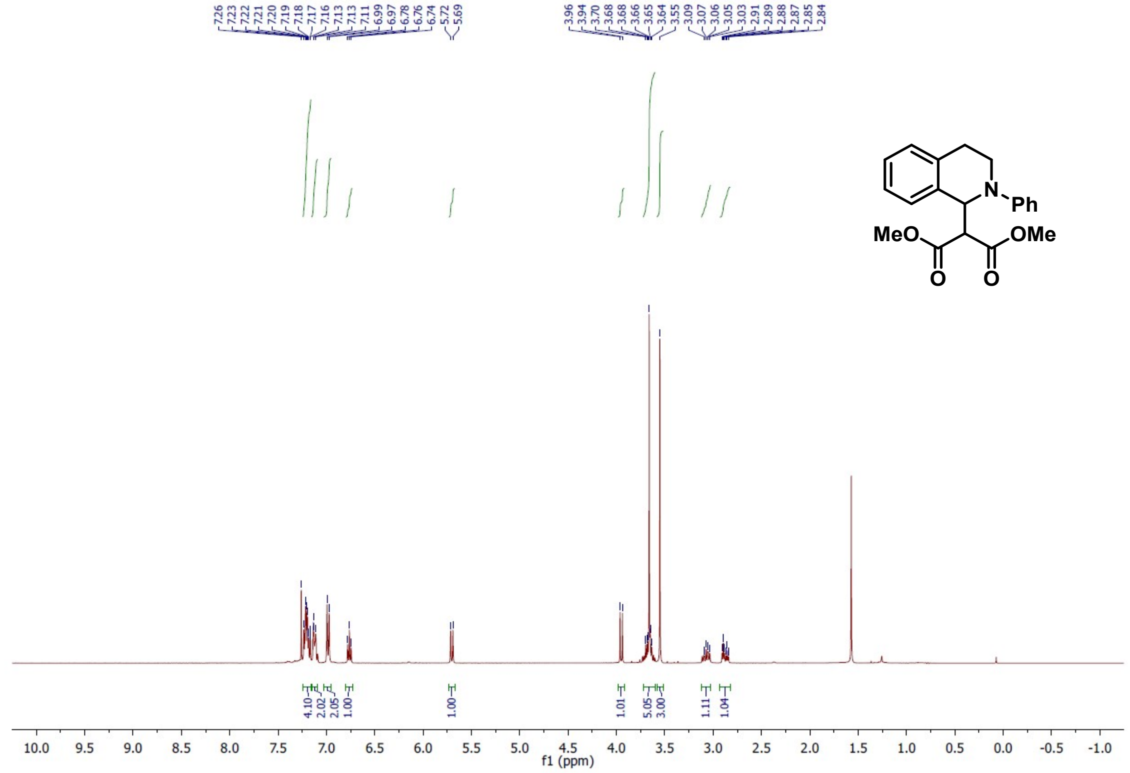

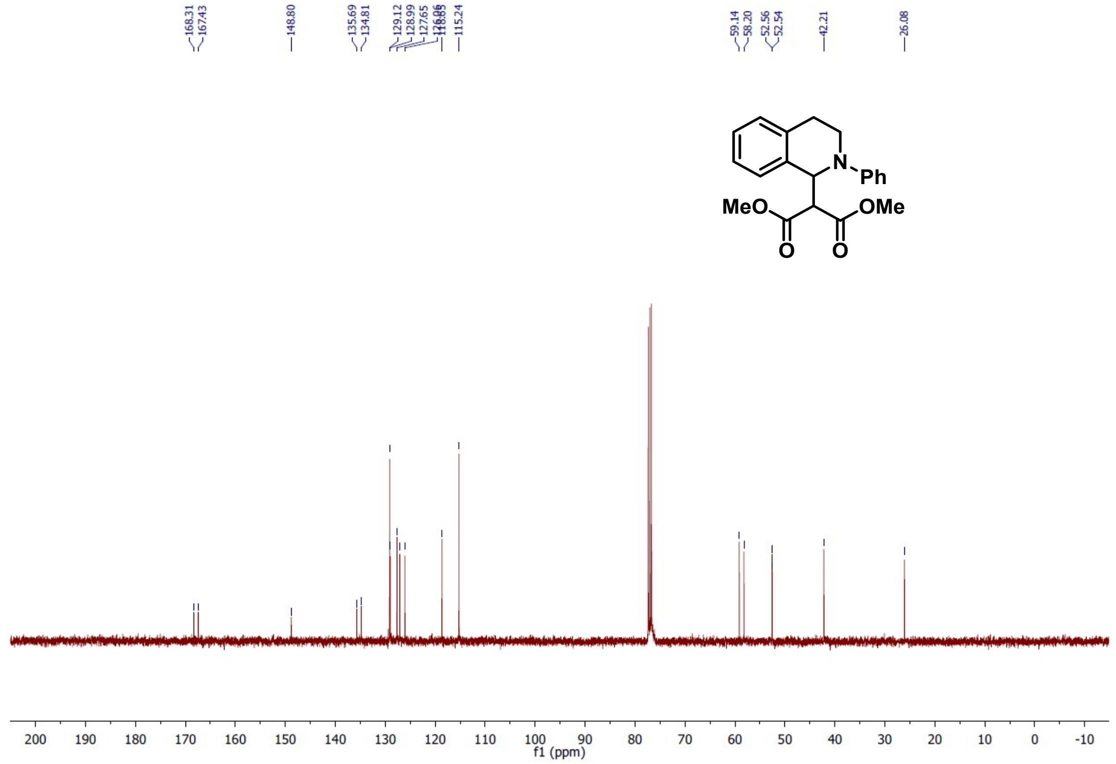

2-(4-methoxyphenyl)-1,2,3,4-tetrahydroisoquinoline-1-carbonitrile (2g)

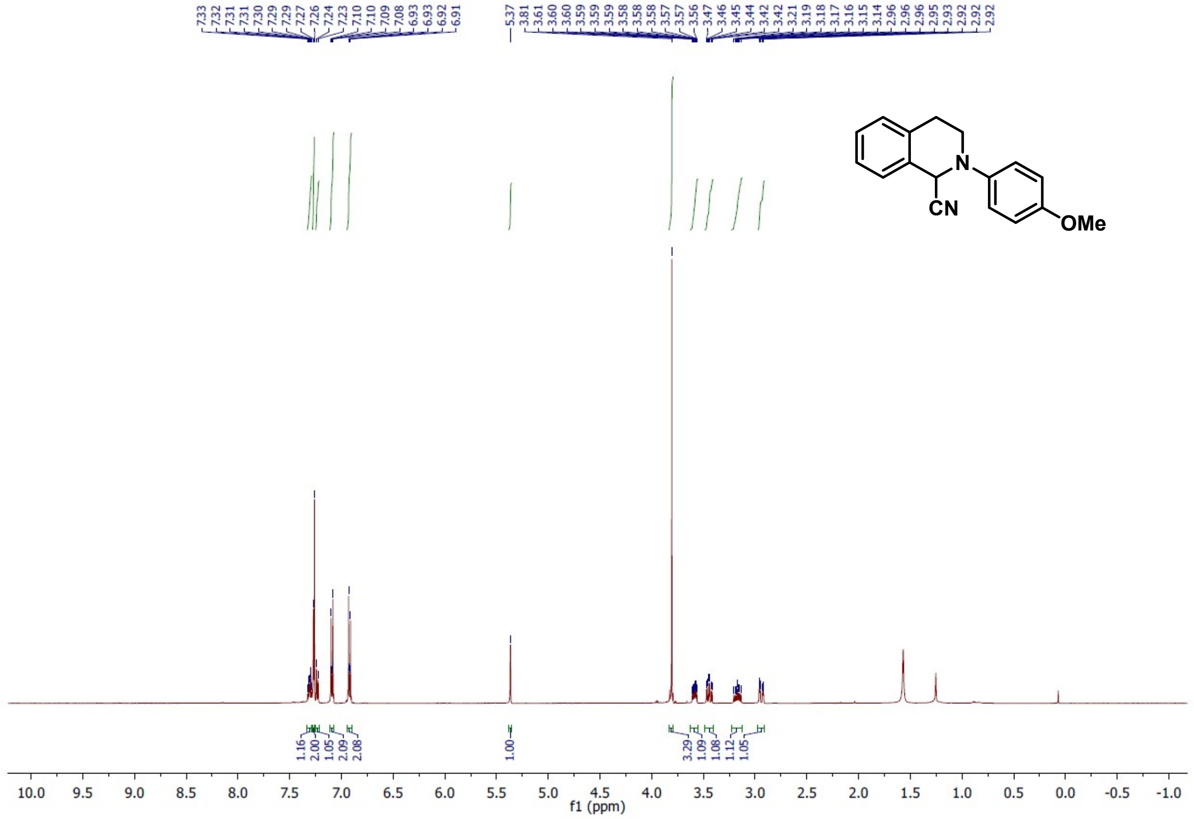

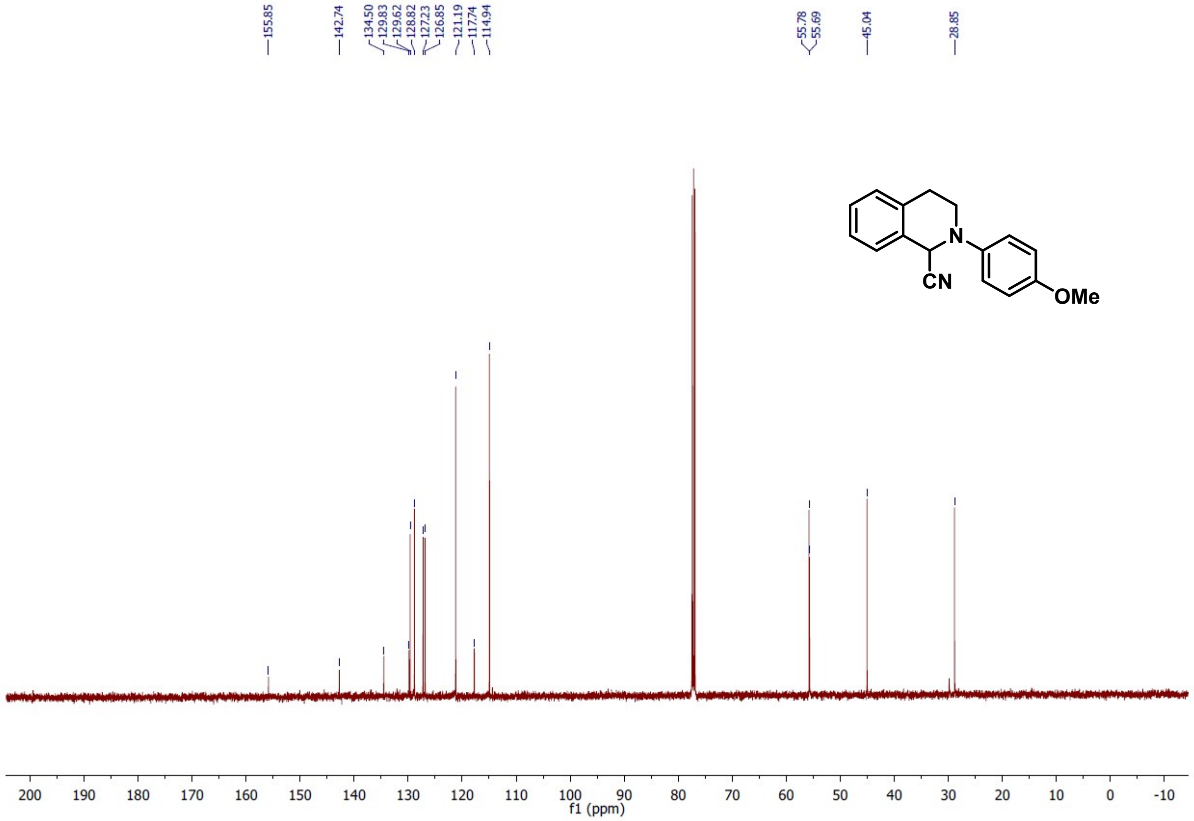

Diethyl (2-(4-methoxyphenyl)-1,2,3,4-tetrahydroisoquinolin-1-yl)phosphonate (2h)

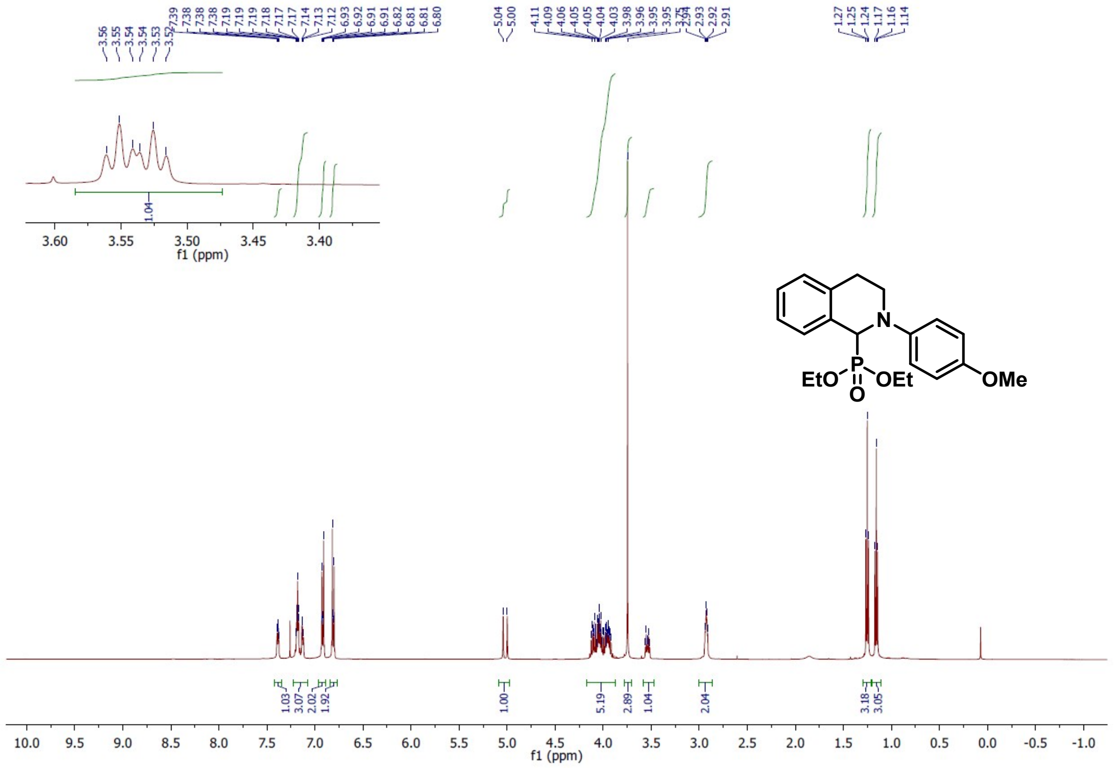

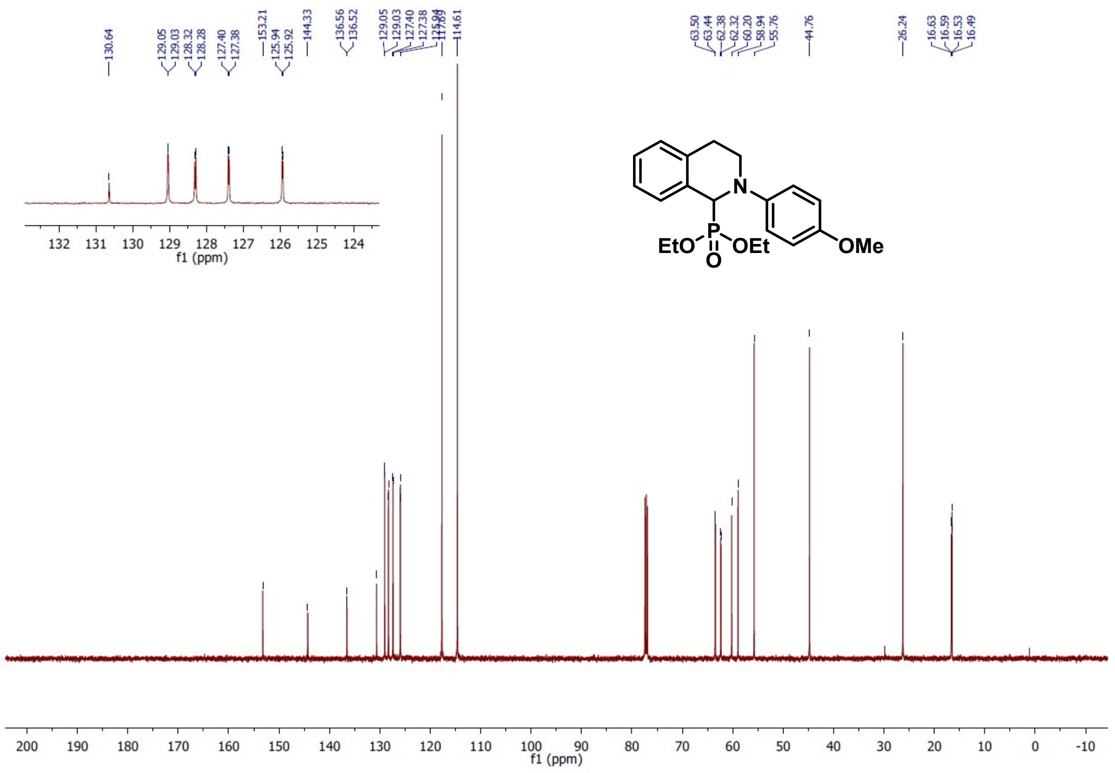

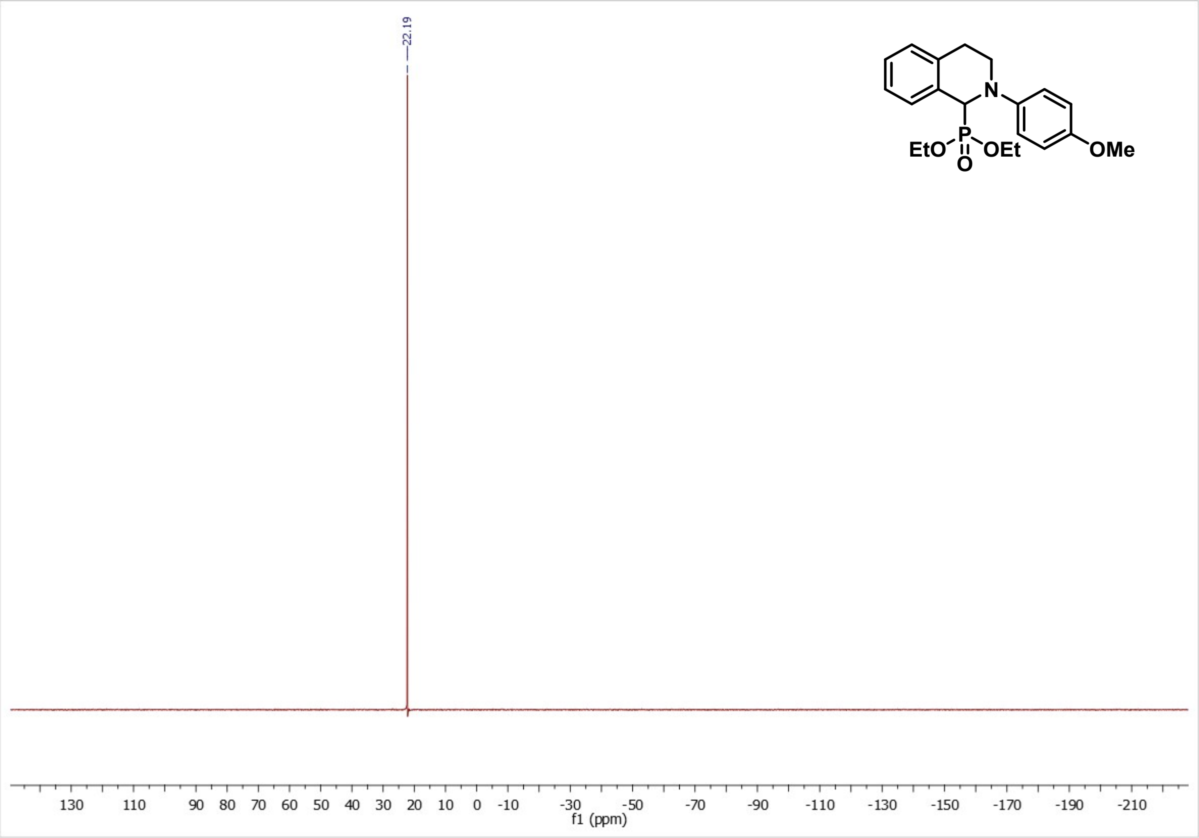

^{31}P NMR spectrum of 2h

2-(4-methoxyphenyl)-1-(phenylethynyl)-1,2,3,4-tetrahydroisoquinoline (2i):

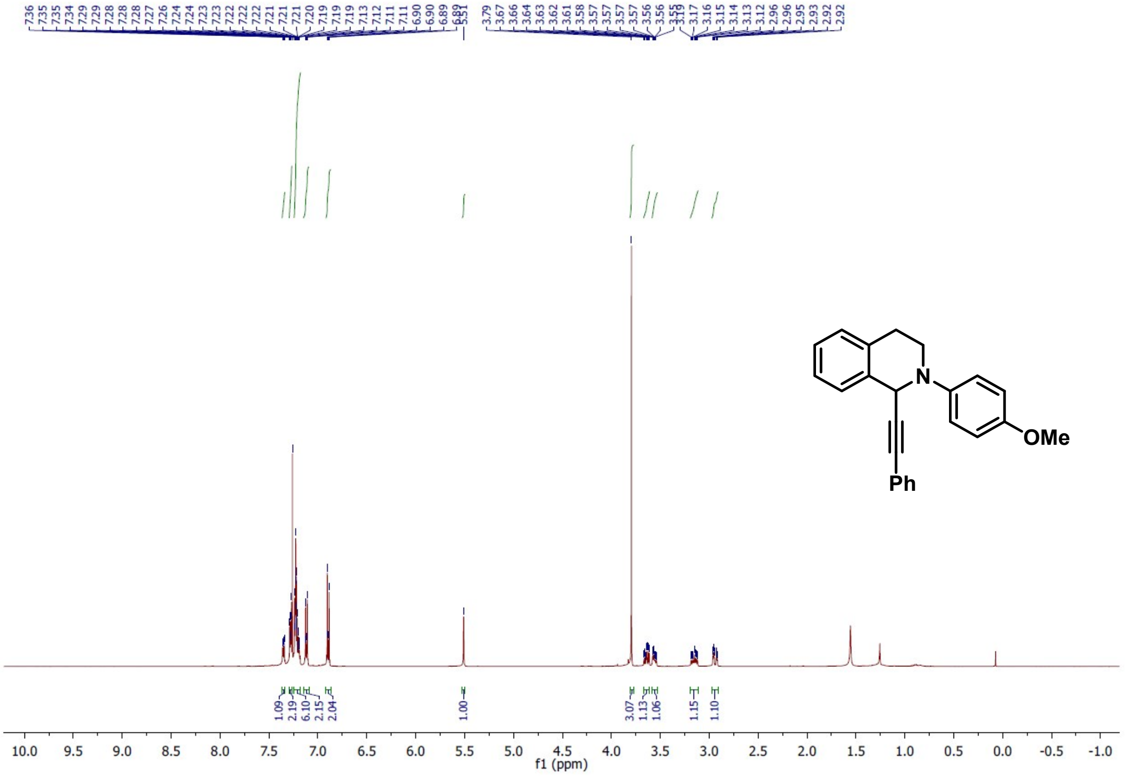

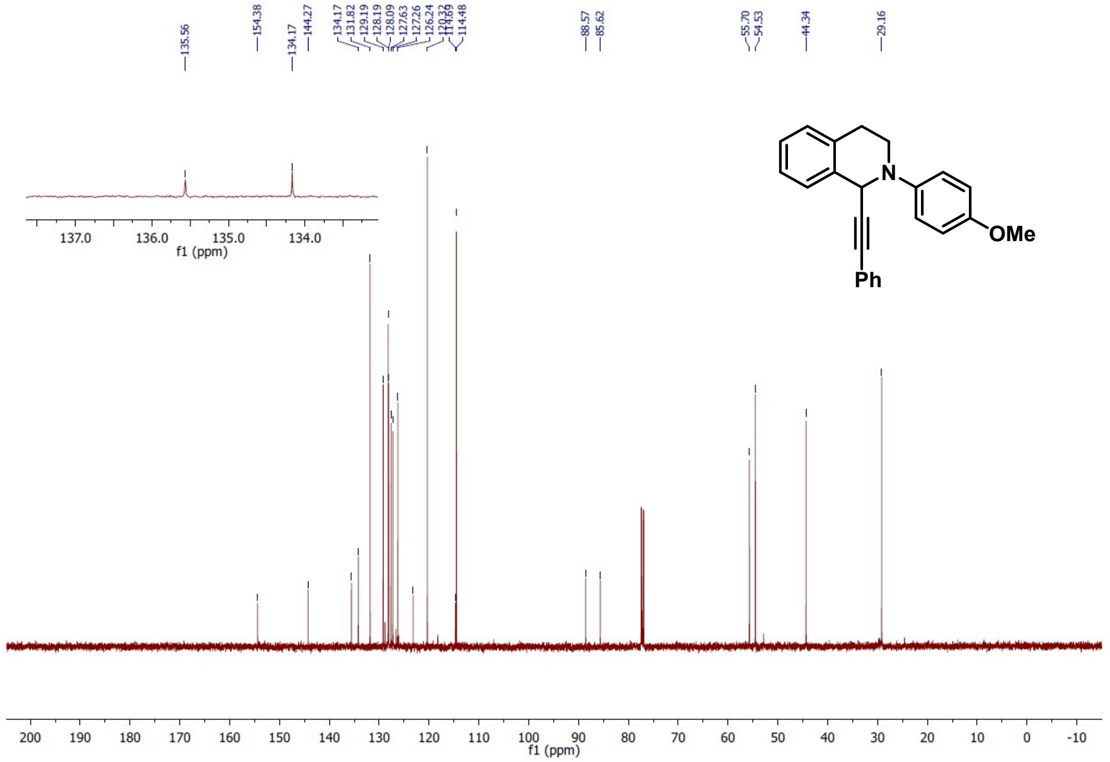

(3aS,9bR)-5-methyl-2-phenyl-3a,4,5,9b-tetrahydro-1H-pyrrolo[3,4-c]quinoline-1,3(2H)-dione (5)

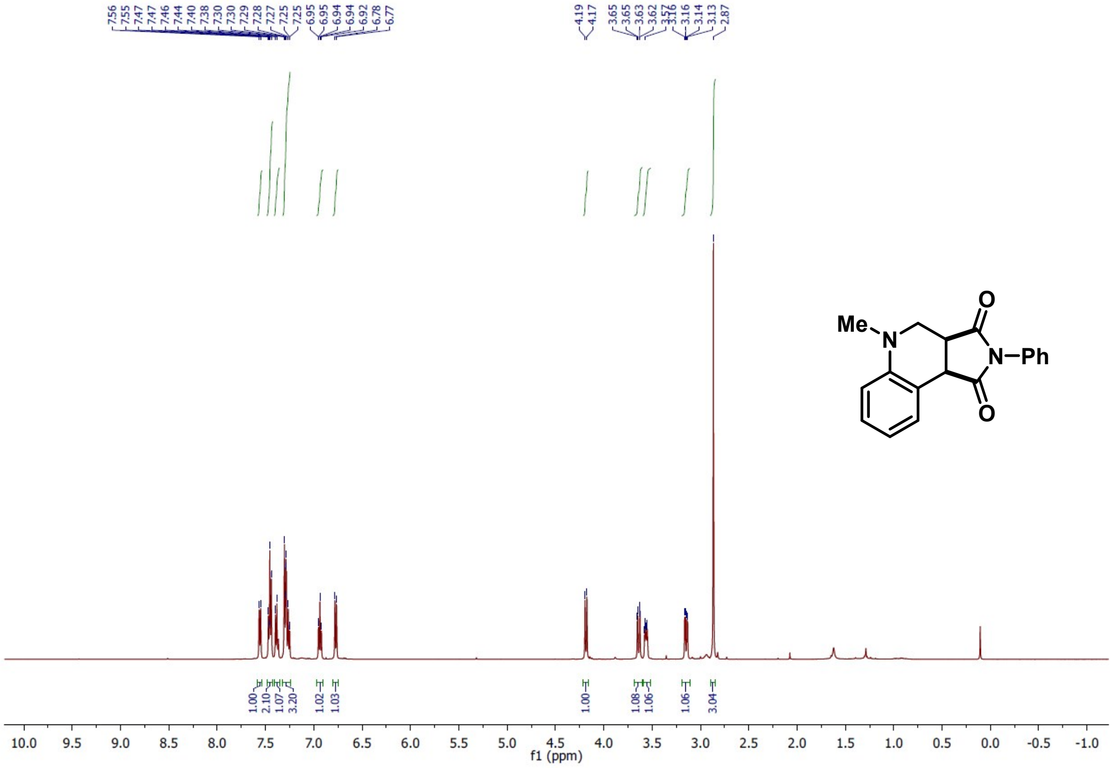

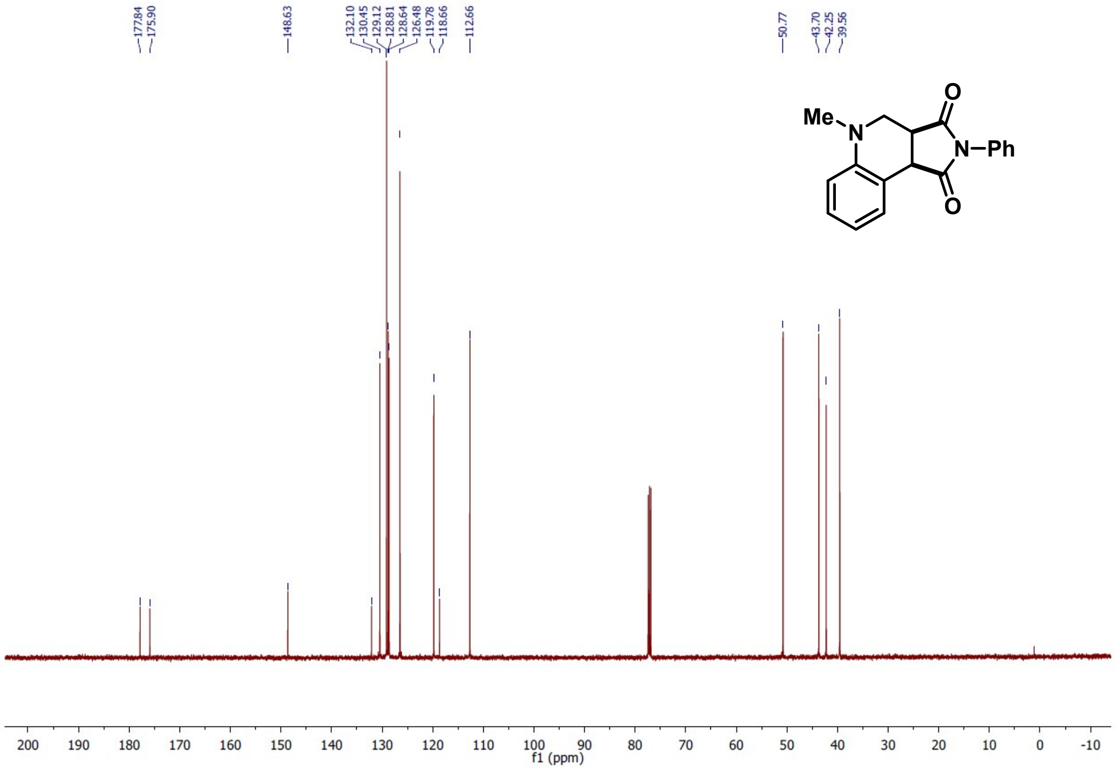

3,5-diphenyl-1,2,4-thiadiazole (7)

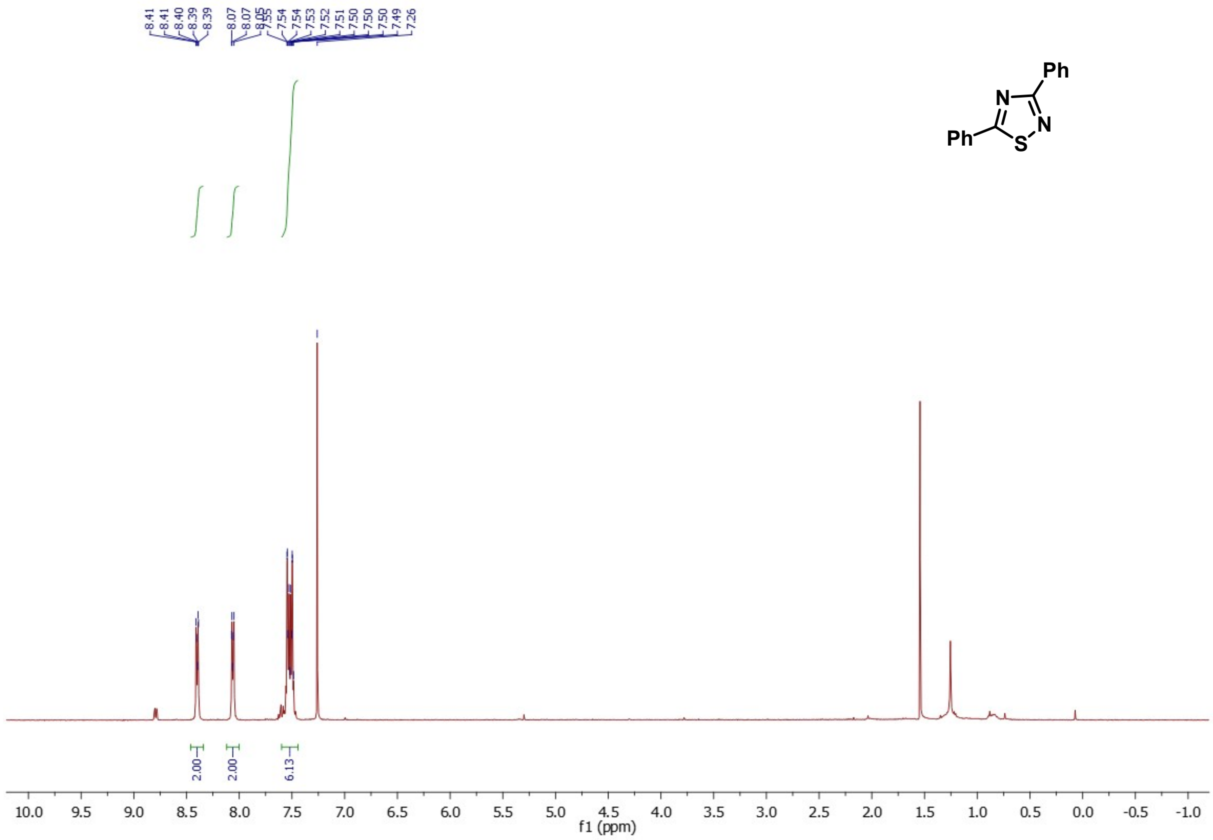

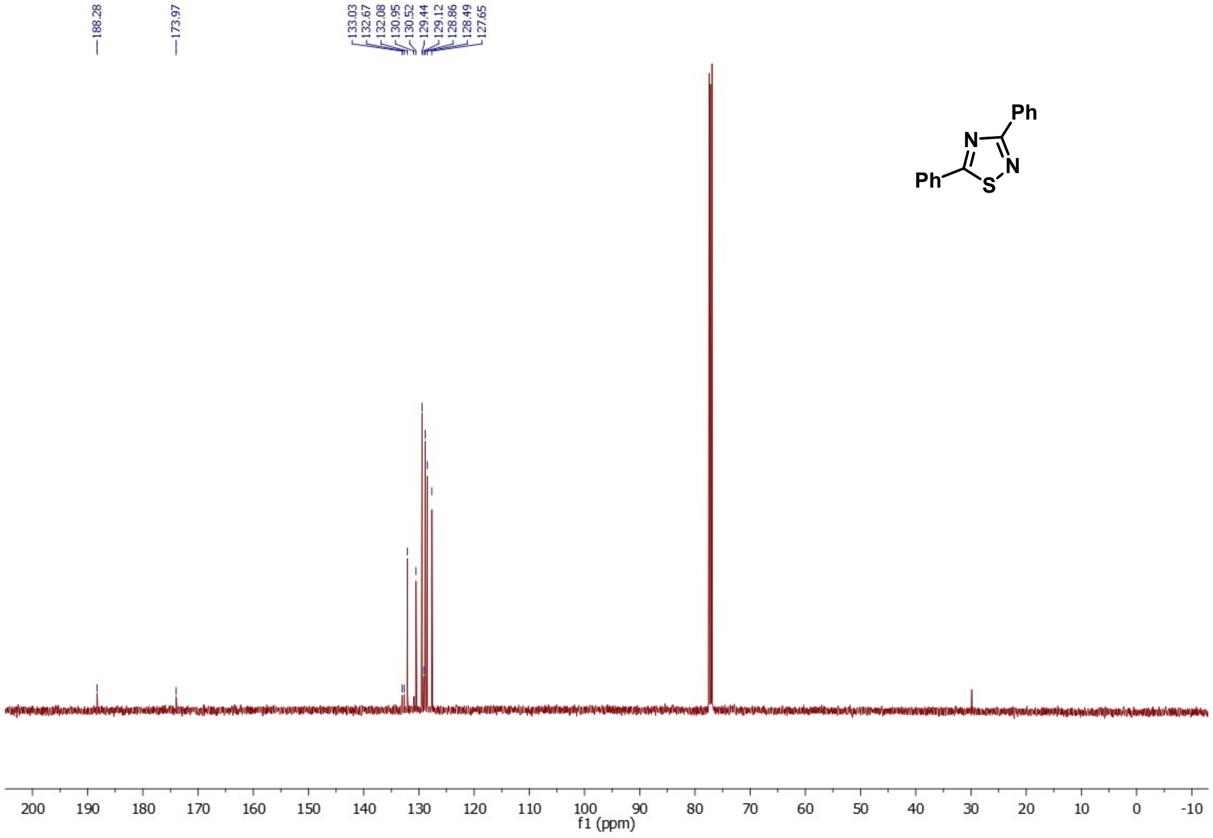

4-methoxyphenoln(9):

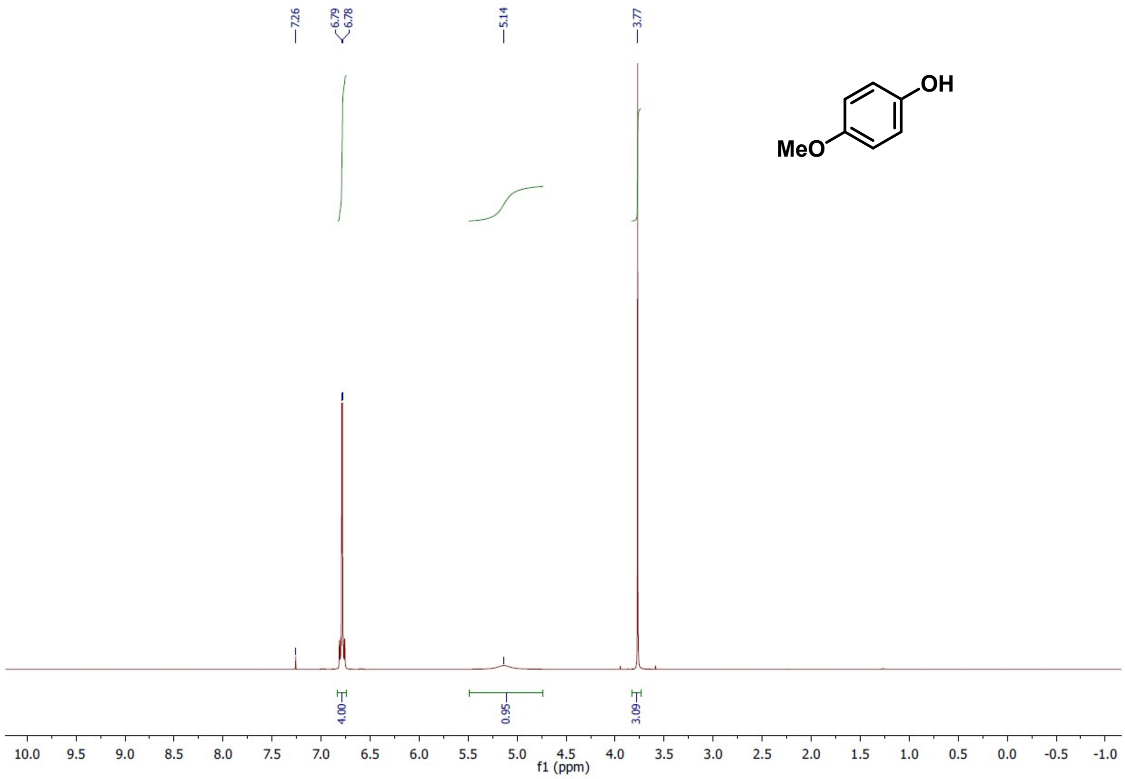

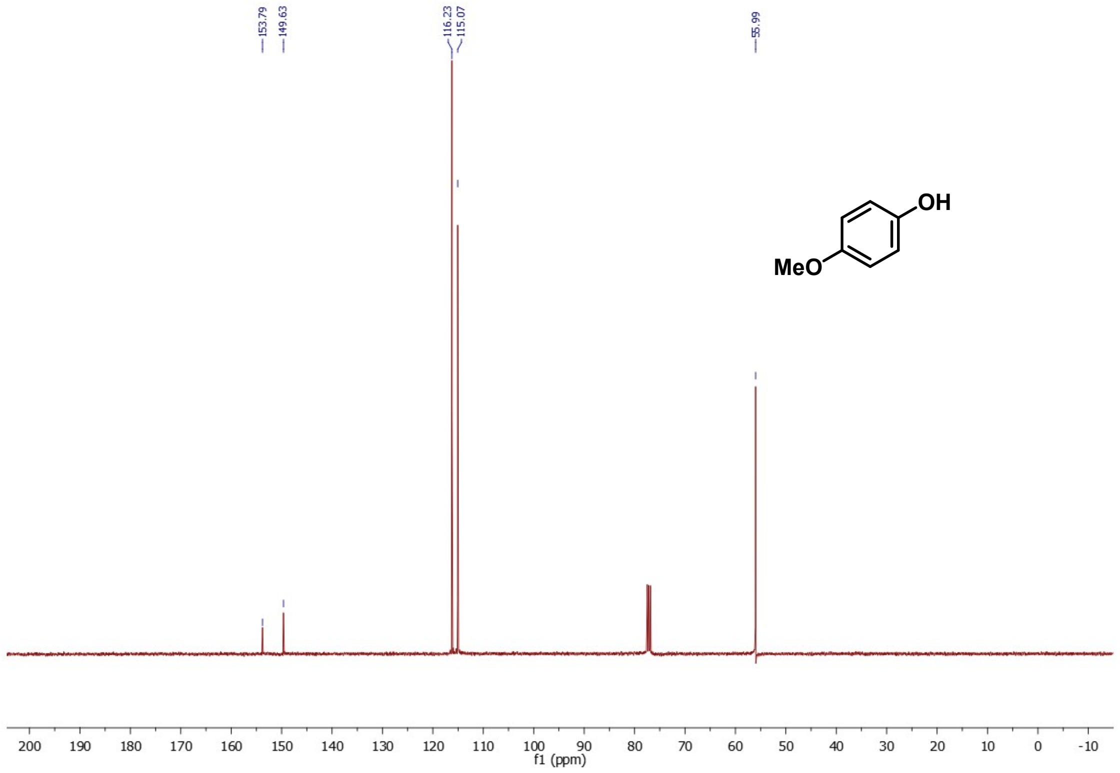

5-hydroxyfuran-2(5H)-one (11):

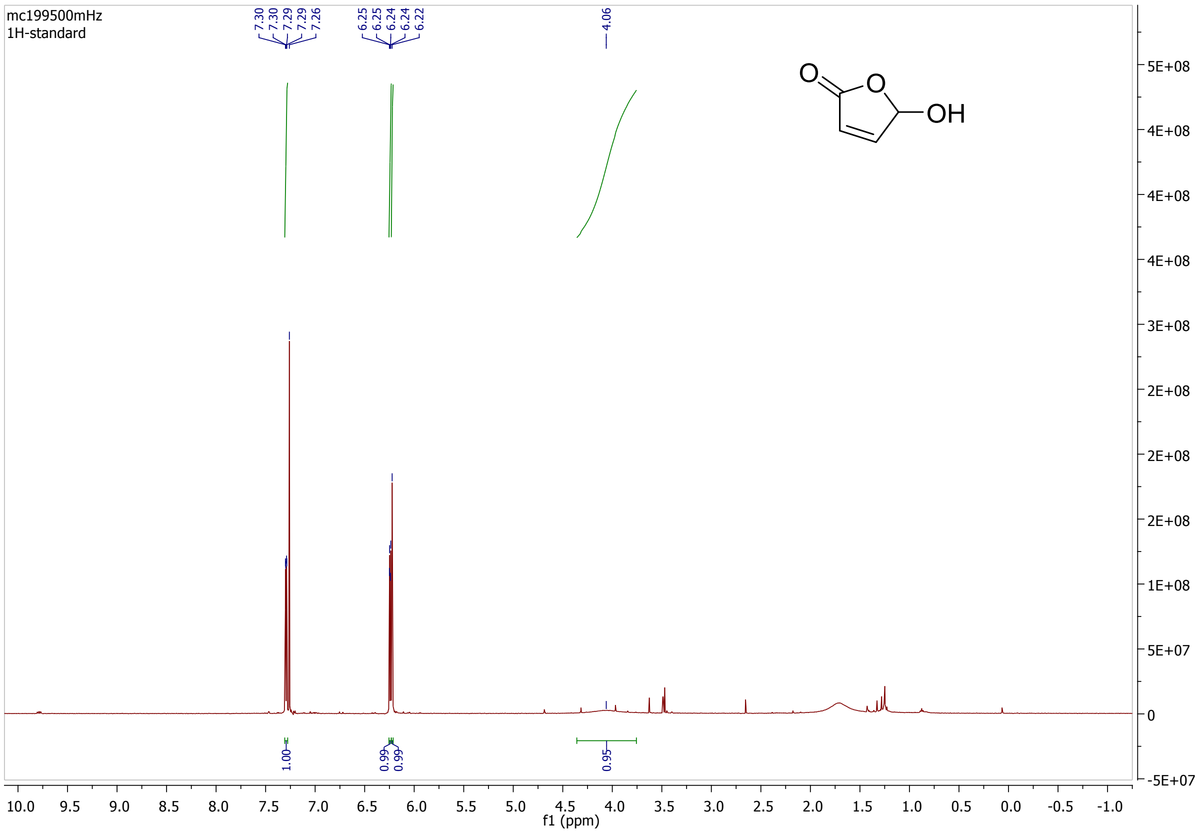

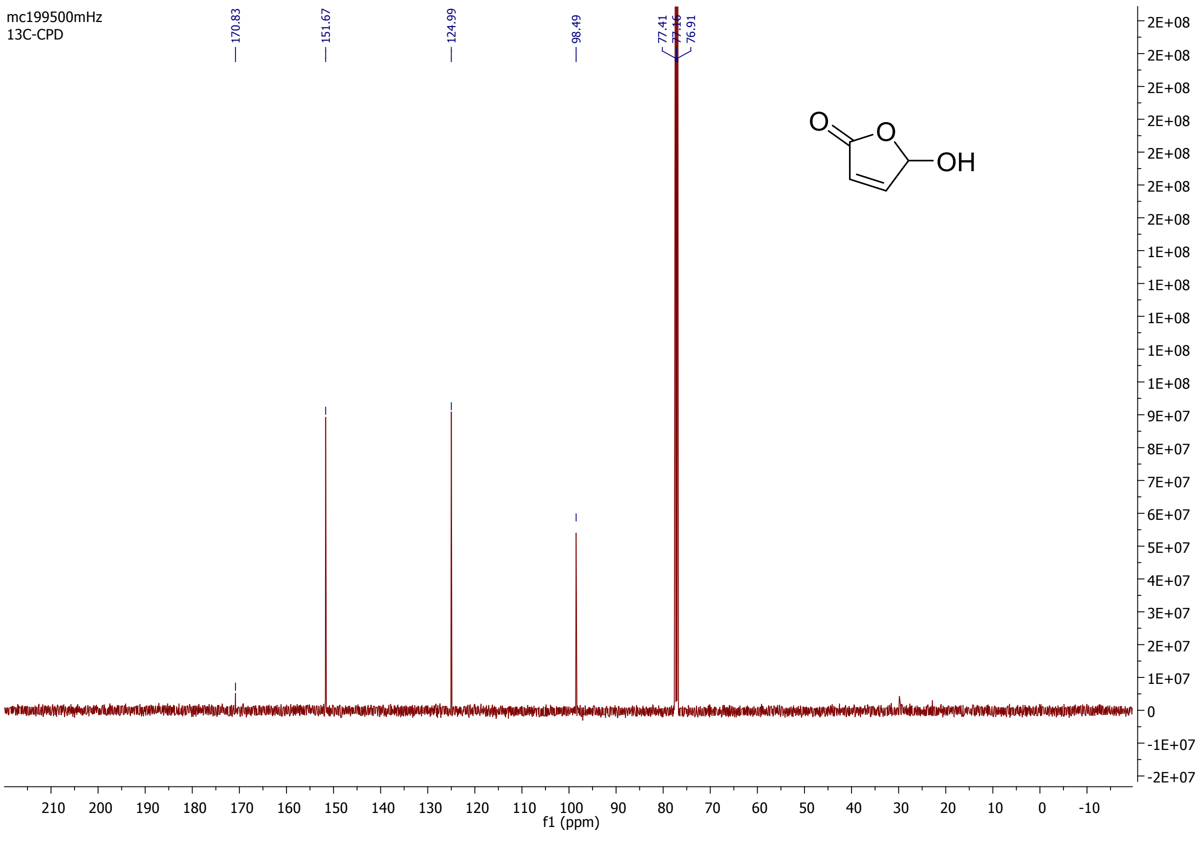

(Z)-1,2,3,4-Tetraphenyl-2-butene-1,4-dione (13)

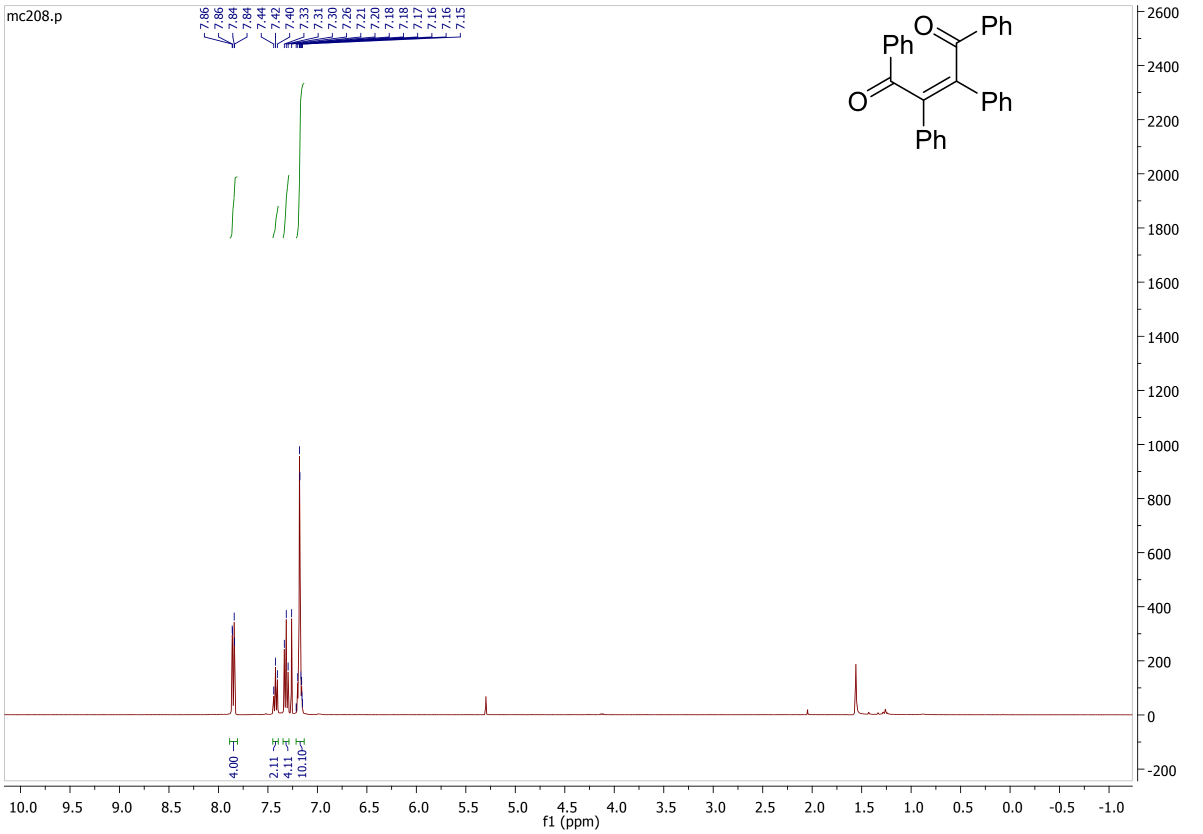

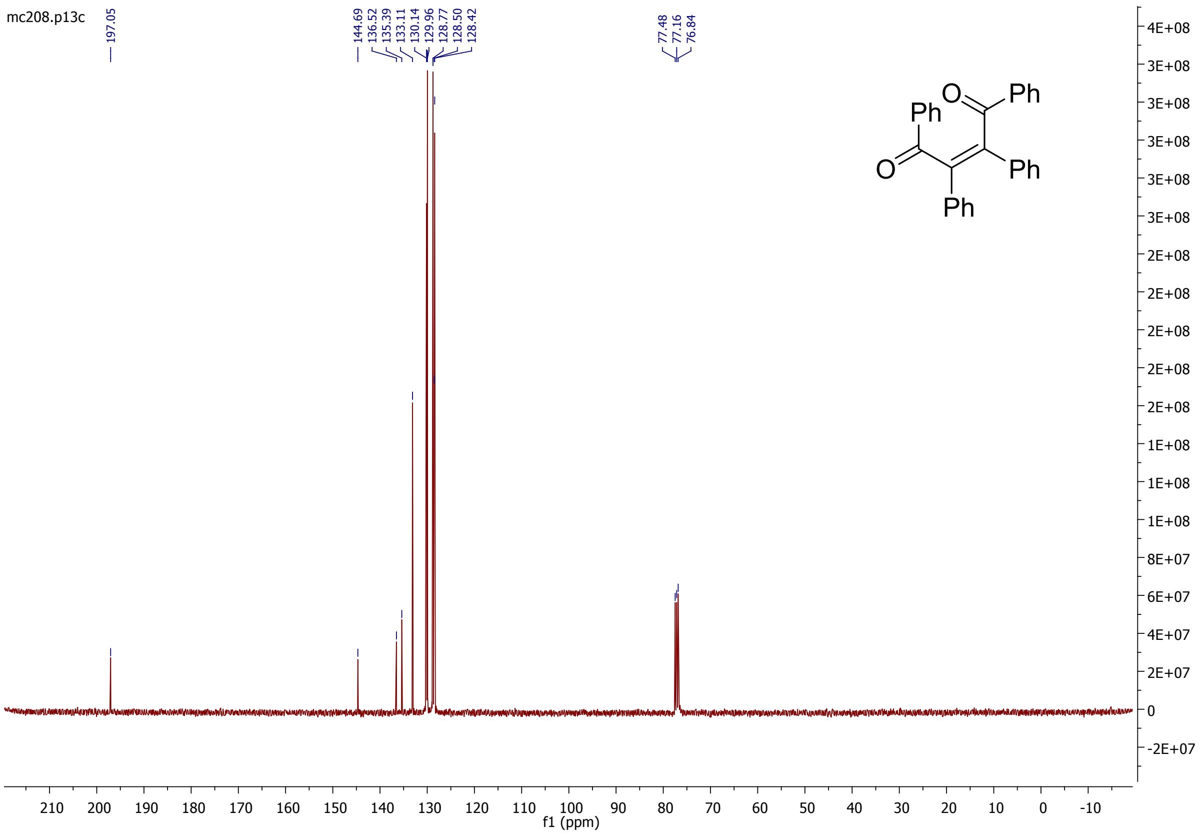

Ethyl 5-phenyl-1H-pyrrole-2-carboxylate (15)

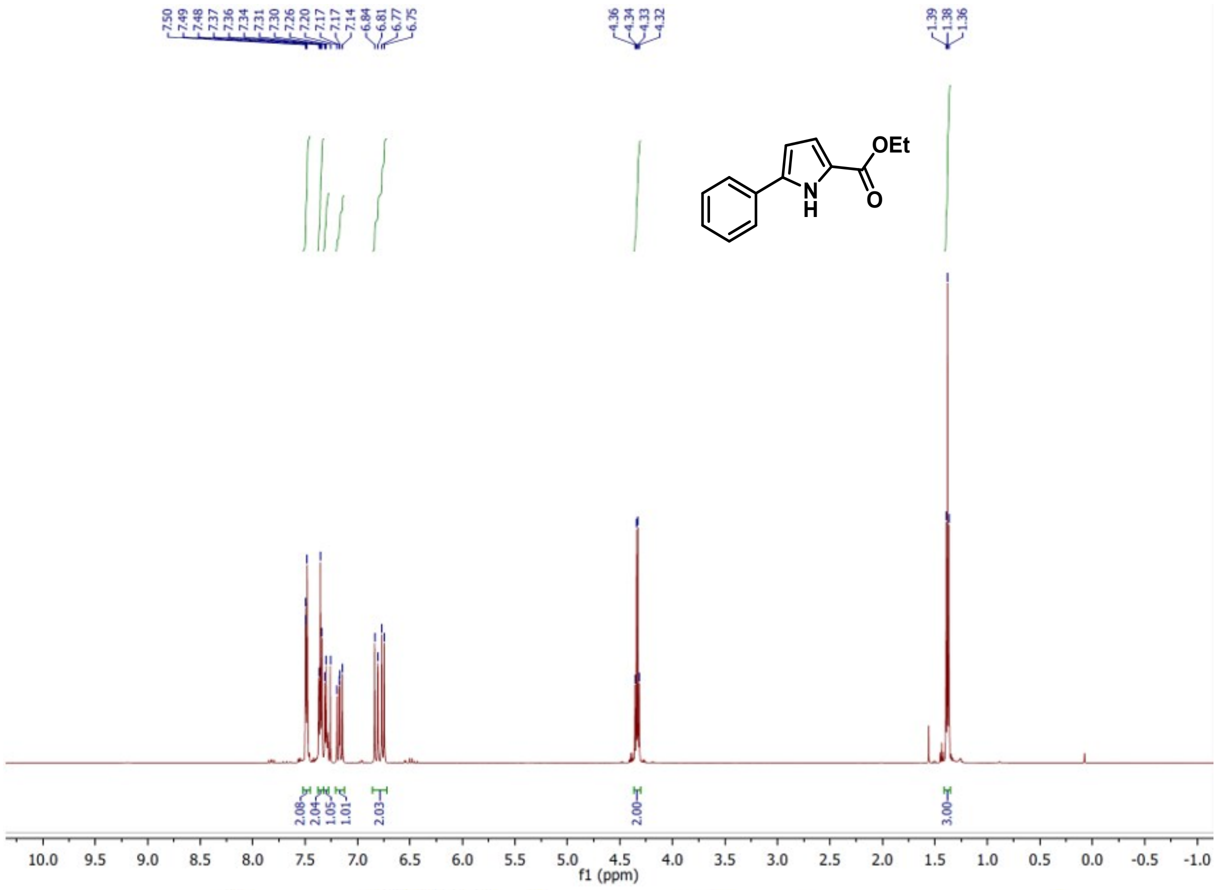

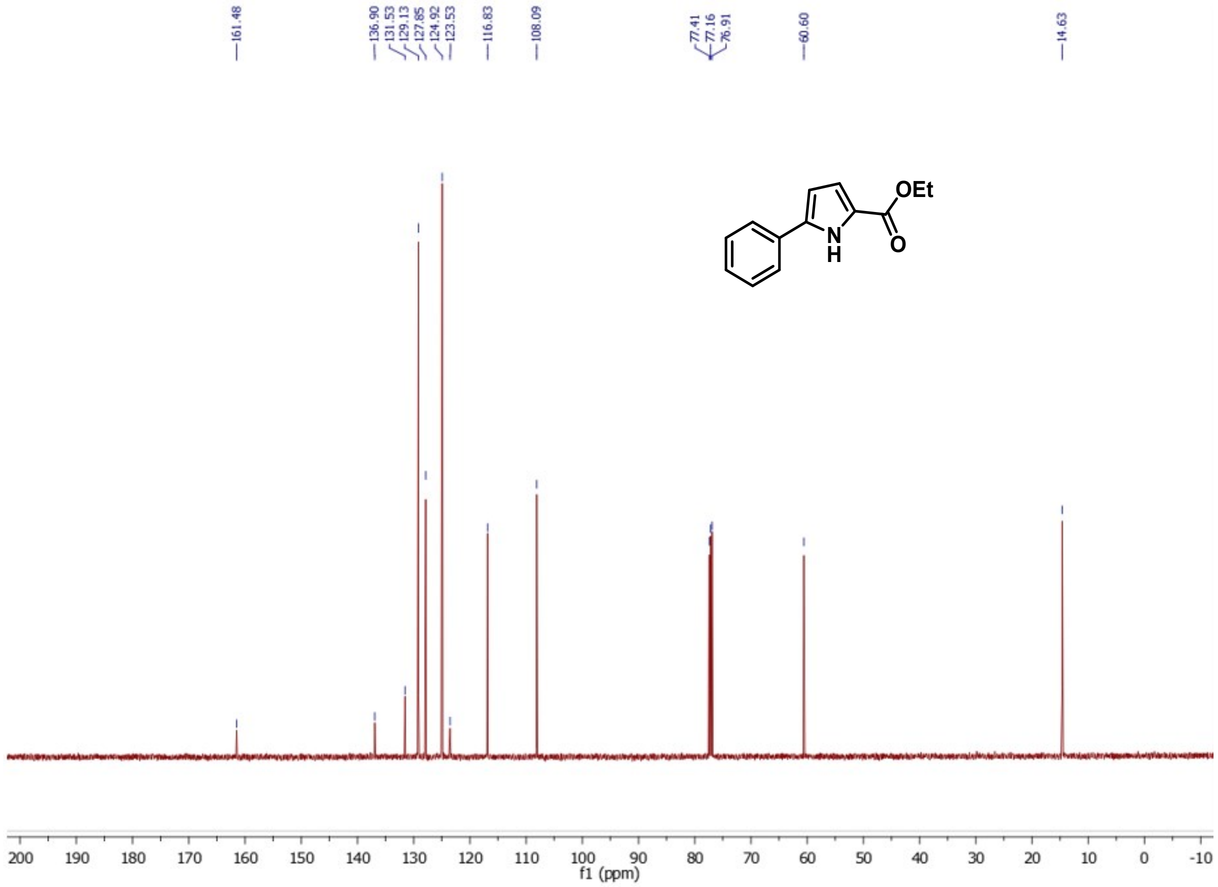

(3,3,3-trifluoroprop-1-ene-1,1-diyl)dibenzene (18)

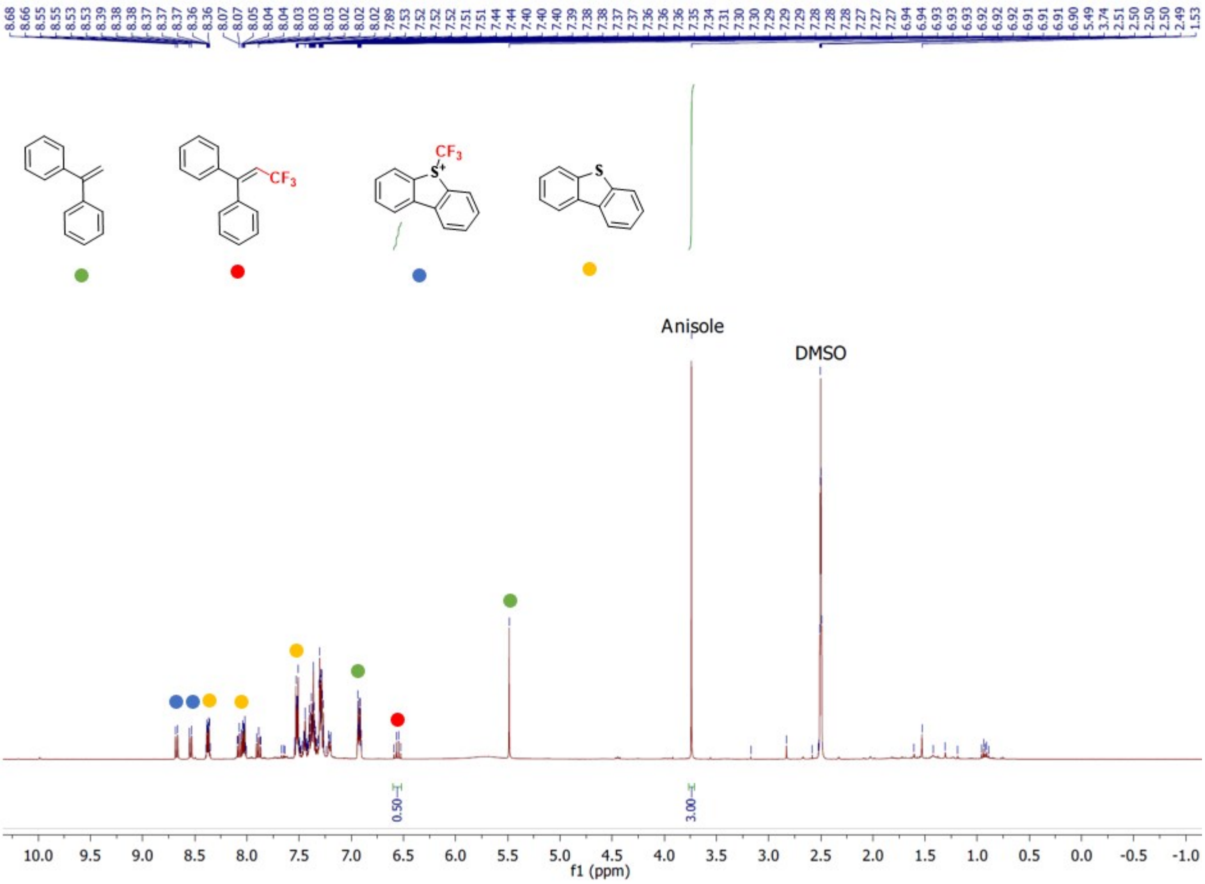

4,4'-(3,3,3-trifluoroprop-1-ene-1,1-diyl)bis(methoxybenzene) (20)

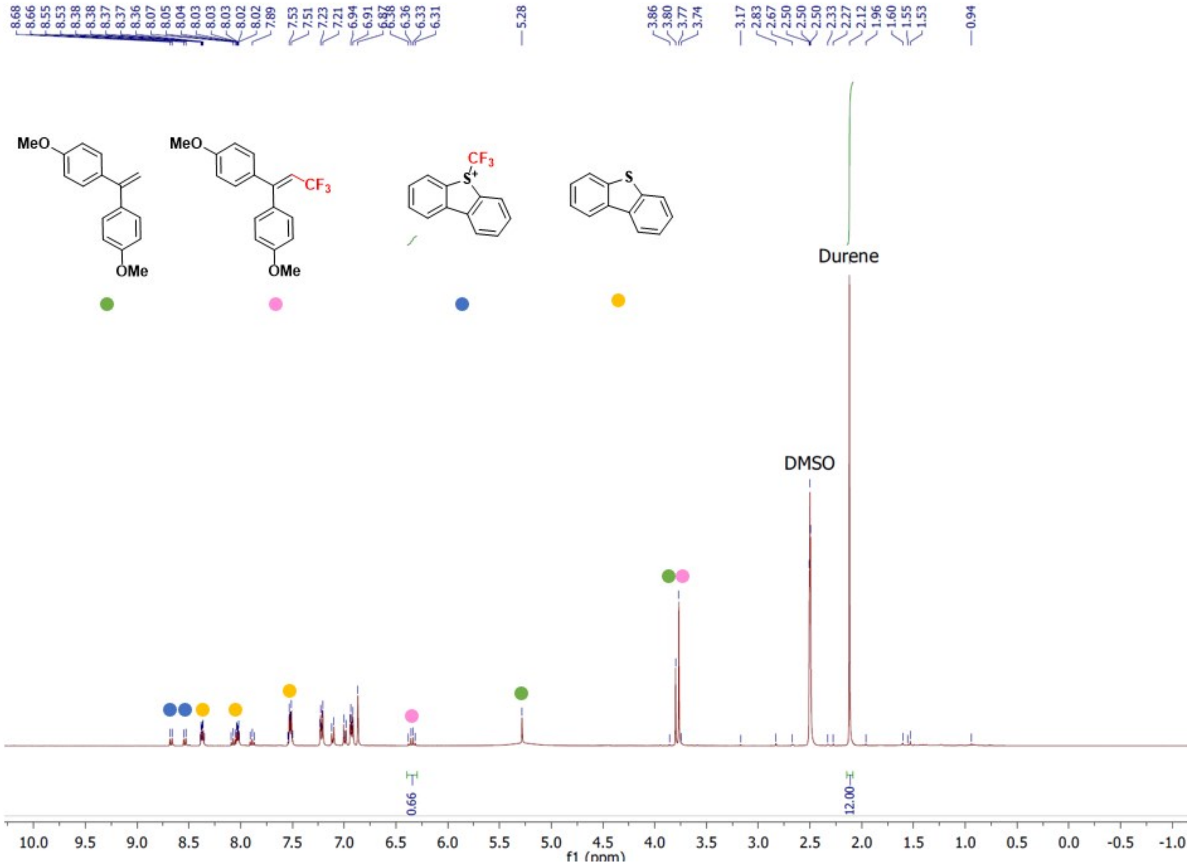

3,3,3-trifluoro-1,1-diphenylpropan-1-ol (21)

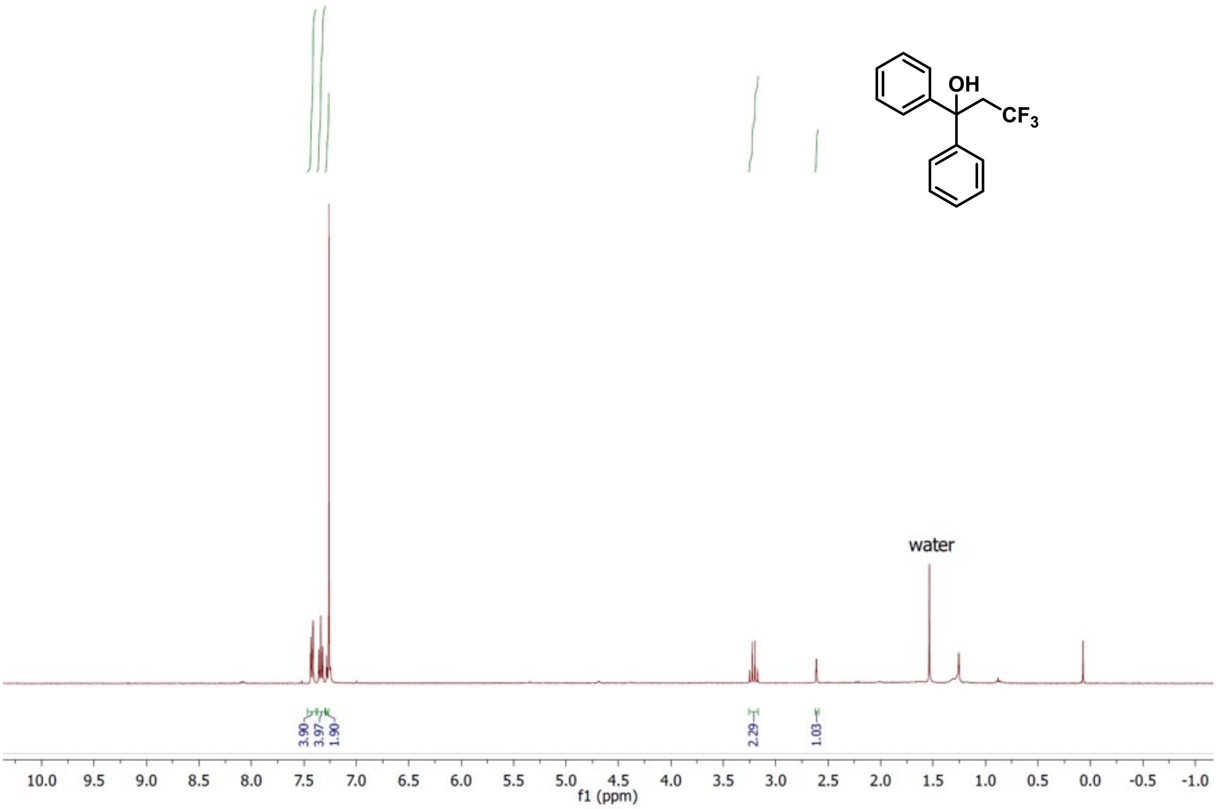

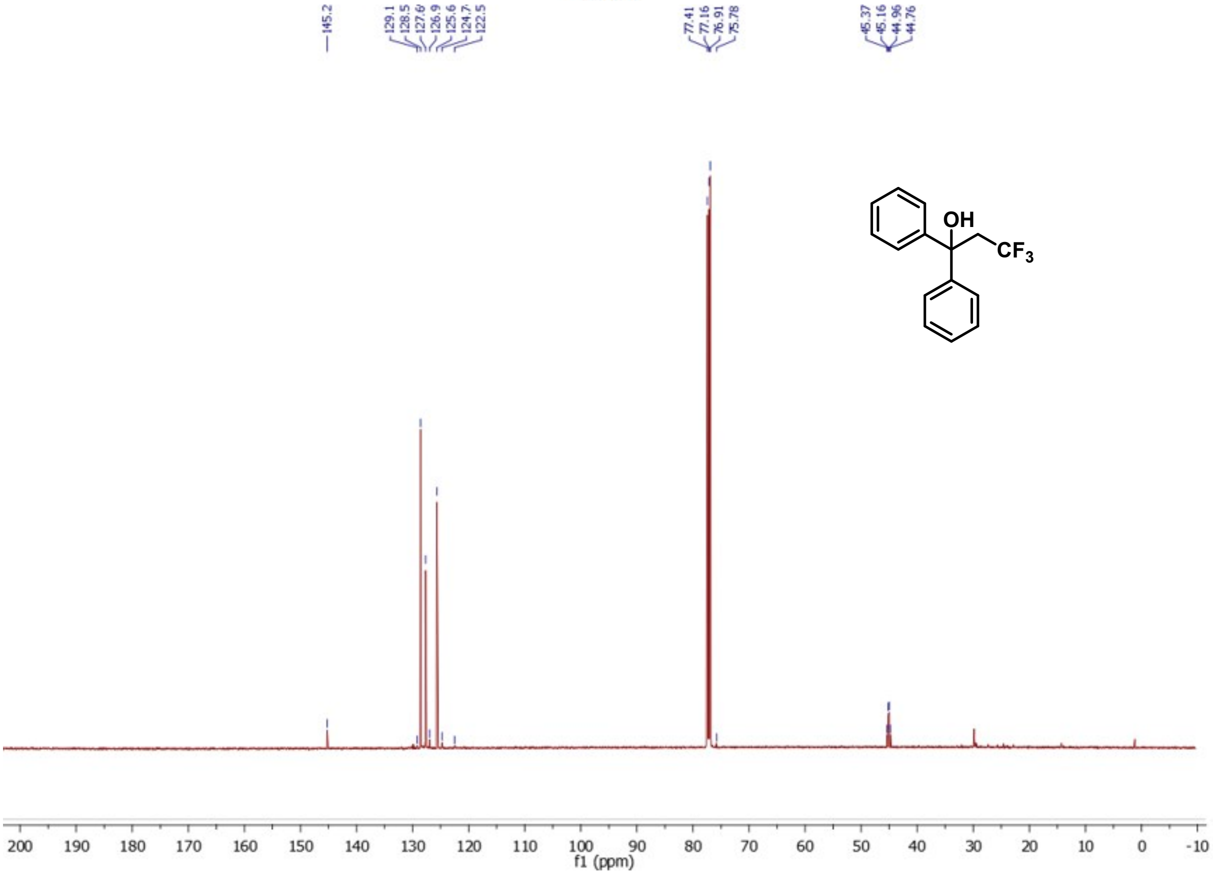

Supplement: SC-012-D1SC00998B-s001 [file SC-012-D1SC00998B-s001.pdf]
